# Bayesian inference of tissue-migration histories in metastatic cancer from cell-lineage tracing data

**DOI:** 10.1016/j.xgen.2026.101193

**Published:** 2026-03-30

**Authors:** Stephen J. Staklinski, Armin Scheben, Lise M. Brault, Rebecca Hassett, Ryan N. Serio, Jiawei Xing, Dawid G. Nowak, Adam Siepel

**Affiliations:** 1 Simons Center for Quantitative Biology, Cold Spring Harbor Laboratory, Cold Spring Harbor, NY 11724, USA; 2 Meyer Cancer Center, Weill Cornell Medicine, New York, NY 10065, USA; 3 Department of Pharmacology, Weill Cornell Medicine, New York, NY 10021, USA; 4 Division of Hematology and Medical Oncology, Department of Medicine, New York Presbyterian Hospital, Weill Cornell Medicine, New York, NY 10021, USA

**Keywords:** cancer metastasis, lineage tracing, Bayesian phylogenetics, single-cell genomics

## Abstract

Cell-lineage tracing now enables direct study of tissue migration in metastatic cancer, but current reconstruction algorithms are limited by a reliance on strong parsimony assumptions and pre-estimated cell-lineage phylogenies. Here, we introduce a probabilistic modeling and inference framework, called Bayesian Evolutionary Analysis of Metastasis (BEAM), which provides richer information about complex metastatic histories. Based on the flexible BEAST 2 platform for Bayesian phylogenetics, BEAM infers a full posterior distribution over cell-lineage phylogenies and tissue-migration graphs, complete with timing information. We show using simulated data that BEAM reliably outperforms current methods for inference of tissue-migration graphs, especially for more complex histories. We then apply BEAM to public datasets for lung and prostate cancer, finding support for distinct modes of migration across clones and reseeding of primary tumors. Overall, BEAM serves as a powerful framework for revealing the modes, timing, and directionality of tissue migration in metastatic cancer.

## Highlights

• BEAM jointly infers cell-lineage trees and tissue-migration histories
• BEAM outperforms existing methods across simulated biological scenarios
• Posterior distributions quantify migration-history uncertainty and dataset limitations
• BEAM infers complex and heterogeneous migration histories across cancer types

## Graphical abstract

**Figure:**
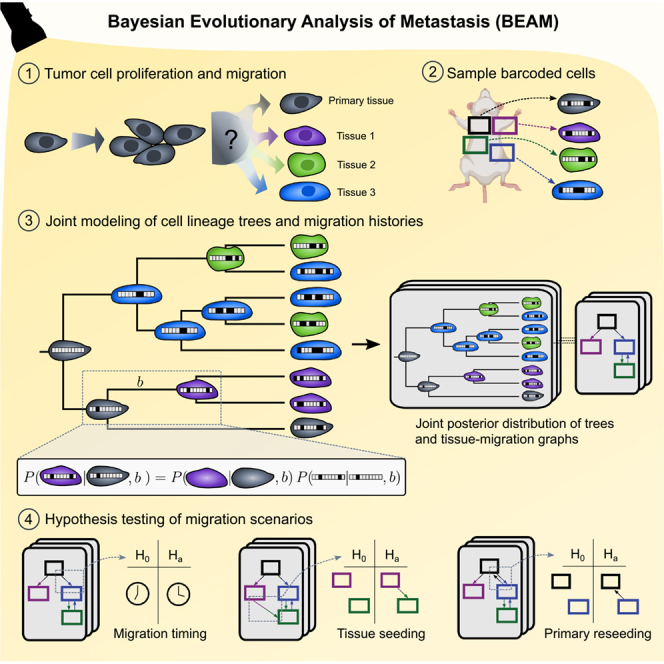

## Teaser

Staklinski et al. present BEAM, a Bayesian approach for uncovering how cancer cells spread between tissues using lineage-tracing data. By accounting for uncertainty and jointly modeling cell lineages and migration, BEAM uncovers complex patterns of metastasis across cancer types and enables hypothesis testing of competing models for how tumors spread.

## Introduction

Like all populations of proliferating cells, tumors adapt to their environments through an evolutionary process involving mutation, selection, and genetic drift.^1^ During the past two decades, the tools of statistical phylogenetics—originally devised to infer species trees^2^^,^^3^^,^^4^—have been adapted for reconstructing tumor evolution.^5^^,^^6^^,^^7^^,^^8^ Recently, technologies for single-cell lineage tracing and sequencing^9^^,^^10^^,^^11^^,^^12^^,^^13^ have inspired phylogenetic methods to reconstruct lineage histories for thousands of individual cells.^14^^,^^15^^,^^16^^,^^17^^,^^18^^,^^19^ These methods have been applied not only to cancer cells but also to problems in developmental biology^20^^,^^21^^,^^22^ and neurobiology.^23^

As these methods have advanced, interest in tumor lineage reconstruction has turned to the critical question of how cancer cells metastasize from one tissue to another. In many cancers, metastasis represents a transition from a localized tumor that can be effectively treated with radiation or surgery to a systemic disease requiring riskier and less effective treatments such as chemotherapy and immunotherapy.^24^ Several common types of cancer—including breast, prostate, colorectal, urinary bladder, and kidney cancer—are highly treatable (with 5-year survival rates >90%) when they are caught before metastasis occurs but have dramatically poorer outcomes (with 5-year survival rates of ∼40% or, in some cases, much less) after metastasis.^25^ The causes of cancer mortality are multifaceted and it is an oversimplification to attribute most cancer deaths to metastasis^26^^,^^27^; nevertheless, metastasis is a key event in the transition from treatable to untreatable disease.^24^

Despite the importance of metastasis, much remains unknown about how and why it occurs. It is not well understood whether metastases are initiated by single or multiple cells, whether tumor cells primarily spread through vascular or lymphatic pathways, how frequently migrations occur, and whether they predominantly arise from the primary tumor or are dispersed across tissues.^28^^,^^29^^,^^30^^,^^31^^,^^32^^,^^33^^,^^34^^,^^35^^,^^36^^,^^37^ It also remains unclear why metastatic tumors appear preferentially in certain organs, in a manner that depends strongly on the cancer type, a phenomenon known as organotropism.^38^^,^^39^^,^^40^^,^^41^^,^^42^ Strikingly, the fundamental question posed by the English surgeon Stephen Paget in his seminal article of 1889—“What is it that decides what organs shall suffer in a case of disseminated cancer?”^43^—still has no good answer.

As shown in recent studies,^34^^,^^37^^,^^44^ the combination of cell-lineage tracing and phylogenetic reconstruction, typically in mouse models, promises to shed new light on these long-standing puzzles. These methods can reveal critical aspects of the rates, routes, drivers, clonality, and specific molecular changes associated with metastatic events. Notably, the primary object of interest in these studies is typically not the cell-lineage phylogeny itself but rather the induced history of migration events, which can be summarized as a “migration graph”^45^—a collapsed version of the phylogeny with nodes representing tissues and (directed) edges representing migration events. If both the phylogeny and the tissue of residence of each cell are known, then the migration graph is simply derived by traversing the branches of the tree and recording a corresponding migration event if the parent cell and child cell occupy different tissues. The challenge is that neither the tree nor the tissues of ancestral cells are known, and uncertainty about these objects propagates to the migration graph in a complex manner. Interestingly, the problem of inferring the tissue-migration graph is related to the problem of phylogeographic reconstruction of the movements of ancestral species.^46^^,^^47^^,^^48^ In both cases, the phylogeny provides a guide but it is the induced migration history that is of primary interest.

Current methods for reconstructing tissue-migration graphs follow a two-step process in which one lineage phylogeny is first reconstructed, and then a migration history is inferred conditional on that phylogeny.^45^^,^^46^^,^^49^^,^^50^^,^^51^ Typically, many migration histories are compatible with a given phylogeny, so one is selected by maximum parsimony, i.e., by minimizing the number of tissue migrations, possibly together with other criteria. This approach is practical and leverages new tools for cell-lineage reconstruction, but it fails to exploit the tightly coupled nature of the problems of inferring the lineage tree and the migration graph. In particular, there are often many lineage trees compatible with the data, particularly when mutations are sparse, but some of those trees may suggest more likely migration histories. Ideally, the two problems would be solved together so that the impact on migration histories could be considered when inferring the lineage phylogeny (see El-Kebir et al.^45^ for an early attempt at a joint solution).

A related problem is that, even when the lineage tree is fixed, many different migration histories are often plausible, and biological questions of interest are typically best addressed by considering a collection of possible histories rather than one of them.^49^ For example, cancer biologists may be primarily interested in a higher-level question such as whether metastasis to bone is more likely than metastasis to the liver or how frequently reseeding of the primary tumor occurs. Ideally, an analysis method would permit evaluating the cumulative support for such a question relative to a probability distribution of possible graphs given the data, but no such method yet exists.

We address these limitations by introducing a joint probabilistic model for cell-lineage phylogenies and tissue-migration graphs together with a procedure for Bayesian statistical inference. Our software implementation, called Bayesian Evolutionary Analysis of Metastasis (BEAM), is the first to support inference of the full posterior distribution over lineage trees, tissue-migration graphs, and associated parameters. BEAM is implemented using the BEAST 2 (Bayesian Evolutionary Analysis by Sampling Trees 2) platform for Bayesian phylogenetics,^52^ and it leverages BEAST 2’s optimized procedures for Markov chain Monte Carlo (MCMC) inference of phylogenetic parameters. In addition, BEAM supports Bayesian hypothesis testing of any derived property of the migration graph, such as the presence of particular edges. We show using simulated data that BEAM accurately reconstructs features of the true migration graph over a broad range of parameters, consistently outperforming available methods. In addition, we apply BEAM to recently published lineage-tracing datasets for lung- and prostate-cancer models and show that it reveals important migration-graph features that were not evident by maximum parsimony. Overall, we show that BEAM is a promising new approach for dissecting the dynamics of metastasis across diverse cancer types.

## Design

**Figure 1:**
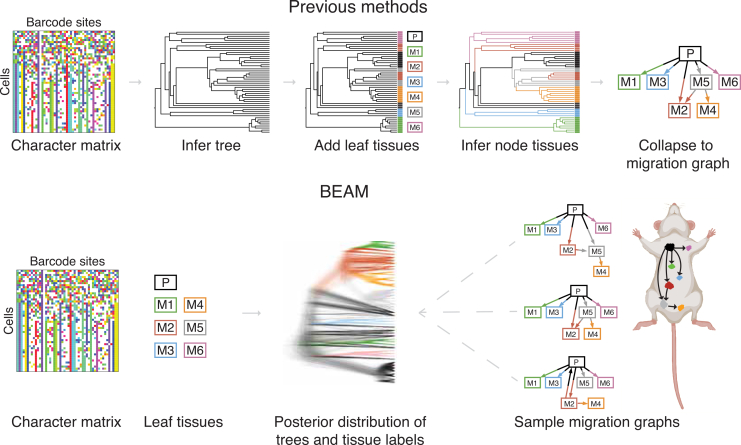
Bayesian inference of lineage trees and tissue-migration histories Existing methods infer a fixed lineage tree from the mutation matrix, assign observed tissue labels to leaves, and reconstruct one or a few migration graphs. By contrast, BEAM jointly models lineage trees and tissue migration, sampling from a joint posterior distribution. In the character matrix, rows represent cells, columns represent barcode sites, and colors are mutations. Tissue labels denote primary (P) and metastatic (M1–M6) tissues. See also Figure S1.

The problem of inferring both the cell-lineage phylogeny and tissue-migration graph can be recast as inferring a “colored” lineage tree, in which each node (cell) is assigned a color representing its tissue of residence (Figure 1). A coloring of nodes unambiguously induces a tissue-migration graph because migration edges correspond to tree branches whose adjoining nodes have mismatching colors. Colors (tissues) of the tips of the tree are generally known from the sampling procedure, so the problem reduces to inferring a coloring of internal nodes.

To define a probabilistic model for this joint process, it is sufficient to extend a model for CRISPR-based lineage tracing to allow for changes in colors (tissues), together with the accumulation of mutations, along branches of the tree. We extended a simplified version of the continuous-time Markov chain (CTMC) model for CRISPR-Cas9 barcode editing implemented in TiDeTree^53^ (see also Chu et al.^19^) with a second, conditionally independent CTMC that describes tissue-migration events. This second CTMC can be parameterized in many ways, but our default is a general time reversible (GTR) parameterization.

With this model, time-dependent transition probabilities along tree branches—representing joint probabilities of mutations and tissue migrations—are products of standard rate-matrix exponentials, and the full likelihood of the data can be calculated using Felsenstein’s pruning algorithm.^54^ While straightforward, this approach enables joint inference of lineage phylogenies and migration graphs by MCMC sampling, avoiding the two-step approximation of existing methods (Figure 1). We implemented this model in a package called BEAM, which is built on the BEAST 2 platform.^52^ An example of the output of BEAM is shown in Figure S1.

## Results

### BEAM recovers tissue-migration histories from realistic simulations

To evaluate the accuracy of BEAM in reconstructing migration histories, we combined existing simulation tools^14^^,^^45^^,^^55^ to model single-cell birth-death dynamics, tissue migration, and CRISPR barcode editing. We first generated synthetic multi-tissue barcode sequence data under a regime favorable for migration-graph reconstruction, with a relatively low migration rate (1 × 10^−6^ per cell) and a relatively high mutation rate (0.0025 mutations per barcode site per cell division). We then assessed the precision and recall of BEAM at predicting individual edges of each simulated migration graph across 100 replicates. For comparison, we also evaluated the MACHINA,^45^ PathFinder,^49^ Metient,^50^ and MACH2^51^ migration-graph reconstruction methods. As a baseline, we assigned tissues (colors) to ancestral nodes by simple Fitch-Hartigan parsimony,^56^^,^^57^ the most common descendant tissue (consensus), or a random tissue (random).

All published methods—except PathFinder, which infers its own phylogeny—require a pre-estimated tree. Our default inputs were phylogenies inferred by maximum likelihood using LAML (Lineage Analysis via Maximum Likelihood).^19^ To evaluate sensitivity to the provided phylogeny, however, we also assessed performance using a tree inferred by Cassiopeia-Greedy under a parsimony assumption^14^ or the ground-truth simulated tree.

**Figure 2:**
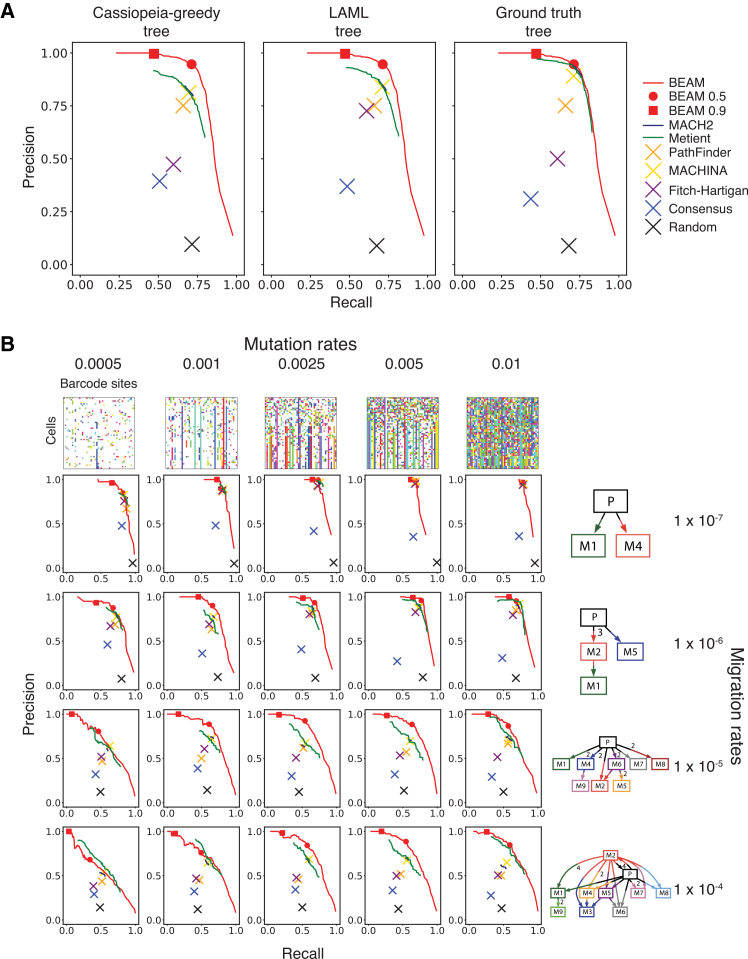
Precision-recall benchmarking across simulated regimes (A) Precision-recall curves for simulated migration graphs (migration rate 1 × 10^−6^; mutation rate 0.0025 per site per division). The BEAM curve is obtained by varying a threshold for the posterior probability of each edge (0.5 and 0.9 thresholds shown as points). Points are shown for the single-solution methods MACHINA^45^ PathFinder,^58^ Fitch-Hartigan parsimony, and two baseline methods (consensus and random; see text). Curves are shown for MACH2^51^ and Metient,^50^ but they are restricted in length. Values represent means over 100 simulations and are conditioned on the input-tree method indicated above each panel. Because PathFinder and BEAM do not require a fixed input tree, their results are identical across panels. (B) Precision-recall curves under varying mutation and migration rates using LAML-based input trees. Representative mutation matrices and migration graphs are shown. See also Figures S2, S3, S4, S5, S6, S7, S8, and S18.

We found that BEAM performed well, achieving nearly perfect edgewise precision (>95%) up to a recall rate of ∼70%, after which the precision declined rapidly (Figure 2A). In this parameter regime, MACHINA, PathFinder, Metient, and MACH2 generally exhibited comparable but slightly less favorable tradeoffs between precision and recall. Notably, however, MACHINA and PathFinder—because they produce a single reconstructed migration graph—only appear as points on a precision/recall graph. Both Metient and MACH2 can report multiple solutions, but we found that they tended to be similar and fall in a narrow range on the graph, more so for MACH2 than Metient. By contrast, BEAM permits a broad range of tradeoffs between precision and recall by allowing the user to choose the threshold for the estimated posterior probability for each edge. All methods outperformed the consensus and random baselines, although the Fitch-Hartigan parsimony approach was sometimes competitive. For comparison with previous publications,^45^^,^^50^^,^^51^ we also computed the F1 score for all methods, using a posterior-probability threshold of 0.5 for each edge in Metient, MACH2, and BEAM (Figure S2). By this metric, the rank order of methods was similar and BEAM maintained a significant advantage.

Notably, the differences in performance between BEAM and other methods became more pronounced when a lower-quality phylogeny from Cassiopeia-Greedy was used as input. Conversely, when other leading methods were given the ground-truth simulated tree, the performance of MACH2 and Metient was comparable to BEAM. Interestingly, the reconstruction accuracy of the trees themselves, as measured by Robinson-Foulds distance, was also highest for BEAM, followed by LAML, and then Cassiopeia-Greedy (Figure S3). Thus, BEAM is able to improve both lineage phylogenies and migration graphs by reconstructing them together. Examples of ground-truth migration graphs and BEAM-inferred graphs are provided in Figure S4.

To see whether these trends continued with other choices of parameters, we carried out experiments with barcode-mutation rates ranging from 0.0005 to 0.01 mutations per site per cell division and migration rates ranging from 1 × 10^−7^ to 1 × 10^−4^ per cell, executing 20 replicates for each combination and using pre-estimated phylogenies from LAML as the inputs for other methods. These scenarios cover a range from many mutations and few migrations, yielding well-resolved lineage trees and simple migration histories, to few mutations and many migrations, where histories are more complex and uncertainty about them should be greater.

As expected, the prediction performance of most methods improved with increasing mutation rate and decreasing migration rate (Figure 2B). Not surprisingly, all but the baseline methods performed well at high mutation and low migration rates (top right of Figure 2B) but performed considerably worse at low mutation and high migration rates (bottom left of Figure 2B). Nevertheless, BEAM outperformed all other methods across all parameter values. Interestingly, the performance improvement from BEAM was most pronounced at intermediate-to-high mutation and migration rates (particularly toward the center and bottom-right of Figure 2B), indicating that the advantages of the Bayesian approach were most evident when there was at least a modest amount of phylogenetic signal and substantial uncertainty about migration-graph structure. This may reflect the increased number of metastasis-informative mutations—those occurring on metastasis edges (Figure S5). In this regime, MACHINA, Metient, and MACH2 also improved on Fitch-Hartigan parsimony. When we stratified our simulated data based on features of the underlying ground-truth migration graphs, BEAM maintained performance across variable migration counts (Figure S6A), co-migration counts (Figure S6B), number of tissues (Figure S6C), monoclonal or polyclonal seeding events (Figure S6D), metastatic-to-metastatic seeding events (Figure S6E), and primary reseeding events (Figure S6F). Overall, BEAM performed well across a range of migration dynamics and proved to be robust to variable levels of information content in the data.

Although these analyses examined mutation and migration complexity, they did not capture the impact of indel-driven barcode site loss. To evaluate this effect, we reused the 100 simulations in Figure 2A and regenerated barcode data under increasing heritable silencing rates. In these experiments, BEAM remained the top-performing method across missing-data levels, retaining reasonable accuracy up to ∼90% missing data (Figure S7), likely because it can draw on tissue labels during tree reconstruction when mutations are sparse. In contrast, all other methods degraded markedly at higher missing-data levels.

To understand how BEAM’s predicted migration graphs differed from those reconstructed by parsimony, we carried out a direct comparison on the same trees. We used BEAM to sample lineage trees, and then, for those trees, we calculated both the number of migration events predicted by BEAM and the minimum number possible according to Fitch-Hartigan parsimony.^34^ The difference between these quantities is a measure of the excess migrations predicted by BEAM. Not surprisingly, in favorable regimes for migration-graph reconstruction, this excess was low, with values of zero in most cases and occasional deviations of 1–3 migrations (Figure S8A). With lower mutation rates or higher migration rates, however, we observed larger excesses, with the BEAM predictions having as many as five, and sometimes 15 or more, events beyond the minimum possible (Figures S8B and S8C). Given the clear performance advantage of BEAM in this regime (Figure 2B), this observation implies that parsimony assumptions tend to break down when migration rates are high or mutational information is limited, leading to underestimation of migration events. In contrast, BEAM relaxes those assumptions, allowing the data to inform a broader range of plausible histories.

### BEAM reveals complex tissue-migration patterns in lung and prostate cancer

Having demonstrated that BEAM performed well on simulated data, we turned to real data from two recent applications of lineage tracing in mouse models of cancer metastasis, one in lung^34^ and one in prostate.^37^ Despite differences in their strategies for cancer initiation (an orthotopic xenograft model of lung cancer in immunodeficient mice vs. a somatically engineered mouse model of prostate cancer in immunocompetent mice) and barcode sequencing (single-cell RNA sequencing vs. bulk sequencing of PCR-amplified DNA), these studies both produced mutation matrices representing barcoded cells from distinct tissues. In both cases, cells were grouped by barcode similarity into clonal populations (CPs) representing lineage trees from distinct founder cells.

The two datasets had previously been analyzed using different parsimony strategies (Fitch-Hartigan parsimony in Quinn et al.^34^ and MACHINA in Serio et al.^37^). For a uniform comparison point for BEAM, we reanalyzed them using LAML^19^ (instead of Cassiopeia^14^) for lineage-tree reconstruction and MACH2^51^ for migration-graph inference. The tissue-migration graphs reconstructed by BEAM and MACH2 were broadly similar with somewhat more complexity evident in the BEAM reconstructions on average, consistent with our simulation results. For example, in the 0.5-posterior-probability threshold graphs across lung-cancer CPs, MACH2 averaged 12.8 migrations and 3.5 co-migrations, while BEAM averaged 13.9 migrations and 4.6 co-migrations; across prostate-cancer CPs, MACH2 averaged 8.2 migrations and 2.8 co-migrations, compared to BEAM averages of 16.6 migrations and 5.4 co-migrations. When we examined individual CPs, we found that, in some cases, the two methods predicted quite different evolutionary histories (e.g., Figure S9), but in other cases the predictions were similar (e.g., Figure S10). CPs varied considerably in size, mutation content, and migration-graph complexity between the two datasets, providing diverse scenarios to evaluate BEAM.

**Figure 3:**
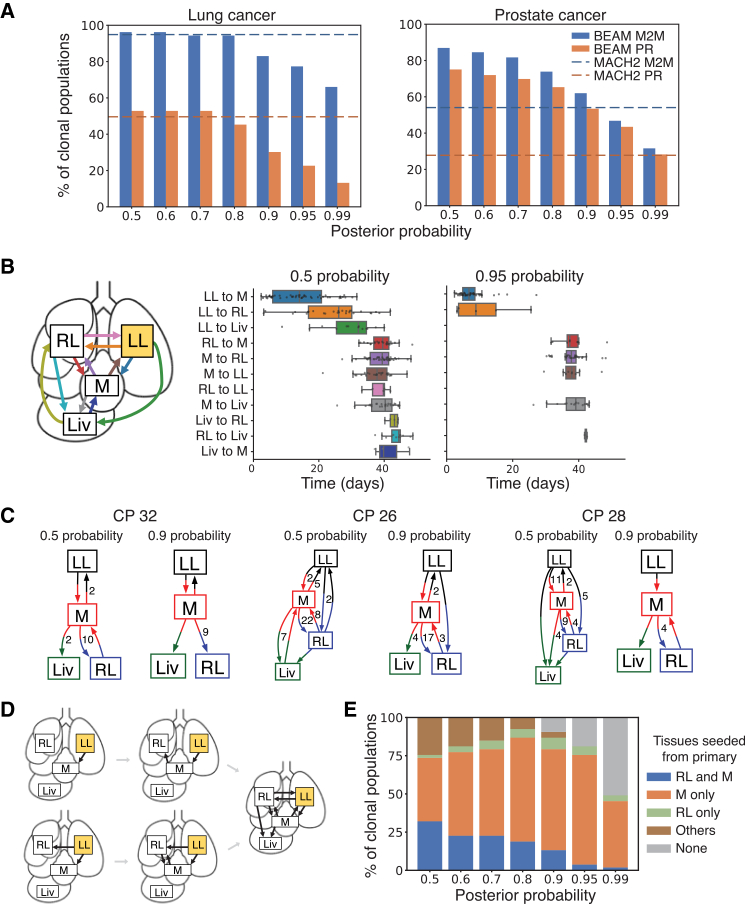
Metastatic progression patterns in lung and prostate cancer (A) Fractions of clonal populations (CPs) with detected metastasis-to-metastasis (M2M) or primary reseeding (PR) events by BEAM across edgewise posterior-probability thresholds for lung^34^ (left) and prostate^37^ (right) datasets. MACH2^51^ estimates (threshold invariant) are shown as dashed lines. (B) BEAM-estimated migration times in the lung-cancer data at 0.5 and 0.95 edgewise posterior-probability thresholds. Boxplots show distributions of expected migration times per edge type per CP, averaged over posterior samples of branch midpoint times. (C) Example 0.5 and 0.9 threshold graphs for lung CP32, CP26, and CP28, illustrating M-hub and direct left lung (LL) → right lung (RL) models. Edge labels indicate multiplicity (>1 lineage-tree branches). (D) Representative progression of mediastinum (M)-hub and direct LL → RL models. (E) Classification of all lung-cancer CPs as posterior-probability threshold increases: LL → RL and LL → M (RL and M), M only (LL → M), RL only (LL → RL), and finally whether LL seeded any other tissues (others or none). Liv = liver. See also Figures S9, S10, S11, S12, and S13 and Table S1.

A key open question is whether migration events predominantly arise from the primary tumor or whether, by contrast, migrations occur at appreciable frequencies between metastatic sites or from metastatic sites back to the primary tumor. The original lung-cancer analysis^34^ reported high rates of both metastasis-to-metastasis (M2M; ∼90%) and primary reseeding (PR; ∼65%) events. BEAM and MACH2 were reasonably consistent with the previous analysis but with lower estimates of the incidence of PR events: MACH2 reported ∼95% M2M and ∼50% PR, and, at a posterior-probability threshold of 0.8, BEAM reported ∼94% and ∼45%, respectively (Figure 3A). Notably, however, the BEAM predictions were quite sensitive to the edgewise posterior-probability threshold, with the rate of M2M events varying from ∼96% at a threshold of 0.5 to ∼66% at a threshold of 0.99, and the rate of PR events varying from ∼53% to ∼13%. MACH2 typically produced a small set of similar graphs, making results largely insensitive to the edgewise posterior-probability threshold. Overall, our Bayesian framework appears to assign high uncertainty to these secondary migration events, which generally occur later in tumor development and have weaker support in the mutation matrix. In particular, our most conservative estimates of the rate of PR events, at ∼13%–23%, are several times lower than the previous estimate of ∼65%. Nevertheless, our analysis supports that both M2M and PR events occur with non-negligible frequency.

The prostate-cancer data differed in several ways from the lung-cancer data. For various reasons—including the use of immunocompetent mice, a DNA readout, and a longer-duration experiment (up to 60 weeks) with more opportunity for drop-out of mutated cells—many fewer barcode mutations are captured in this system, making the phylogenetic inference problem more challenging. In addition, a more diverse set of organs was sampled in this study, including the prostate, liver, lungs, bones, bladder, and lymph nodes, whereas the lung-cancer data were dominated by three organs in close proximity (right lung [RL], left lung [LL], and mediastinum [M]). In part for these reasons, and likely also owing to some differences in their analysis pipeline, Serio et al.^37^ detected M2M and PR events in only ∼7% and ∼0.3% of CPs, respectively (by our re-analysis of their migration graphs).

In our reanalysis, both BEAM and MACH2 detected substantially higher rates of M2M and PR events in the prostate-cancer dataset (Figures 3A and S11). MACH2 found M2M events in ∼54% of CPs and PR events in ∼28% of CPs. At a posterior-probability threshold of 0.8, BEAM estimated even higher frequencies of ∼74% and ∼65%, respectively. As with the lung-cancer data, however, BEAM’s estimates depended strongly on the choice of threshold, declining from ∼87% to ∼32% for M2M and from ∼75% to ∼28% for PR as the threshold increased, indicating that many of the M2M and PR events had weak support in the data. As discussed in the next section, these estimates are undoubtedly influenced by limits in the mutational information available in this dataset, but overall they suggest that the previous analysis may have been somewhat conservative in detecting secondary migration events.

One advantage of our Bayesian inference approach is that it naturally allows for estimation of the times at which tissue-migration events occur, within the limits of a molecular-clock assumption. When applying BEAM migration timing analysis to the lung-cancer dataset, we noticed that the primary LL tissue was often predicted to seed the lymphatic M and RL tissues early in disease progression (Figure 3B). In further examination of the inferred migration graphs, we found that early migrations followed one of two distinct patterns: one in which M was the only site seeded from LL and acted as a hub for all other migrations and another in which both M and RL were seeded from LL (Figure 3C). Here, we used simulated data to show that BEAM more accurately detects the M-hub migration pattern than other methods and infers relative migration rates that are consistent with expectations (Figure S12). Although the early dynamics differed between these patterns, both eventually resulted in widespread dissemination (Figure 3D). The proportion of CPs in each pattern changed with the posterior-probability threshold, but nearly all CPs fit into one of the two (Figure 3E). We also observed that metastasis to the liver (Liv) was most likely to occur via M, rather than from LL or RL (Table S1). Overall, these patterns were broadly consistent with a previously reported principal-components analysis,^34^ but BEAM provided additional information about migration event timing, further revealing the patterns by which these tumors spread.

### Bayesian hypothesis testing of higher-level questions in metastasis

The previous section illustrates a recurring issue: while methods generally produce full tissue-migration graphs, investigators are often interested in higher-level questions, such as the frequency of M2M or PR events, or whether or not the M acted as a migration hub. We extended BEAM to address questions of this kind by averaging over possible migration graphs and weighting them by their posterior probabilities using the framework of Bayesian hypothesis testing. In this way, particular hypotheses of interest can be tested without relying on one or a few reconstructed migration graphs, each with high levels of uncertainty.

To illustrate the utility of this approach, we returned to questions that arose in our reanalysis of the lung and prostate-cancer datasets. First, we sought to test whether or not the prostate-cancer dataset supported the hypothesis of PR, given the discrepancies between methods and across BEAM posterior distributions. In this case, however, we had the additional challenge of sparse mutational data, owing to constraints of the prostate-cancer mouse model. Indeed, we observed that a majority of the CPs exhibited either no phylogenetically informative mutations or similar numbers to the lowest mutation-rate category in our simulations, where inference was challenging (left of Figure 2B). The lung-cancer data was more informative by this measure but still on the low end of our simulated mutation-rate categories. We observed similar limitations in mutation content in additional published datasets for metastatic pancreatic^44^ and lung^35^ cancer (Figure S13), suggesting that generating enough mutations to enable robust migration-graph inference remains a general challenge in the field.

**Figure 4:**
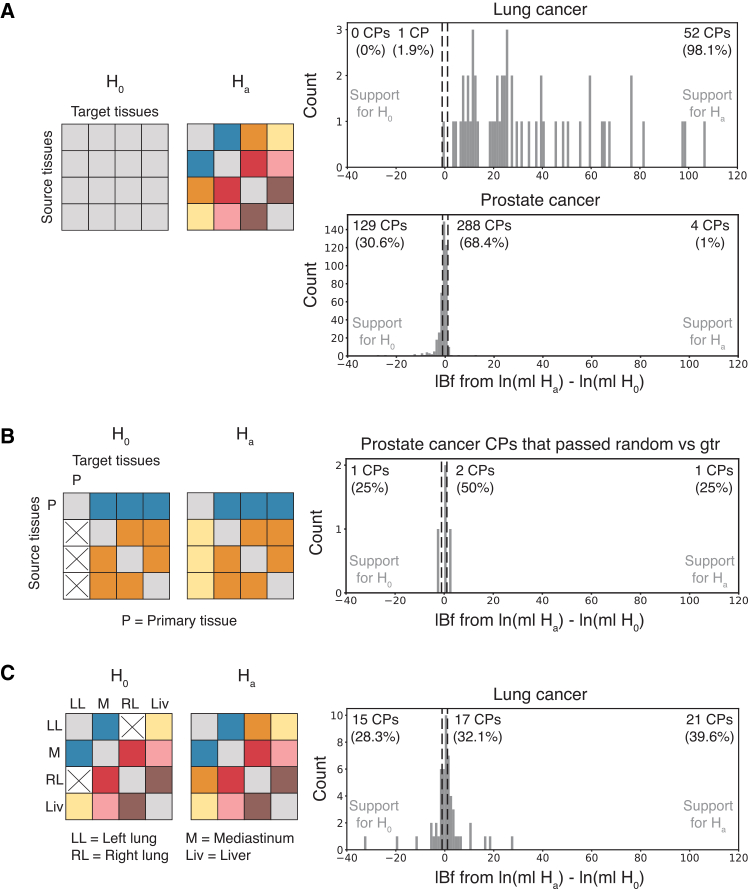
Bayes-factor testing of dataset informativeness and migration structure (A) Hypothesis test of tissue-migration information comparing an alternative GTR model (right) against a random null model (left) in the lung and prostate datasets. (B) Test of a model allowing PR (right) against a null model prohibiting it (left) in prostate CPs that passed (A). (C) Test of a full GTR model allowing all migration types (right) against a null model prohibiting direct LL → RL seeding (left). CPs were classified by log Bayes factor (lBf) as supporting the null, the alternative, or neither, using thresholds lBf <−1.1 and lBf >1.1 (vertical dashed lines). See also Figures S13, S14,S15, S16, and S17 and Table S2.

We therefore defined an initial hypothesis test to distinguish CPs that were sufficiently informative for inference of tissue-migration histories from ones that were not. For this test, we compared an alternative model with a GTR tissue-migration model to a null model in which tissue labels were randomly sampled in proportion to their relative frequencies at the leaves of the tree. This test evaluates whether or not the tissue labels evolve in a Markovian manner along the branches of the tree—that is, whether or not each cell’s tissue label depends on the label of its parent and the branch length between them. If such a dependency exists, then there is at least some information in the data about the tissue-migration process, whereas, if it does not exist, no such information is present. When we applied this test to our simulated data, we found that it was minimally restrictive, allowing all but a few datasets to pass at a threshold of log Bayes factor (lBf) >1.1 (Figure S14). Similarly, the test identified ∼98% of the lung-cancer CPs as informative about tissue migration. When we applied it to the prostate-cancer dataset, however, we found that only four CPs (∼1%) passed (Figure 4A; Table S2), indicating that the limited mutational content in this dataset permits only a minority of CPs to be informative about migration history. These results aligned with a simpler measure of mutual information derived from the full posterior distributions (Figure S15). Similarly, only three pancreatic and two additional lung-cancer clones passed this test (Figure S16).

We then focused our hypothesis testing of PR on the four prostate-cancer CPs that were most informative about migration history. In this case, we tested an alternative model allowing for PR against a null model in which PR events were forced to have rates of zero. We found positive support for PR in only one of the four CPs (Figure 4B), which, interestingly, came from a different mouse than the single case of PR previously identified using MACHINA.^37^ As validation, we performed the same hypothesis test on simulated datasets with and without reseeding events, and we found that all simulations with support for PR did indeed exhibit PR, while 75% of the simulations supporting the null hypothesis did not exhibit PR (Figure S17). Among the simulations without positive support in either direction, 50% exhibited PR, suggesting that our test is conservative. Thus, our Bayes-factor analysis does support PR in the prostate-cancer model, albeit in a small fraction of CPs. Notably, the Bayesian hypothesis test is considerably more stringent than simply applying a posterior-probability threshold to the edges of the migration graph, as in the previous section.

For a second illustration, we designed a hypothesis test to distinguish between the two main patterns we observed in lung cancer. We tested an alternative hypothesis in which LL → RL events were allowed (corresponding to the blue bars in Figure 3E) against a null hypothesis in which they were not, in which case we presume the data will be explained by the M-hub model (orange bars in Figure 3E). This test compared a fully parameterized model (representing the alternative hypothesis) against a model forced to have a rate of zero for LL → RL events (representing the null hypothesis). We found that the alternative hypothesis of direct LL → RL seeding obtained positive support in ∼40% of eligible CPs, whereas the null hypothesis was positively supported in ∼28% of CPs (Figure 4C). The remaining ∼32% supported neither model. This analysis further supports the finding that both the LL → RL and M-hub patterns are present in the data. It additionally suggests that somewhat more CPs exhibit the LL → RL than the M-hub-only pathway.

## Discussion

In this article, we introduced BEAM, a fully Bayesian framework that jointly models cell-lineage and tissue-migration histories to reconstruct the timing and routes of metastatic progression from single-cell lineage-tracing data. To evaluate BEAM, we developed a data simulator that integrates an agent-based model of cancer metastasis with CRISPR-based lineage recording in DNA barcodes. We found that BEAM consistently recovered simulated migration histories with higher precision and recall than existing methods and that the Bayesian approach uniquely allowed for control over the confidence associated with each migration event. In real data for lung and prostate cancer, BEAM uncovered complex and highly connected migration graphs even under stringent posterior-probability thresholds. In lung cancer, we identified two distinct patterns of metastatic progression driven by early divergence and found that liver metastases were preferentially seeded by secondary passage from a lymphatic tumor. In prostate cancer, BEAM highlighted diffuse posterior distributions over migration histories, which we traced to phylogenetic uncertainty resulting from limited mutational information. In both cases, BEAM effectively distinguished between signal and noise, preserving meaningful structure when data were informative while avoiding overfitting when they were not. We also showed that BEAM enabled direct hypothesis testing of features of the migration model, using it to assess the informativeness of lineage data, test for metastatic-to-PR events, and classify progression patterns.

Overall, BEAM makes three contributions to migration-history-inference methods. First, it simultaneously addresses the intertwined problems of lineage-tree and migration-graph inference, enabling lineage trees to be evaluated in part by the likelihood of their induced migration graphs. Second, BEAM samples from a full posterior distribution over migration graphs, which supports decision-making based on posterior-probability thresholds and avoids overconfidence in cases with weak data. We recommend edgewise posterior-probability thresholds of 0.50 for exploratory summaries and 0.90 for conservative results, corresponding to points shown on the plots for simulated data. Third, BEAM supports formal hypothesis testing by marginalizing over tree topologies and phylogenetic parameters, allowing rigorous evaluation of questions about migration structure, data informativeness, and specific evolutionary events. Notably, BEAM is also modular by design, making it adaptable to new lineage-tracing technologies as they evolve. Together, these features make BEAM a powerful framework for extracting meaningful insights from complex single-cell lineage-tracing data.

In our reanalysis of available lineage-tracing datasets, sparse mutation information was a persistent barrier to robust inference of tissue-migration histories (see also Salvador-Martínez et al., Jiang et al., and Gao and Feder^59^^,^^60^^,^^61^). This limitation was especially notable with the prostate-cancer dataset,^37^ which derived from a system designed to maximize biological realism at some expense to mutation rates. Nevertheless, we found that even more mutation-rich datasets for lung^34^^,^^35^ and pancreatic^44^ cancer compared unfavorably with simulated data regimes in which migration graphs could be reconstructed accurately. We conducted extensive simulations spanning a range of mutation and migration rates, allowing readers to assess where future datasets may fall within these regimes. Developing improved bar-coding techniques is a highly active area of research^62^^,^^63^^,^^64^^,^^65^^,^^66^ (see Askary et al.^67^ for a recent review), and we expect that the problem of sparse information will fade in importance as experimental methods improve. At present, however, it is critical for investigators to ensure that reconstructed migration histories are well supported by the data before drawing strong biological conclusions from them. To our knowledge, the Bayes-factor-based test proposed here is the first formal statistical test for this purpose, and we anticipate that it and tests like it will be important in ensuring that migration-graph inference is well grounded in the available data.

An important distinction between BEAM and multi-criteria parsimony approaches is that BEAM does not distinguish single-cell migration events from multiple-cell co-migrations. El-Kebir et al.^45^ introduced the idea of explicitly modeling co-migrations to address the problem that polyclonal events can cause the number of migrations to be over-estimated by standard methods. Their co-migration-aware formulation of the parsimony problem, however, is challenging to solve and led them to employ a computationally intensive mixed integer linear programming approach (see also Koyyalagunta et al. and Roddur et al.^50^^,^^51^). In principle, it would be possible to model co-migrations in BEAM, but a good deal of additional complexity would be required. Instead, we chose simply to model all migration events as occurring independently. Interestingly, even though co-migrations were frequent in our simulations, BEAM performed well in recovering the true migration histories, suggesting that explicit modeling of co-migrations might not be necessary, provided a model is sufficiently flexible in other respects. Despite these encouraging results, future work could explore an extension of the Bayesian approach that models co-migrations. One interesting application would be to evaluate the degree to which the data support the hypothesis of co-migration.

Modeling co-migrations is closely tied to a debate in the field surrounding polyclonal vs. monoclonal metastatic seeding. Early studies favored monoclonal seeding,^68^^,^^69^ whereas recent studies supported polyclonal seeding, with clones disseminating simultaneously or sequentially.^28^^,^^31^^,^^45^^,^^70^^,^^71^^,^^72^^,^^73^^,^^74^ Nevertheless, polyclonal seeding does not always dominate.^70^^,^^75^ For example, a recent genomic study uncovered homogeneous, monoclonal metastatic sites for several cancer types.^42^ However, experimental sampling methods can introduce classification bias.^76^ Metastasis stage and therapy also influence observations,^28^^,^^30^^,^^32^^,^^70^^,^^75^^,^^77^^,^^78^^,^^79^ with distant or post-therapy metastases favoring monoclonal origins.^30^^,^^78^^,^^80^ In cell-lineage tracing, both monoclonal and polyclonal origins were observed even when inferred using criteria that favored co-migrations.^81^ Evidently, both tissue-seeding modes occur across disease settings, calling for flexible modeling strategies that can accommodate both.

Another major source of computational complexity in methods that infer a migration graph by parsimony based on a given lineage tree is the presence in the starting tree of polytomies, or nodes with more than two children. Polytomies reflect uncertainty about the structure of the tree and are common in trees estimated from lineage-tracing data when mutations are sparse. To find parsimonious migration graphs, these methods have to consider the set of all possible refinements of polytomy-containing trees into binary trees, a set that grows exponentially with the number of polytomies. In practice, trees with many polytomies, such as those from the prostate-cancer dataset we analyzed, can lead to extremely long running times. BEAM circumvents this problem by directly exploring the space of tissue-labeled binary lineage trees and considering their likelihoods under both the barcode-mutation and tissue-migration models. As a result, it can avoid regions of the solution space where the lineage tree fits the mutation data but implies an unlikely migration graph, improving efficiency.

Despite these advantages, BEAM has similar limits in scalability to the other available migration-history-inference methods. On one hand, BEAM benefits from simultaneous consideration of lineage trees and migration graphs, native handling of binary trees, a simple model of migration, and relatively fast likelihood calculations. On the other, its reliance on exploration of the full space of tissue-labeled lineage trees by MCMC prohibits it from scaling to thousands of cells. BEAM’s precise scaling limits depended on several factors, including tree size, mutation data, and observed tissues, but we found that it could typically resolve lineage trees with up to 300 cells and was generally impractical to apply beyond that scale (Figure S18). In our hands, these limits were broadly comparable to those of the parsimony-based approaches MACHINA and Metient and higher than that of PathFinder, despite major differences between algorithmic strategies, suggesting current limits of the field that will require new innovations for major improvements in scalability (see Schmidt and Raphael^81^ for one recent example). We note, however, that Metient has been optimized to use GPU resources for improved scalability but that we did not use them in this study. Building on our recent work replacing MCMC with variational inference in Bayesian phylogenetics,^82^ we plan to extend this approach to tissue-migration modeling, as in BEAM, to improve scalability.

Although BEAM could, in principle, be extended to analyze bulk human tumor sequencing data for reconstructing metastatic seeding histories, doing so requires integrating allele-frequency-based mixture models of clonal evolution rather than the discrete sequence-based substitution models currently employed. Developing such extensions represents a substantial methodological shift beyond the scope of the present study.

Beyond modeling tissue migration, the Bayesian approach here is applicable to any discrete-state process evolving along a lineage tree, including cell-state transitions. In principle, BEAM could be adapted to infer cell-state hierarchies from lineage-tracing data, as in recent work.^35^^,^^83^^,^^84^^,^^85^^,^^86^ However, such applications require extensions to model unobserved intermediate states that arise during differentiation, which are not explicitly represented in the current framework.

CRISPR-Cas9-based lineage-tracing technology is only a decade old, and its application to metastasis is even more recent. Related computational methods remain in their infancy, with considerable room for improvement. Nevertheless, BEAM represents an important step forward by introducing simultaneous Bayesian inference of lineage phylogenies and tissue-migration graphs, as well as Bayesian hypothesis testing, to the field. Because uncertainty about the true structure of the lineage tree and migration graph tends to be high, the Bayesian approach is particularly powerful. We expect the ideas introduced here to encourage continued methods development and to improve our understanding of metastasis.

### Limitations of the study

Although we developed a simulation framework integrating an agent-based model of metastasis with CRISPR-based lineage tracing, it was designed using existing tools to benchmark BEAM across broad-parameter regimes rather than to exhaustively explore all biologically realistic seeding scenarios. In particular, polyclonal seeding was treated in a simplified manner, as we did not systematically vary the number of clones or their relative contributions to individual metastatic sites. While BEAM performed robustly under the diverse conditions examined, developing a more flexible, *de novo* simulation framework remains an important direction for future work.

## Resource availability

### Lead contact

Requests for further information and resources will be fulfilled by the lead contact, Adam Siepel (asiepel@cshl.edu).

### Materials availability

This study did not generate new materials.

### Data and code availability

• The datasets reanalyzed in this study were found in the original reports.^34^^,^^35^^,^^37^^,^^44^ All other data will be shared by the lead contact upon request.
• All code was archived on Zenodo: https://doi.org/10.5281/zenodo.18744445. Up-to-date versions are available on GitHub at https://github.com/CshlSiepelLab/beam, https://github.com/CshlSiepelLab/graphposterior, and https://github.com/CshlSiepelLab/beam_experiments.
• Additional information is available from the lead contact upon request.

## Acknowledgments

This work was performed with assistance from US National Institutes of Health (10.13039/100000002NIH) 10.13039/100000054National Cancer Institute (NCI) grants R01-CA272466 (to D.G.N.) and 5P30CA045508 (to David Tuveson of 10.13039/100013356CSHL), NIH 10.13039/100000057National Institute of General Medical Sciences grant R35-GM127070 (to A.S.), Starr Cancer Consortium grant I16-0060 (to D.G.N.), an 10.13039/100000048American Cancer Society Research Scholar Grant (to D.G.N.), a 10.13039/100020424Weill Cornell Medicine Walter B. Wriston Research Scholar Award (to D.G.N.), 10.13039/100000005Department of Defense 10.13039/100014039Prostate Cancer Research Program Early Investigator Research Award W81XWH-22-1-0068 (to R.N.S.), NIH/NCI Cancer Pharmacology Training Grant CA062948 (to R.N.S.), a 10.13039/100000001National Science Foundation Graduate Research Fellowship (to S.J.S.), a Starr Centennial Scholarship from the 10.13039/100009784Starr Foundation (to S.J.S.), and the 10.13039/100020532Simons Center for Quantitative Biology at CSHL. The content is solely the responsibility of the authors and does not necessarily represent the official views of the NIH. We thank David M. McCandlish, Bruce Stillman, Hannah V. Meyer, and members of the Siepel and Nowak labs for support. We thank Mrinmoy S. Roddur for assistance with MACH2 and Divya Koyyalagunta for assistance with Metient.

## Author contributions

Conceptualization, S.J.S., A. Scheben, D.G.N., and A. Siepel; methodology, S.J.S., A. Scheben, R.H., and A. Siepel; investigation, S.J.S., A. Scheben, L.M.B., R.H., R.N.S., J.X., D.G.N., and A. Siepel; writing – original draft, S.J.S. and A. Siepel; writing – review & editing, S.J.S., A. Scheben, L.M.B., R.H., R.N.S., J.X., D.G.N., and A. Siepel; funding acquisition, S.J.S., R.N.S., D.G.N., and A. Siepel; resources, D.G.N. and A. Siepel; supervision, A. Scheben, D.G.N., and A. Siepel.

## Declaration of interests

The authors declare no competing interests.

## Declaration of generative AI and AI-assisted technologies in the writing process

ChatGPT and Claude were used to assist in software implementation and manuscript preparation. The authors edited the content as needed and take full responsibility for the publication.

## STAR★Methods

### Key resources table

**Table:** 

REAGENT or RESOURCE	SOURCE	IDENTIFIER
**Deposited data**		

Processed data and raw scRNA-seq libraries of metastatic A549 cell lines	Quinn and Jones et al.^34^	Gene Expression Omnibus (GEO): GSE161363
Processed data for KP-Tracer tumors	Yang et al.^35^	Zenodo: https://doi.org/10.5281/zenodo.5847461
Processed data for KPCY pancreatic tumor lineage tracing	Simeonov et al.^44^	Mendeley data: https://doi.org/10.17632/t98pjcd7t6.1
Processed data for EvoCaP prostate cancer lineage tracing	Serio et al.^37^	Available upon request and Code Ocean: codeocean.com/capsule/5050757/tree/v2

**Software and algorithms**		

BEAM	This manuscript	Zenodo: https://doi.org/10.5281/zenodo.18744445 and GitHub: github.com/CshlSiepelLab/beam
graphposterior	This manuscript	Zenodo: https://doi.org/10.5281/zenodo.18744445 and GitHub: github.com/CshlSiepelLab/graphposterior
beam_experiments	This manuscript	Zenodo: https://doi.org/10.5281/zenodo.18744445 and GitHub: github.com/CshlSiepelLab/beam_experiments
Cassiopeia	Jones et al.^14^	GitHub: github.com/YosefLab/Cassiopeia
LAML	Chu and Mai et al.^19^	GitHub: github.com/raphael-group/LAML
MACHINA	El-Kebir et al.^45^	GitHub: github.com/raphael-group/machina
PathFinder	Miura et al.^58^	GitHub: github.com/SayakaMiura/PathFinder
Metient	Koyyalagunta et al.^50^	GitHub: github.com/morrislab/metient
MACH2	Roddur et al.^51^	GitHub: github.com/elkebir-group/MACH2

### Method details

#### Modeling barcode mutation and tissue migration as conditionally independent processes

The barcode-mutation and tissue-migration processes were modeled as conditionally independent continuous-time Markov chains (CTMCs), given the phylogeny and branch lengths. Consider a single branch of the tree with length *b*, leading from a parent node *u* to a child node *v*. We represent the joint conditional probability of a barcode mutation state *B*_*v*_ and tissue label *T*_*v*_ at the child, given a barcode mutation state *B*_*u*_ and tissue label *T*_*u*_ at the parent, as a product of the two conditional probabilities,

$$P\left(T_{v},B_{v}|T_{u},B_{u},b\right)=P\left(T_{v}|T_{u},b\right)\;P\left(B_{v}|B_{u},b\right)\text{.}$$

As a result, the likelihood of observed barcode data **B** and tissue labels **T** at the tips of a tree with topology T and branch lengths **b** can be expressed as a product of phylogenetic likelihoods for the barcode and tissue labels, respectively,

$$L\left(\theta ,T,b;\;B,T\right)=P\left(B,T|\theta_{B},\theta_{T},T,b\right)=P\left(B|\theta_{B},T,b\right)\;P\left(T|\theta_{T},T,b\right)\text{,}$$

where *θ* = {*θ*_**T**_,*θ*_**B**_} such that *θ*_**B**_ contains parameters for the barcode mutation process and *θ*_**T**_ for the tissue migration process. Each CTMC is defined by a corresponding rate matrix and an overall strict clock rate, as detailed below.

The barcode mutation model, adapted from TiDeTree^53^ and similar to LAML,^19^ was designed to describe an irreversible CRISPR-induced mutation process with the potential for silencing at individual sites. In particular, the model is defined by an infinitesimal generator *Q*_*B*_, such that:

$$Q_{B}=\left[\begin{array}{ccccc} -\left(1+l\right) & s_{1} & \ldots & s_{N} & l\\ 0 & -l & \ldots & 0 & l\\ 0 & 0 & \ldots & 0 & l\\ \vdots & \vdots & \vdots & \ddots & \vdots \\ 0 & 0 & \ldots & -l & l\\ 0 & 0 & \ldots & 0 & 0 \end{array}\right]\text{.}$$

The first state represents the unmutated barcode, the last state represents a heritably silenced barcode, and the remaining *N* states represent unique indel (insertion or deletion) outcomes. Notice that all edits and silencing are assumed to be irreversible. Model parameters include the silencing rate *l* and relative indel rates **s**_*N*_, where *s*_*i*_∈**s**_*N*_ is scaled such that $\sum_{i=1}^{N}s_{i}=1$. This scaling ensures that the expected editing rate is one and therefore that estimated branch lengths can be interpreted in units of expected indels per site. We omit the notion of a “scarring window” used in ref.^53^ and allow mutations to occur at any time.

Tissue transitions were modeled with a separate infinitesimal generator *Q*_*T*_, whose state space corresponds to the set of available tissues. Each off-diagonal entry *q*_*ij*_ in *Q*_*T*_ represents the instantaneous migration rate from tissue *i* to tissue *j*. By default, we assume a general time reversible (GTR) parameterization, where each entry is given by *q*_*ij*_ = *r*_*ij*_*π*_*j*_, with symmetric exchangeability rates *r*_*ij*_ = *r*_*ji*_, and equilibrium tissue frequencies *π*_*j*_ such that ∑_*j*_*π*_*j*_ = 1. The full rate matrix takes the form:

$$Q_{T}=\left[\begin{array}{ccccc} -\sum_{j\neq 1}r_{1j}\pi_{j} & r_{12}\pi_{2} & r_{13}\pi_{3} & \ldots & r_{1n}\pi_{n}\\ r_{21}\pi_{1} & -\sum_{j\neq 2}r_{2j}\pi_{j} & r_{23}\pi_{3} & \ldots & r_{2n}\pi_{n}\\ r_{31}\pi_{1} & r_{32}\pi_{2} & -\sum_{j\neq 3}r_{3j}\pi_{j} & \ldots & r_{3n}\pi_{n}\\ \vdots & \vdots & \vdots & \ddots & \vdots \\ r_{n1}\pi_{1} & r_{n2}\pi_{2} & r_{n3}\pi_{3} & \ldots & -\sum_{j\neq n}r_{nj}\pi_{j} \end{array}\right]\text{,}$$

where the diagonal terms ensure that each row sums to zero. The full rate matrix is normalized such that, under the tissue equilibrium frequencies, the expected number of transitions per unit time is equal to one. BEAM explicitly parameterizes the equilibrium frequency of the first tissue, *π*_1_, which is assumed to be the primary source of the tumor, and sets the remaining frequencies to be equal:

$$\pi_{j}=\frac{1-\pi_{1}}{N-1}\;\text{for}\;j\neq 1\text{.}$$

Likelihoods were computed using Felsenstein’s pruning algorithm.^54^ In the case of the barcode mutation model, for a leaf node *i* with observed barcode state *B*_*i*_, the partial likelihood *L*_*i*_(*x*)—indicating the probability of the data beneath node *i* given that node *i* has state *x*—is initialized as:

$$L_{i}\left(x\right)=\left\{ \begin{array}{cc} 1 & \text{if}\;x=B_{i}\\ 0 & \text{otherwise}. \end{array}\right.$$

In the case of the tissue migration model, *L*_*i*_(*x*) is analogously set to 1 if, and only if, *x* is equal to the tissue label at leaf *i*, *T*_*i*_. In both cases, the recurrence relation for internal node *j* with children *k* and *l* is:

$$L_{j}\left(x\right)=\prod_{i\in \left\{k,l\right\}}\left(\sum_{y}L_{i}\left(y\right)P\left(x\to y|b_{i}\right)\right)\text{,}$$

where *P*(*x*→*y*∣*b*_*i*_) represents the conditional probability of state *y* at child node *i*∈{*k*,*l*} given state *x* at parent node *j* and branch length *b*_*i*_. These conditional probabilities are obtained, in the usual way, by computing the matrix exponential *P*_*B*_(*b*_*i*_) = *exp*(*Q*_*B*_*b*_*i*_) or *P*_*T*_(*b*_*i*_) = *exp*(*Q*_*T*_*b*_*i*_).^4^

At the root node *r*, the final likelihood is given by:

$$P\left(B|\theta_{B},T,b\right)=\sum_{x}\pi \left(x\right)L_{r}\left(x\right),\text{or}\;P\left(T|\theta_{T},T,b\right)=\sum_{x}\pi \left(x\right)L_{r}\left(x\right)\text{,}$$

where *π*(*x*) is the equilibrium frequency of root state *x*. Because, in our setting, we typically can assume that the root has the unmutated state, we force *π*(*x*) to be equal to 1 for that state and zero otherwise. For the tissue migration process, we do the same for the primary tissue.

A general implementation of the pruning algorithm was used for the tissue-migration process, but for the barcode mutation process, optimizations were available due to irreversibility of the process. In particular, under this model, many ancestral states are not possible and can be excluded to improve efficiency.^19^ Let *S* be the set of all observed indel states at the tips of the tree, including the unedited state (0) and missing data (−1), and let *S*_*j*_ be the set of observed descendant states for internal node *j*. The allowed ancestral states at node *j*, *A*_*j*_, are:

$$A_{j}=\left\{ \begin{array}{cc} \left\{0\right\} & \text{if}\;0\in S_{j}\;\text{or}\;\left|S_{j}\setminus \left\{-1\right\}\right|>1\\ \left\{0,s\right\} & \text{if}\;S_{j}\setminus \left\{-1\right\}=\left\{s\right\}\;\text{and}\;s\neq 0\\ S & \text{if}\;S_{j}=\left\{-1\right\}. \end{array}\right.$$

The summation in the recursive likelihood calculation can therefore be restricted to *y*∈*A*_*j*_, assuming that *L*_*j*_(*y*) = 0 for *y*∉*A*_*j*_, substantially improving runtime.

With these assumptions, implementation in BEAST 2 was straightforward. For each sampled or proposed tree topology T, branch lengths **b**, barcode mutation parameters *θ*_**B**_, and tissue-migration parameters *θ*_**T**_, we simply calculated the unnormalized posterior density by multiplying the prior densities and the phylogenetic likelihoods of the conditionally independent barcode data and tissue labels,

$$P\left(\theta_{B},\theta_{T},T,b|B,T\right)\;\propto \;P\left(\theta_{B},\theta_{T},T,b\right)\;P\left(B|\theta_{B},T,b\right)\;P\left(T|\theta_{T},T,b\right)\text{.}$$

We relied on the existing functionality in BEAST 2 to explore the space of tree topologies and parameters. Convergence was assessed by checking that the effective sample size for all parameters exceeds 200, and by visually confirming that parameter traces had reached stationarity.

#### BEAM parameters and priors

BEAM includes several free parameters that govern the tree topology, mutation dynamics, and tissue migration process. The phylogenetic tree topology is denoted by T, with associated branch lengths **b**=(*b*_1_,*b*_2_, …,*b*_*n*_). The prior over tree topologies and branch lengths is modeled using a birth-death process, with birth rate *λ* and death rate *μ*, which are treated as free parameters. The initial tree $T_{0}$ can be provided directly in Newick format. In this study, we used LAML to infer a starting tree by approximate maximum likelihood. The barcode mutation model includes a silencing rate parameter *l*, while the relative rates of non-silencing edit outcomes, *s*_1_ … *s*_*N*_, are fixed and normalized based on the relative frequencies in the observed mutation matrix, as similarly done in Chu et al.^19^ and Seidel and Stadler.^53^ A strict molecular clock rate *ν*_*b*_ converts real-time branch lengths, which are directly operated on by MCMC proposals for a tree with fixed height from a specified experiment duration time, into number of substitutions before applying the rate matrix exponential to compute transition probabilities for the branch. Tissue migration dynamics are modeled with the relative migration rates between tissues denoted *r*_*ij*_, and a strict clock rate for tissue migration is represented by *ν*_*t*_. The equilibrium frequency of the primary tissue is denoted *π*_1_, with the frequencies of the remaining tissue types set to a uniform value to ensure that all frequencies sum to one.

The prior distributions assigned to these parameters were as follows:

λ∼Uniform(0,1),μ∼Uniform(0,1),

$$l∼\text{Exponential}\left(\beta_{s}\right),\text{where}\;\beta_{s}=0.1\text{,}$$

$$\nu_{b}∼\text{Exponential}\left(\beta_{v}\right),\text{where}\;\beta_{v}=0.1\text{,}$$

$$r_{ij}∼\text{Exponential}\left(\beta_{q}\right),\text{where}\;\beta_{q}=1.0\text{,}$$

$$\nu_{t}∼\text{Exponential}\left(\beta_{t}\right),\text{where}\;\beta_{t}=0.1\text{,}$$

$$\pi_{1}∼\text{Uniform}\left(0,1\right)\text{.}$$

Initial values for parameters were selected based on empirical convergence behavior observed in pilot runs. We used minimally informative priors in this study, but BEAST 2 allows these prior distributions to be adjusted easily, as needed.

#### Simulation model for cancer evolution and CRISPR lineage tracing

We developed an agent-based model of cancer cell dynamics built on three tools: the Tool for Tumor Progression,^55^ the MACHINA simulator,^45^ and the Cassiopeia barcode simulator.^14^ MACHINA extended the original tumor progression model to include metastasis. Below, we briefly describe the key modeling components.

Cell birth and death follow a multiplicative fitness landscape with logistic constraints. The birth rate of cell *c* is

$$b\left(c\right)=\min\left(1,\max\left(0,0.5\times {\left(1+0.1\left(1-\frac{N\left(\sigma_{c}\right)}{50,000\times \left|\sigma_{c}\right|}\right)\right)}^{\left|\sigma_{c}\right|+1}\right)\right)\text{,}$$

where *N*(*σ*_*c*_) is the number of cells in the same tissue with driver mutation set *σ*_*c*_. A cell divides if *r*∼Uniform(0,1)<*b*(*c*); otherwise, it dies. Upon division, one daughter retains the parent’s genotype, while the other mutates with probability *p* = 0.1. Mutations are drivers with probability *p*_*d*_=(2 × 10^−7^)(|*σ*_*c*_|+1), and passengers otherwise. Each generation, birth/death decisions are made for all cells, followed by metastasis decisions for each existing tissue. The probability of migration from tissue *t* is

$$P_{\text{migration}}\left(t\right)=\mu_{t}\prod_{y\in Y\left(t\right)}\left(N\left(t,y\right)\times \left|y\right|\right)\text{,}$$

where *N*(*t*,*y*) is the number of cells in tissue *t* with driver mutation set *y*, and *μ*_*t*_ is the migration rate per cell per driver. Migration occurs from a tissue if *r*∼Uniform(0,1)<*P*_migration_(*t*) and the number of migrating cells is then drawn from Poisson(1) and migrated to a destination tissue chosen from a uniform transition matrix across ten tissues.

Simulations run for 250 generations, after which cells are downsampled. The lineage tree with branch lengths in cell divisions is then used to simulate CRISPR barcodes at the tips using Cassiopeia, assuming a uniform mutation rate across sites, default heritable silencing rate of 0.0001, and default stochastic silencing rate of 0.01. The output includes a cells-by-barcode matrix, tissue labels, and ground-truth phylogeny and migration history.

#### Simulated data

To generate simulated datasets under ideal conditions, we ran 100 simulations with a mutation rate of 0.0025 mutations per barcode site per cell division and a migration rate of 1 × 10^−6^ per cell. These simulations produced a ∼50% saturated character matrix and a migration graph of intermediate complexity (see representative examples in Figure S4). To simulate data with variable complexity of barcode mutation and tissue migration, we simulated 20 datasets for each combination of barcode mutation rates {0.0005,0.001,0.0025,0.005,0.01} per barcode site per cell division and tissue migration rates {1 × 10^−7^,1 × 10^−6^,1 × 10^−5^,1 × 10^−4^} per cell (representatives shown in Figure 2B). Additional data simulation conditions are described within figure captions alongside respective results. For all conditions, we simulated trees with 50 cells after downsampling and used a traversal of the downsampled tissue-labeled tree to obtain the ground-truth migration graph. Simulations in which no migrations occurred were discarded and replaced with new ones until the desired number was reached. We note that for the results shown in Figures 2A and 2B, all simulations were processed with all migration history inference methods with the exception of seven simulations for PathFinder in Figure 2A, and three simulations for MACHINA and 69 simulations for PathFinder in Figure 2B due to long runtimes.

#### Alternative methods for benchmarking

For consistency and efficiency in benchmarking, we developed a pipeline to process the same simulated datasets by several methods in parallel. Because all methods except BEAM required a fixed lineage phylogeny as input, we selected a single phylogeny inference method for preprocessing and, for each simulated mutation matrix, we applied this method once and used the resulting tree for all downstream steps, except when a different phylogeny inference method was indicated in Figure 2A. Based on published benchmarks and our preference for a probabilistic reconstruction method, we chose the LAML method^19^ for phylogeny inference. Notably, LAML uses an approximate maximum-likelihood method to estimate both a topology and branch lengths under a similar mutation model to the one assumed by BEAM. For initialization of LAML, we used the Cassiopeia-Greedy algorithm^14^ to generate an approximate tree topology without branch lengths from the mutation matrix.

Once this cell-lineage tree was obtained, we applied the following methods for migration-graph inference (listed in order of increasing sophistication).

##### Random

Tissue labels at the tips of the input phylogeny were used to randomly assign tissue states to internal nodes, ignoring the tree structure. This approach served as a baseline for migration-graph accuracy under random ancestral state assignments and provided an approximate lower bound for the performance of other methods.

##### Consensus

For each internal node, tissue labels for all tips in its sub-tree were collected and the most common tissue label among those tips was assigned to the node. This was the simplest possible use of the tree structure for tissue labeling.

##### Parsimony

We applied our own implementation of the Fitch-Hartigan algorithm^56^^,^^57^ for the “small parsimony problem” of discrete ancestral state reconstruction. This method minimized the number of migration events on an input cell lineage tree, without refining that tree to resolve polytomies or considering other parsimony criteria.

##### MACHINA

We applied MACHINA^45^ v1.0 in Parsimonious Migration History with Tree Refinement (PMH-TR) mode to both refine a given clone tree into a binary tree, resolving polytomies, and label its internal nodes with tissue assignments to infer a migration history. The MACHINA objective is to minimize, in order: the number of migration events (directed edges in the migration graph), the number of co-migrations (unique edges in the migration graph, so that a single directed edge and a directed multi-edge each only contribute a count of one), and the number of seeding sites (distinct tissues serving as sources of migration) using mixed integer linear programming under a parsimony criterion. We used MACHINA in unrestricted migration mode to allow the migration graph to take any topology rather than being restricted to predefined patterns. MACHINA returned a single most parsimonious labeling.

##### PathFinder

We used PathFinder^49^ v2.0 to infer migration histories from sequence alignments with known tissue labels. PathFinder first builds a Neighbor-Joining tree from the alignment, then applies a probabilistic algorithm to infer migration events and resolve polytomies by permutation of the tree. To generate the input sequence alignment, we converted the indel matrix containing multiple mutations per site into a binary format, representing each mutation as a separate site with “A” for unmutated and “T” for mutated states. We ran PathFinder with default settings, enabling the relax_threshold flag to retain low-probability histories that proved necessary to prevent run failures. Although it was originally described as a Bayesian approach, we received clarification from the PathFinder authors that the method was intended to output a single best migration history rather than a properly calibrated posterior distribution of migration histories, so we computed performance metrics on the single best solution.

##### Metient

We applied Metient^50^ v0.1.3.4.13 in a similar way to MACHINA. Metient minimizes the same three criteria of migrations, co-migrations, and seeding sites while resolving polytomies in the tree, similar to MACHINA, but it allows flexibility in how these criteria are weighted. We ran Metient-evaluate using the default parsimony weights in pancancer_genetic_uniform_weighting() (0.5448 for migrations, 0.2727 for co-migrations, and 0.1825 for seeding sites) that were recommended by the authors for non-human data. We also tested Metient-calibrate on our simulated data, which found different optimal weights but led to worse performance, so we report results using the default weights and Metient-evaluate. Tissue locations for tips were encoded in the input metadata as present and nodes were marked absent from all tissue locations. We used the default settings for Metient-evaluate except that we applied solve_polytomies = True. For precision and recall based performance metrics, we integrated across all output solutions to obtain an edgewise probability graph. Specifically, to get probabilities for all output solutions, the loss scores were min-max normalized before applying temperature-scaled softmax with a fixed temperature of 0.5, as recommended by the authors.

##### MACH2

Finally, we applied MACH2^51^ v1.0.1, an extension of MACHINA which solves the same PMH-TR problem but enumerates the full set of equally parsimonious migration histories rather than returning a single solution. All solutions are derived from the same integer linear programming formulation used in MACHINA, but MACH2 leverages combinatorial properties of the solution space to exhaustively enumerate all optimal solutions. We ran MACH2 in the default mode and the output solutions were all weighted equally (as in^51^) to construct an edgewise probability migration graph summarizing the full solution set.

#### Precision, recall, and F1 score calculations

To evaluate migration graph inference, we assessed the precision and recall of inferred edges in terms of true positive (TP), false positive (FP), and false negative (FN) edges in the graph. Importantly, both the simulated and inferred migration graphs are actually *directed multigraphs*, with the possibility of multiple edges between each pair of nodes. Therefore, motivated by ref.,^51^ we computed precision and recall using *counts* of each type of edge per graph rather than by considering their presence/absence. This strategy penalizes cases where the types of migration events are correctly inferred but the numbers of events are not—a kind of error that is particularly relevant when evaluating the clonality of seeding.

Given a true migration graph *G* and an inferred migration graph *G*^∗^ let *E*_*ij*_ and $E_{ij}^{*}$ represent the numbers of directed edges in *G* and *G*^∗^, respectively, that run from tissue *i* to *j* where *i*≠*j*. The counts TP, FP, and FN were derived from these edge counts as follows:

$$\text{TP}=\sum_{i\neq j}\min\left(E_{ij},E_{ij}^{*}\right),\text{FP}=\sum_{i\neq j}\max\left(E_{ij}^{*}-E_{ij},0\right),\text{FN}=\sum_{i\neq j}\max\left(E_{ij}-E_{ij}^{*},0\right)\text{.}$$

The precision (Pr), recall (Re), and F1 score were then calculated by the standard formulas:

$$\mathrm{Pr}=\frac{\text{TP}}{\text{TP}+\text{FP}},\text{Re}=\frac{\text{TP}}{\text{TP}+\text{FN}},F1=2\times \frac{\mathrm{Pr}\times \text{Re}}{\mathrm{Pr}+\text{Re}}\text{.}$$

For precision-recall curves from MCMC samples, we first converted each sample *s* of a tissue-labeled tree into a migration multigraph, with an integer count $E_{ij}^{\left(s\right)}$ for each type of edge (*i*,*j*). We then let *f*_*ij*_(*c*) be the fraction of such sampled multigraphs that have *c* or more counts of edge (*i*,*j*), that is, the fraction with $E_{ij}^{\left(s\right)}\geq c$. Because it is derived from samples from the approximate posterior distribution, this fraction *f*_*ij*_(*c*) can be interpreted as an estimate of the posterior probability that the inferred edge count $E_{ij}^{*}\geq c$. We therefore subjected this value to a varying posterior-probability threshold, *p*, such that 0 ≤ *p* ≤ 1. For each choice of *p*, we estimated $E_{ij}^{*}$ as the maximum number of edges *c* such that *f*_*ij*_(*c*)≥*p*. We then evaluated the counts TP, FP, and FN using that value of $E_{ij}^{*}$. For the F1 score calculations, we fixed the threshold *p* at 0.5.

We used the same strategy for MACH2, treating each reported solution as if it were a draw from a posterior distribution (so that they were equally weighted). For Metient, we obtained the posterior distribution over solutions through min-max normalization and temperature-scaled softmax, as recommended by the authors, and then weighed solutions by their posterior probability when computing an edgewise probability graph to then apply thresholds to.

We followed ref.^87^ in using precision-recall curves rather than receiver operating characteristic (ROC) curves, which are problematic in the multigraph setting.

#### Calculating excess migrations relative to parsimony

To calculate the excess migrations predicted by BEAM, we began with a sample from the joint posterior distribution over tissue-labeled trees and model parameters given the data. For each sampled tree, the number of BEAM-inferred migration events was obtained in the usual way, by counting branches in the tree for which the parent and child tissue labels disagreed. The tissue labels were then removed from the internal nodes of the tree and our implementation of the Fitch-Hartigan parsimony algorithm was applied to obtain an estimate of the minimum possible number of migration events given the leaf labels only. This minimum possible count was subtracted from the BEAM estimate to obtain an estimate of the excess migrations per sample. Finally, an estimate of the posterior expected excess migration count was obtained by averaging these per-sample excess counts across all samples.

#### Application to lung, prostate, and pancreatic cancer datasets

As detailed in ref.,^34^ the lung cancer data was derived from orthotopically xenografted KRAS-mutant A549 lung adenocarcinoma cells, engineered with CRISPR barcodes and surgically implanted into the left lungs of four mice. As in the published analysis, we focused on data for one mouse (labeled “5k”) with 100 clonal populations (CPs), which were tracked over 54 days. We began with the provided allele table and metadata for mouse 5k. We excluded the 17 CPs excluded in the original paper (5, 16, 18, 25, 33, 38, 39, 41, 50, 53, 65, 69, 75, 81, 87, 88, 93), 9 CPs with only one observed tissue label (22, 29, 46, 48, 49, 78, 85, 94, 96), and 21 large CPs (1–4, 6–15, 17, 19–23, 27, 31) that were computationally prohibitive to analyze, and analyzed the remaining 53 CPs with BEAM. To infer tissue migration graphs, we used the coarse-grained tissue annotations, specifically the metadata labels LL, M, RL, and Liv, which were mapped to each cell barcode.

The prostate cancer data was obtained from a somatically engineered mouse with knockout of PTEN/P53^88^ and the introduction of CRISPR barcodes. Cancer developed in 10 mice over an average of ∼380 days, producing a variable number of CPs per mouse. The prostate cancer datasets for all mice were re-processed using the EvoTraceR pipeline (v1.0.4), following the authors’ original analysis protocol, resulting in 522 CPs. We excluded 2 CPs (MMUS1875 CP1 and MMUS1466 CP1) that were computationally prohibitive to analyze and we excluded 99 CPs with only one observed tissue label. This left us with 421 CPs to analyze across the 10 mice with BEAM. With MACH2 analyses, we also had to exclude 3 more CPs (MMUS1469 CP1 and 3; MMUS1495 CP1) due to long runtimes.

For both datasets, these preprocessing steps produced mutation matrices and associated tissue labels for each CP. We then inferred a baseline tree for each CP using the Cassiopeia-Greedy algorithm^14^ and supplied this tree to LAML^19^ for tree and branch-length estimation by maximum likelihood. These LAML-estimated lineage trees were later used both as the starting trees for BEAM and as the fixed input trees for all other migration-graph inference methods.

We incorporated two additional reference datasets for a supporting comparison of CRISPR barcode mutational content in metastatic CPs. From a published pancreatic cancer dataset^44^ with 83 total clones from two mice, we retained 20 metastatic clones (clones 2, 4, 5, 9, 10, 11, and 22 from mouse M1 and clones 1, 2, 4, 5, 6, 9, 15, 19, 21, 23, 25, 29, 63 from mouse M2) after filtering out 57 clones with only one tissue label, one clone that was too large to analyze with BEAM, and 27 clones with no phylogenetically informative mutations (22 of which overlap with those clones having only one tissue label). We then used the provided mutation matrices directly with minor preprocessing to convert them into the necessary format for our analyses. From an additional lung cancer dataset,^35^ we retained five metastatic clones (3457_Apc_T1, 3457_Apc_T4, 3508_Apc_T2, 3513_NT_T1, and 3519_Lkb_T1 all formatted as mouse_genotype_clone) from the nine clones available in the deposited data after filtering out four CPs too large to analyze with BEAM. We derived mutation matrices from the provided overall allele table in a similar manner to our pre-processing of the main lung cancer dataset,^34^ since both datasets originated from the same laboratory and reported similar pre-processing steps within Cassiopeia,^14^ and we again used the coarse-grained tissue annotations here that merge multiple samples from the same tissue. All preprocessing scripts were made publicly available, see Data and code availability.

#### Previously reported levels of M2M and PR for real data

For the lung cancer dataset, we used the numbers of CPs reported to exhibit metastasis-to-metastasis (M2M) seeding and primary reseeding (PR) under the labels “metastatic cascade” and “reseeding” in Figure 7 of ref.^34^ across all CPs that they analyzed. For the prostate cancer dataset, however, the published analysis reported fractions of edges in full lineage trees within or across CPs,^37^ which did not directly correspond to our metrics. Therefore, we obtained the MACHINA migration-graph output files from the authors and recalculated the percentage of CPs showing evidence of M2M or PR events across all CPs in their study. Specifically, a CP was labeled as M2M if it had any edge excluding the primary tissue, and as PR if it had an edge with a non-primary source tissue and the primary tissue as the target tissue.

#### Hypothesis testing with Bayes factors

Bayes factors are a flexible and powerful means for model comparison and hypothesis testing in a Bayesian setting, but they require evaluation of the marginal likelihood of the data, integrating over parameters and any other latent variables. In our case, the marginal likelihood given the model *M* has the formidable form:

$$P\left(B,T|M\right)=\sum_{T}∭P\left(B|\theta_{B},b,T\right)P\left(T|\theta_{T},b,T\right)P\left(\theta_{B},\theta_{T},b,T\right)\;d\theta_{B}\;d\theta_{T}\;db\text{.}$$

This expression—a version of what is sometimes called the “Felsenstein equation”^89^^,^^90^—is both a sum over all possible tree topologies and an integral over all possible combinations of branch lengths and other parameters, making it highly intractable.

Nested sampling, however, offers an effective approach for approximating this integral by MCMC, and a convenient package for nested sampling is already available for BEAST 2.^91^ This approach works by mapping the parameter space to a prior mass using a cumulative density function,^92^ thereby transforming the high-dimensional integral into a one-dimensional integral over prior mass. The integral is approximated by sequentially sampling parameter sets of increasing likelihood and computing a weighted sum of these likelihoods, where the weights correspond to the changes in prior mass between samples.

Once marginal likelihoods are available for two models *M*_1_ and *M*_2_, a Bayes factor can easily be computed as a ratio of their marginal likelihoods. In the log space used by BEAST 2,

$$\ln\left(\text{Bayes}\;\text{factor}\right)=\ln\left[\frac{P\left(B,T|M_{1}\right)}{P\left(B,T|M_{2}\right)}\right]=\ln\left[P\left(B,T|M_{1}\right)\right]-\ln\left[P\left(B,T|M_{2}\right)\right]\text{.}$$

We simply interpreted values of *ln*(Bayes factor)<-1.1 as indicating positive support for *M*_2_, values of *ln*(Bayes factor) > 1.1 as indicating positive support for *M*_1_ and values between −1.1 and 1.1 (inclusive) as not positively supporting either model (see^93^).

In our case, we began by running nested sampling with 10,000 MCMC iterations per sub-chain and a single active particle. We then increased the number of active particles as much as computationally feasible and following the recommendations of the "Taming the BEAST" tutorial.^94^

#### Calculating mutual information of tissue transitions

For a simple, model-free evaluation of the information in the data relevant to migration history in the posterior distribution, we computed the mutual information between the sampled tissue assignments at parent and child nodes across all branches of the tree. First, a collection of trees sampled by BEAM was traversed to fill an *n*×*n* matrix of counts *C*, where *n* is the number of distinct tissue labels, and *C*_*ij*_ represented the total number of inferred transitions from tissue *i* to tissue *j* across branches. The count matrix was then normalized to obtain a matrix of joint probabilities of pairs of tissues, $P_{ij}=\frac{C_{ij}}{\sum_{i,j}C_{ij}}$, as well as vectors of marginal probabilities, *P*_*i*._ = ∑_*j*_*P*_*ij*_ and *P*_.*j*_ = ∑_*i*_*P*_*ij*_. The mutual information between the source tissue *X* and target tissue *Y* was then calculated as:

$$I\left(X;Y\right)=\sum_{i=1}^{n}\sum_{j=1}^{n}P_{ij}\;\log_{2}\left(\frac{P_{ij}}{P_{i.}P_{.j}}\right)\text{.}$$

To normalize for differences in entropy between datasets, the mutual information was scaled by the average entropy of the marginals:

$$I^{\prime }\left(X;Y\right)=\frac{2\times I\left(X;Y\right)}{H\left(X\right)+H\left(Y\right)}\text{,}$$

where *H*(*X*) = -∑_*i*_*P*_*i*._*log*_2_(*P*_*i*._) and *H*(*Y*) = -∑_*j*_*P*_.*j*_*log*_2_(*P*_.*j*_).

Since BEAM models tissue transitions as a Markov process, where ancestral tissue labels are more likely to persist within the same tissue along the cell lineage tree rather than to result from frequent migration between tissues, we expected a well-fit model to produce a high normalized mutual information score.

#### Evaluating the PR test on simulated data

All ground-truth PR simulations from the variable rates simulated dataset in Figure 2B were collected, excluding any with a mutation rate of 0.0005 or a migration rate of 1 × 10^−4^ due to poor performance in prior evaluations or a migration rate of 1 × 10^−7^ due to a lack of ground truth migration graphs with PR. This resulted in 39 valid PR simulations. All non-PR ground truth simulations were then gathered, excluding those with migration rate of 1 × 10^−4^, migration rate of 1 × 10^−7^, or mutation rate of 0.0005 and 39 of them were randomly sampled to match the number of PR simulations for class balance. We then applied the PR hypothesis testing procedure to all 78 simulations. Classification was based on the Bayes factor, where simulations with *ln*(Bayes factor) > 1.1 were classified as PR and *ln*(Bayes factor)<-1.1 as non-PR. This classification scheme ignores simulations classified as not supporting either model with −1.1≤*ln*(Bayes factor)≤1.1, so we provide results for all three outcome categories.

### Quantification and statistical analysis

Quantitative and statistical analyses are described in the relevant Method details sections and figure legends. All analyses were performed in Python.

## Supplemental information

Document S1. Figures S1–S18 and Tables S1 and S2

Document S2. Transparent peer review records for Staklinski et al.

Document S3. Article plus supplemental information
