# Supplementary material for: Bayesian inference of tissue-migration histories in metastatic cancer from cell-lineage tracing data
Source: Cell Genom. 2026 Mar 30;6(6):101193. doi: 10.1016/j.xgen.2026.101193 (PMC13261679; doi:10.1016/j.xgen.2026.101193)
Supplement: Document S1. Figures S1–S18 and Tables S1 and S2 [file mmc1.pdf]

Cell Genomics, Volume *6*

Supplemental information

Bayesian inference of tissue-migration histories in metastatic cancer from cell-lineage tracing data

Stephen J. Staklinski, Armin Scheben, Lise M. Brault, Rebecca Hassett, Ryan N. Serio, Jiawei Xing, Dawid G. Nowak, and Adam Siepel

SUPPLEMENTARY FIGURES

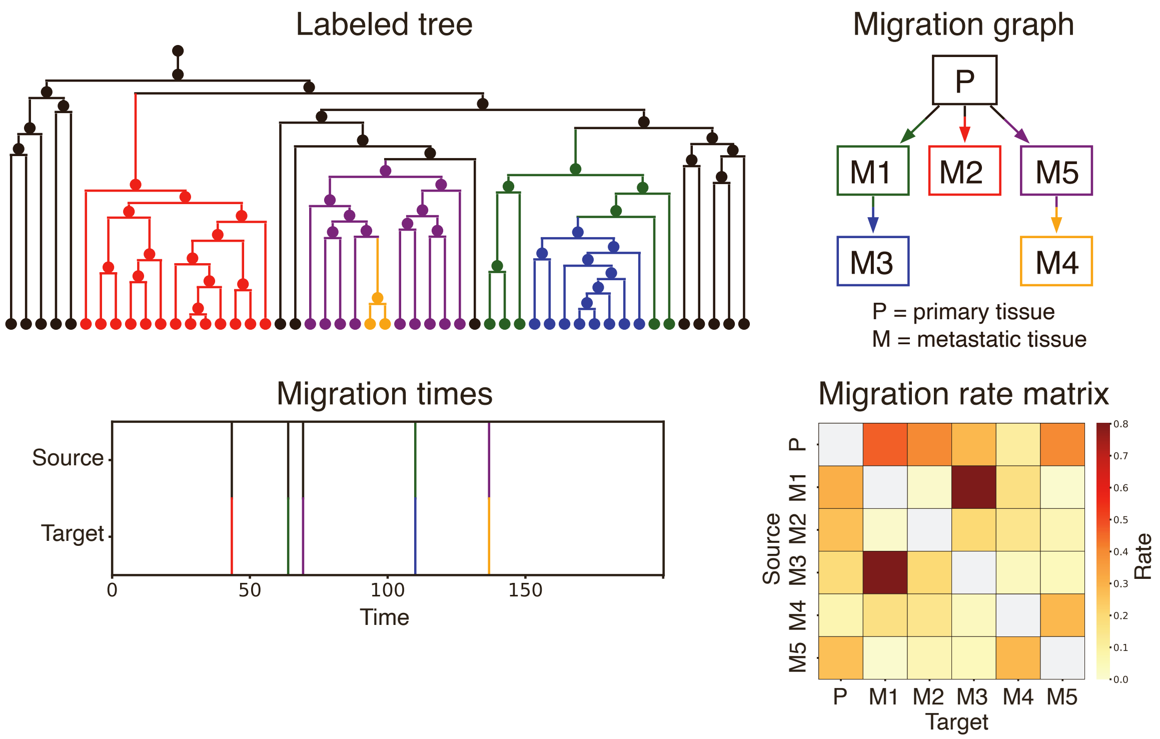

Figure S1. Example posterior samples from joint inference of lineage trees, migration graphs, and model parameters, Related to Figure 1
An example of a sample from BEAM's posterior distribution, including a labeled tree (top left), the corresponding migration graph (top right) and derived migration times (bottom left). The migration rate parameters (bottom right) are also sampled from the posterior distribution.

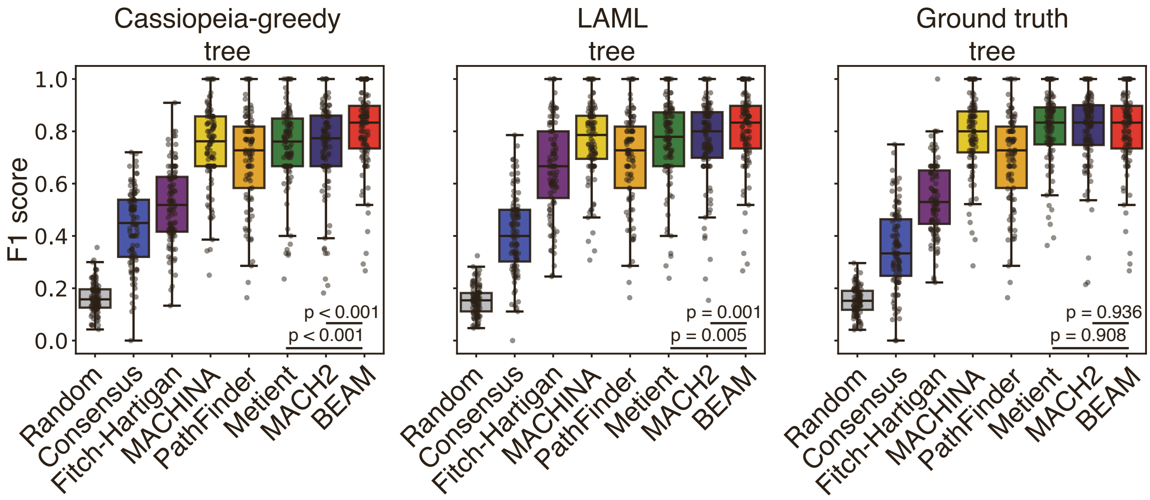

Figure S2. F1 score comparison for migration-graph inference methods run on simulated data, Related to Figure 2

F1 scores for the same experiment as in **Figure 2A**, based on a threshold of 0.5 posterior probability for Metient, MACH2, and BEAM. Reported p-values are from paired t-tests only between MACH2 and Metient compared to BEAM. Again, inference results are shown for each method, conditioned on the input tree topology inference method indicated above each plot. Because PathFinder and BEAM do not rely on a fixed input tree, the same results are displayed for these two methods across all plots.

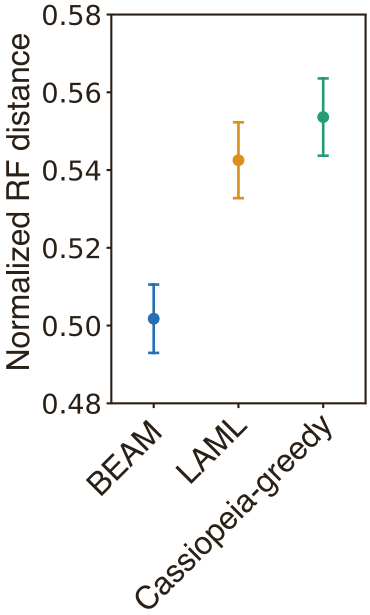

Figure S3. Tree reconstruction accuracy across inference methods measured by Robinson–Foulds distance, Related to Figure 2
Mean normalized Robinson–Foulds (RF) distance measuring tree reconstruction accuracy for the simulations shown in **Figure 2A**, comparing Cassiopeia-greedy, LAML, and BEAM inference results. The normalized RF distance is calculated as the symmetric difference between the sets of bipartitions in the true and inferred trees, divided by the total number of bipartitions across both trees. Trivial bipartitions (those separating a single leaf from all others) are excluded from the calculation. The expectation over the distances for all posterior distribution samples was calculated for BEAM. Points represent the mean $\pm$ standard error of the mean across replicate simulations.

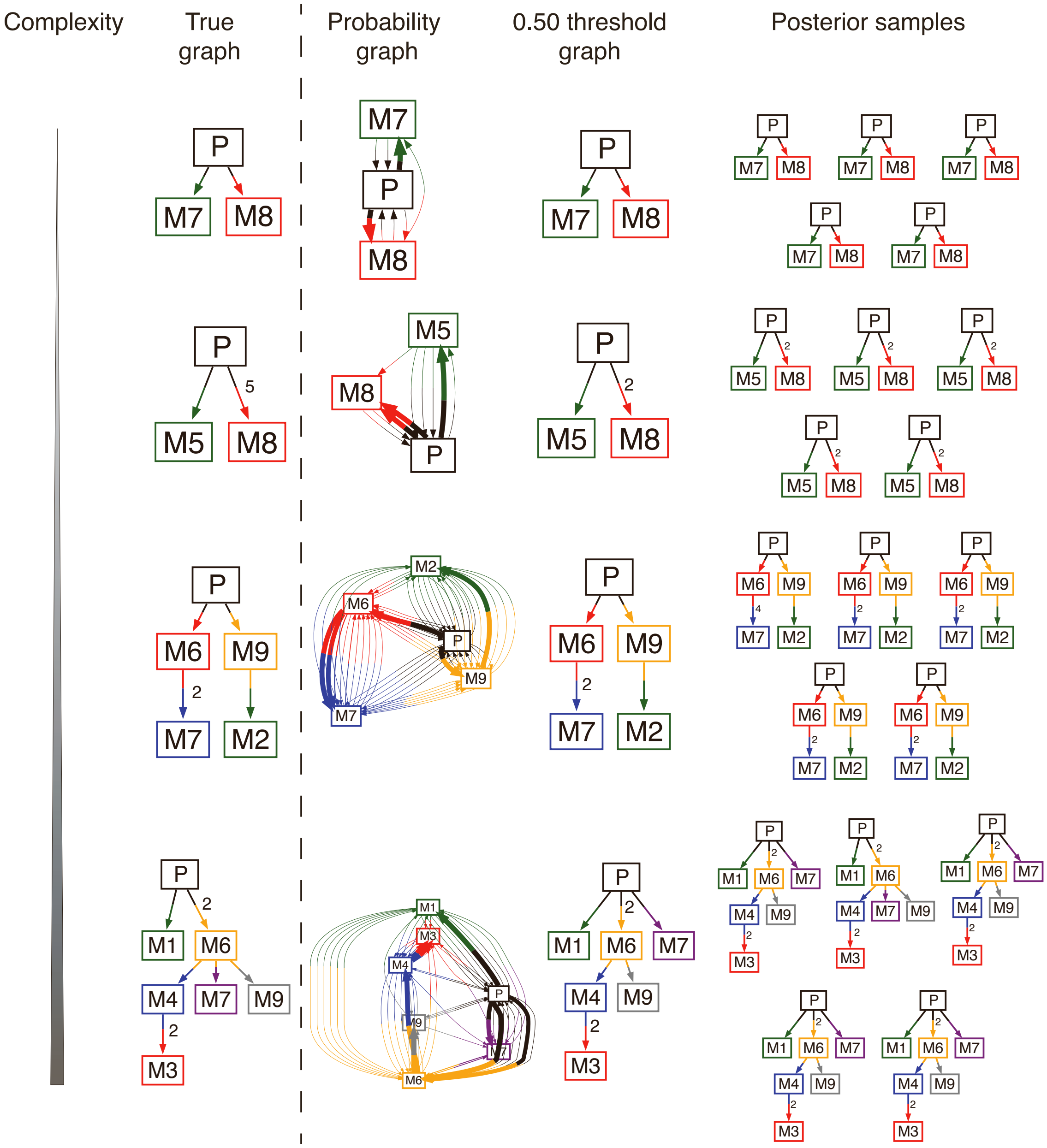

Figure S4. Representative posterior migration graphs for simulated datasets of increasing complexity, Related to Figure 2
Representative graphs sampled from BEAM's posterior distribution for simulated datasets of increasing complexity in the favorable parameter regime shown in **Figure 2A**. Shown for each case are the true migration graph, the edgewise probability-weighted graph, the 0.5-threshold graph, and individual samples from the posterior distribution. Numbers next to edges indicate multi-edge counts. Edges without a number represent single migration events. The P label represents the primary tissue and labels beginning with M are metastatic tissues in the simulations.

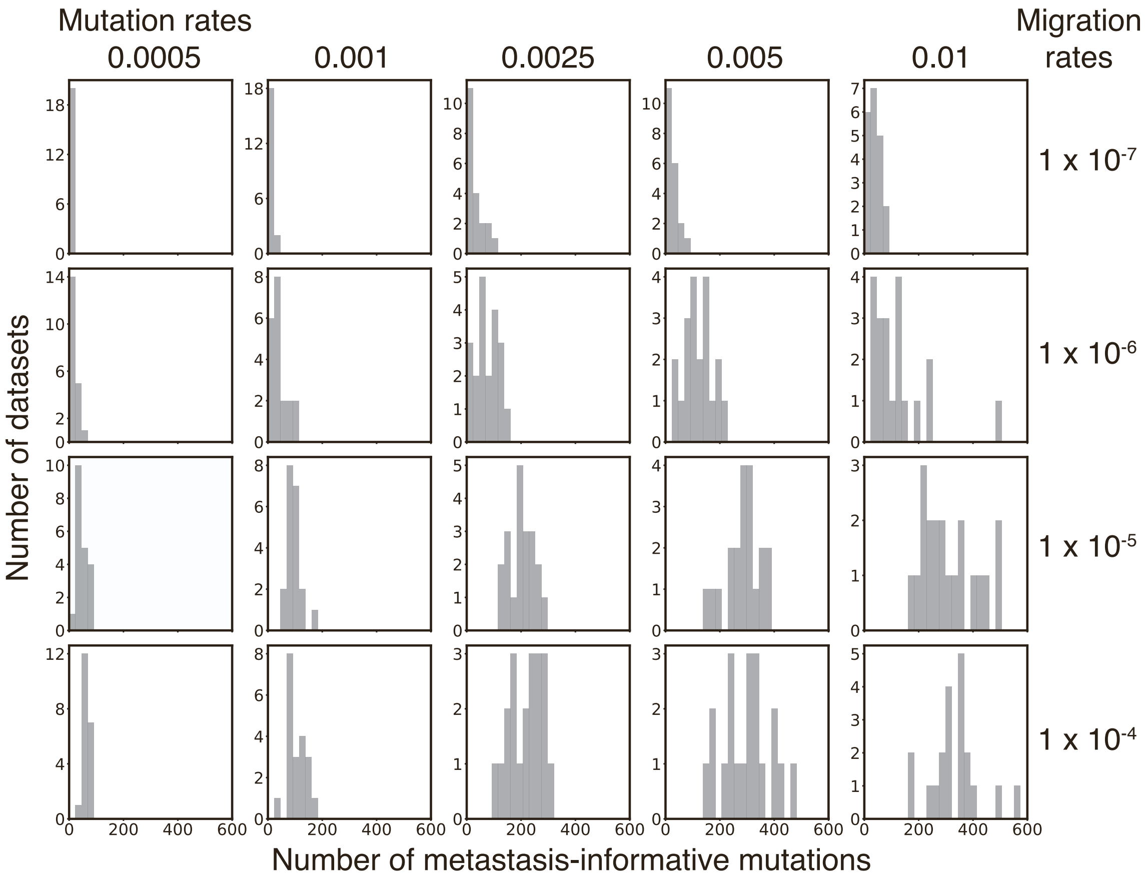

Figure S5. Number of metastasis-informative mutations across simulated mutation and migration regimes, Related to Figure 2

Number of metastasis-informative mutations per simulation for the variable parameter simulations shown in **Figure 2B**. A metastasis-informative mutation was defined as a mutation occurring on a migration branch in the ground-truth tree, where the parent and child tissues differ. Migration branches were identified directly from the simulation output. Mutations were mapped to branches by inferring the accumulated indel states at each internal node using a post-order traversal that intersected child node states, followed by a pre-order pruning step to retain only new mutations arising along each branch. The total number of metastasis-informative mutations was then calculated as the sum of new mutations occurring on all migration branches.

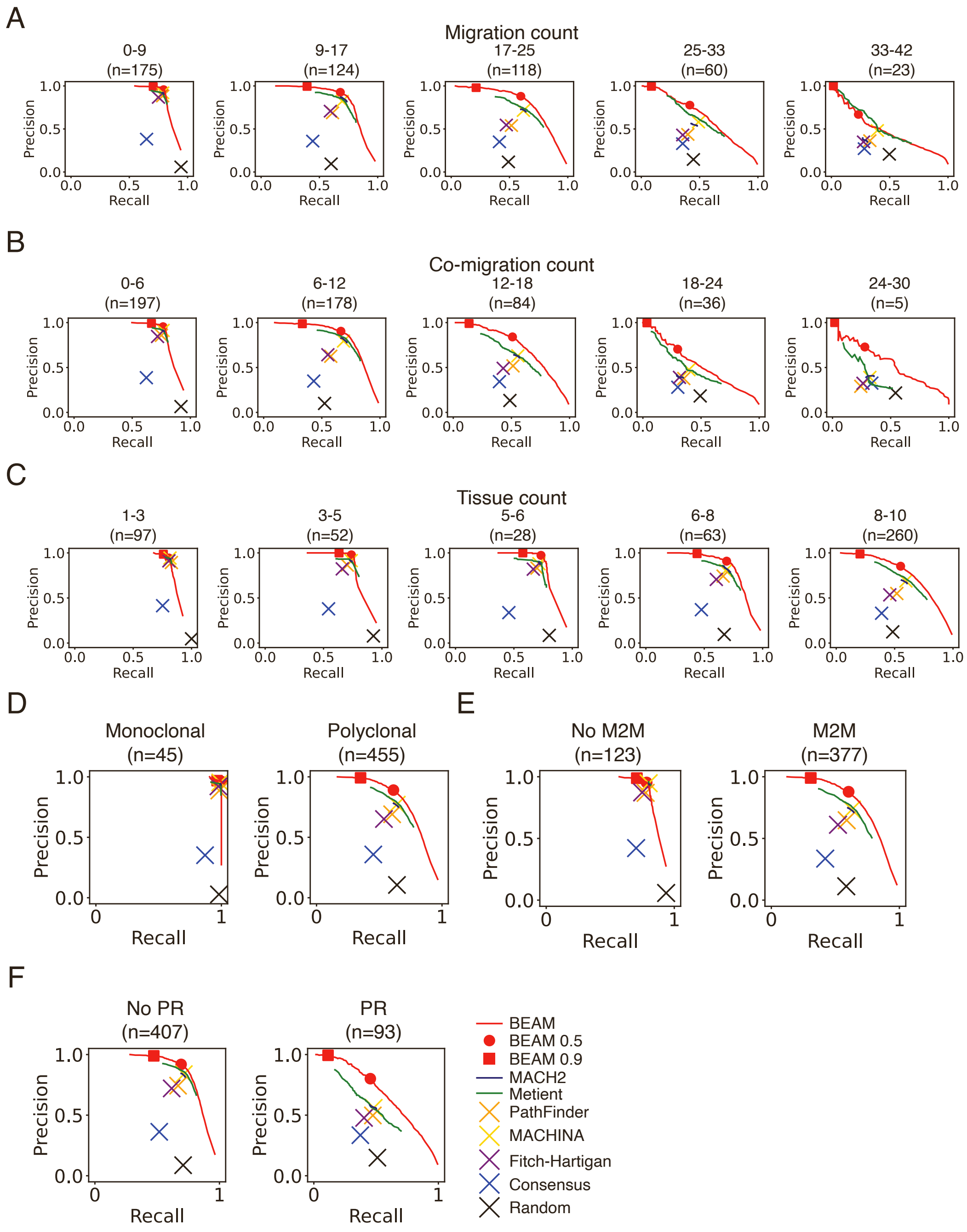

Figure S6. Precision–recall performance stratified by simulated migration-graph properties, Related to Figure 2

Precision-recall curves for 500 total simulated datasets from the combination of **Figure 2A–B** stratified by: (A) number of migration events (directed edges in the graph), (B) number of co-migration events (unique edges in the graph, so that a single directed edge and a directed multi-edge each only contribute a count of one as in[S4?,S5]), (C) number of tissues, (D) clonality (whether any multi-edges exist or not in the graph for polyclonal and monoclonal respectively), (E) presence of metastasis-to-metastasis (M2M) seeding, and (F) presence of primary reseeding (PR). For plots where groups are defined by ranges, each range excludes its left endpoint and includes its right endpoint. For methods that require an input phylogeny, the results are shown using the tree inferred by LAML.

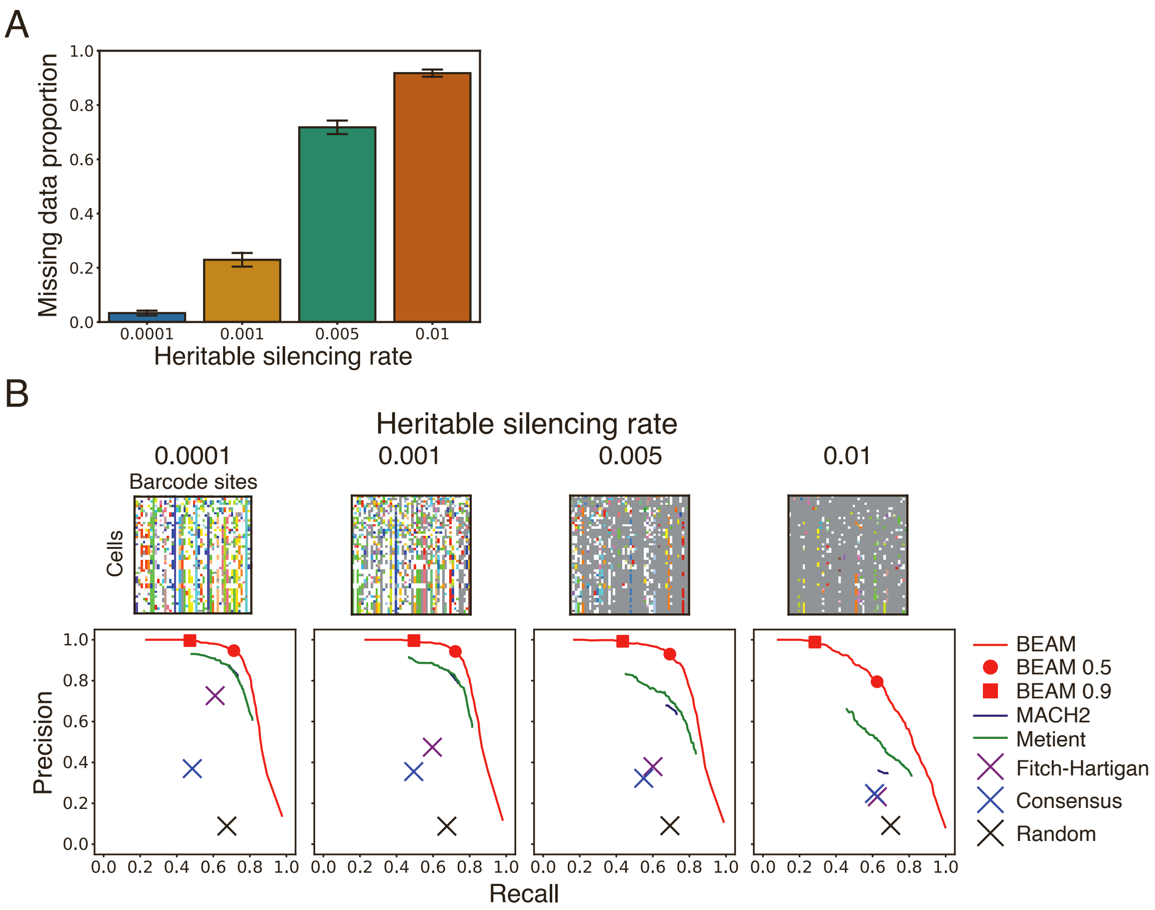

Figure S7. Effects of CRISPR barcode missing data on migration-graph inference accuracy, Related to Figure 2

(A) Proportion of missing mutation matrix entries across simulated datasets for each heritable silencing rate. Bars summarize 100 simulations generated using the same ground truth migration histories as in **Figure 2A**, with new barcode data overlaid using the indicated heritable silencing rates. Bars are shown as mean $\pm$ standard deviation (B) Representative barcode matrices (top), with gray color indicating missing data entries, and precision–recall curves (bottom) for BEAM, MACH2, Metient, Fitch–Hartigan parsimony, Consensus, and a Random baseline across the same heritable silencing-rate range. BEAM point estimates at posterior probability thresholds 0.5 and 0.9 are shown. Methods requiring an input phylogeny use the LAML-inferred tree. Results for the 0.0001 heritable silencing rate come directly from **Figure 2A**. MACHINA and PathFinder are excluded in the 0.0001 silencing rate plot for consistency across other silencing-rate groups, as they were not run for all settings due to their generally lower performance and longer runtimes relative to MACH2 and Metient.

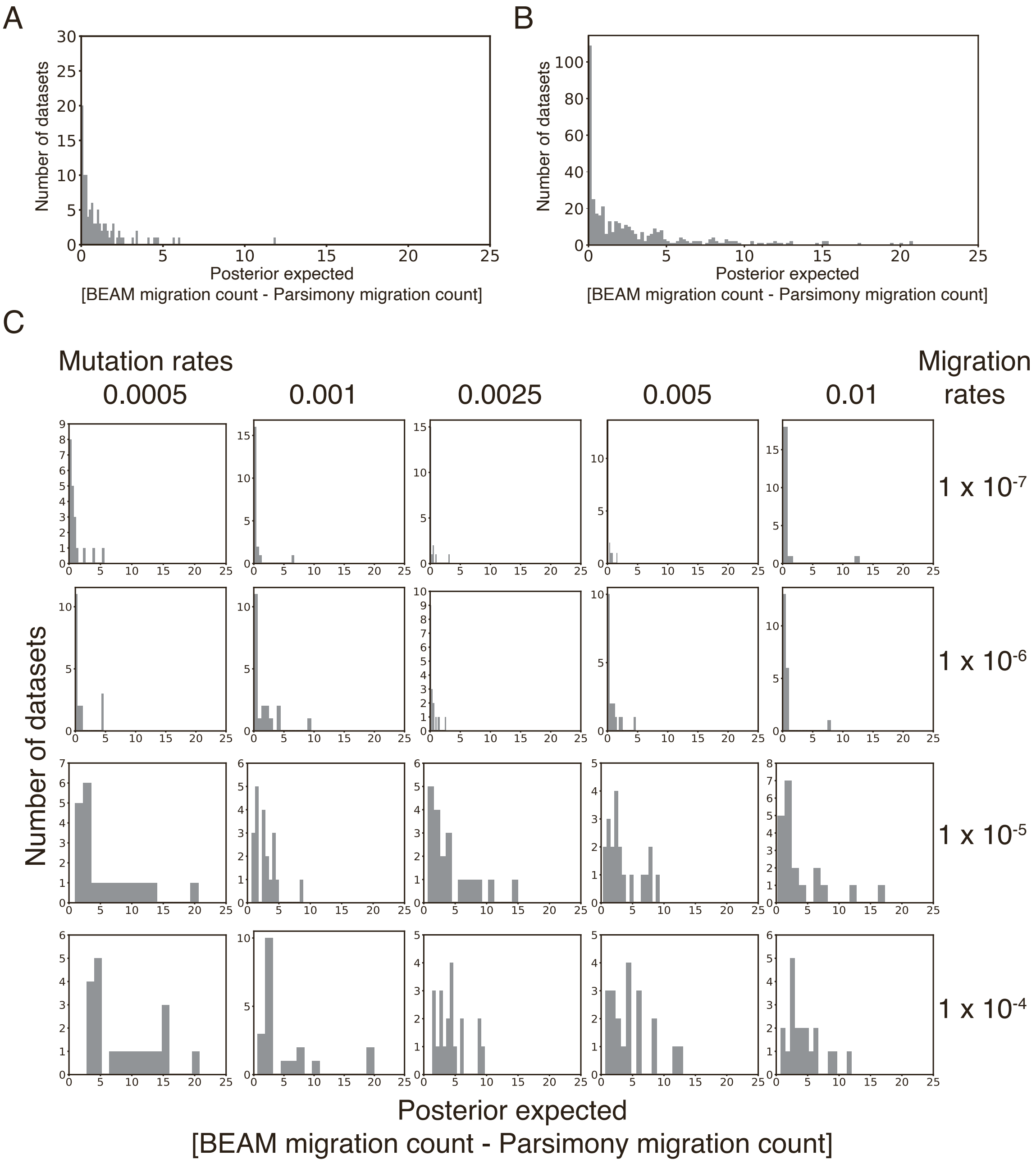

Figure S8. Posterior excess migration counts relative to parsimony-based solutions, Related to Figure 2
Histogram of the excess migrations predicted by BEAM relative to the Fitch-Hartigan parsimony solution for the same trees (see text) in: (A) the favorable parameter regime shown in **Figure 2A**; (B) the variable parameter regime shown in **Figure 2B**; and (C) the variable parameter regime stratified by mutation and migration rate.

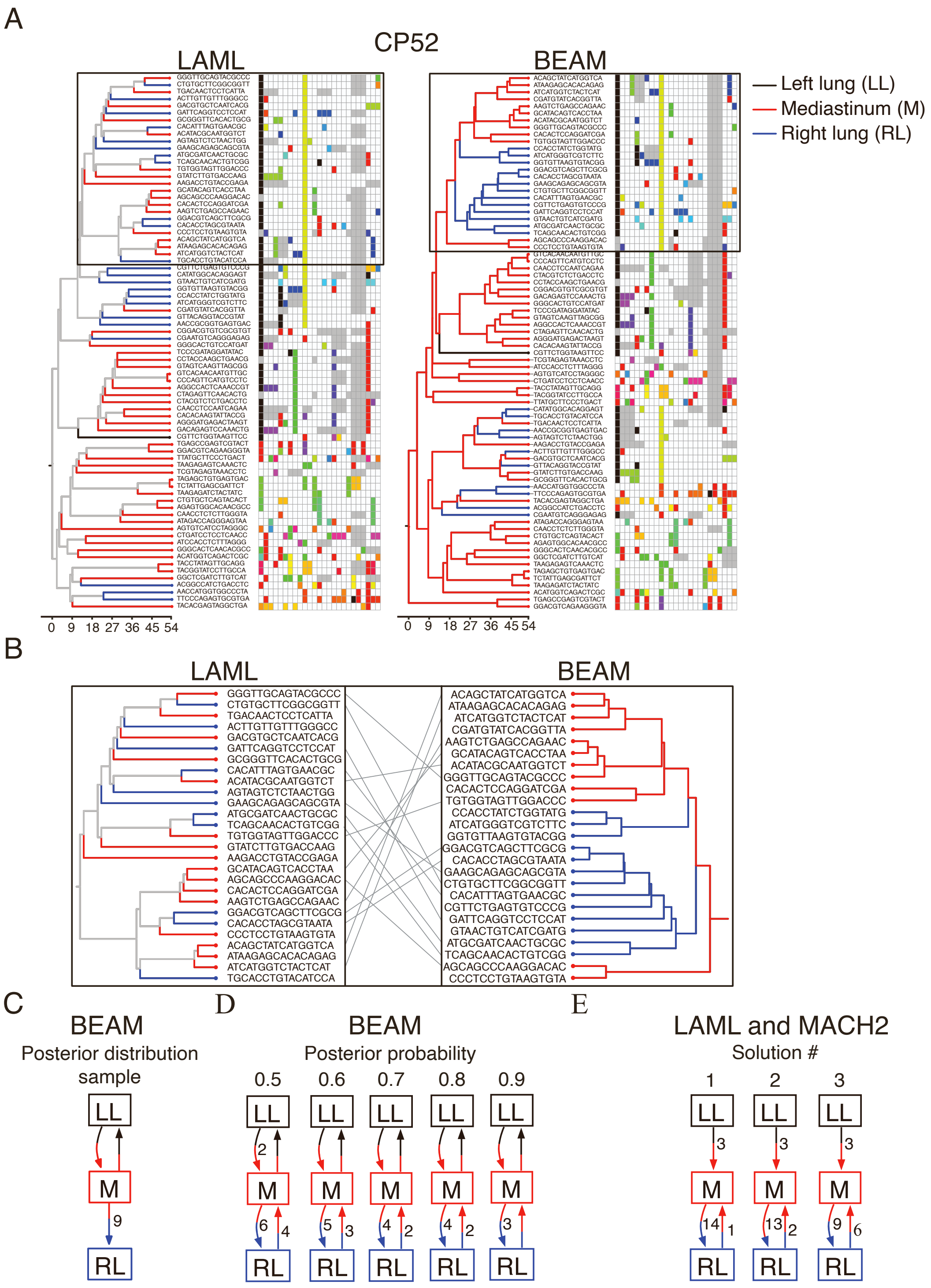

Figure S9. Example lineage trees and migration graphs with differing inferred topologies across methods, Related to Figure 3

(A) Lung cancer lineage trees for CP52 as inferred by LAML (left) and BEAM (right). LAML inferred only the tree topology, so internal nodes are gray, while BEAM resolved internal nodes by tissue. The matrix next to each tree shows barcode mutations per cell with white as unedited, gray as missing, and each color representing a mutation (with colors repeating if there are too many unique mutations). (B) Zoomed-in view of a clade with topological differences between LAML and BEAM. Gray lines map tips present for both methods. (C) Migration graph for the BEAM lineage tree posterior sample in (A). (D) BEAM migration graphs at increasing edgewise probability-retention thresholds. (E) MACH2 migration graph resolved from the LAML tree.

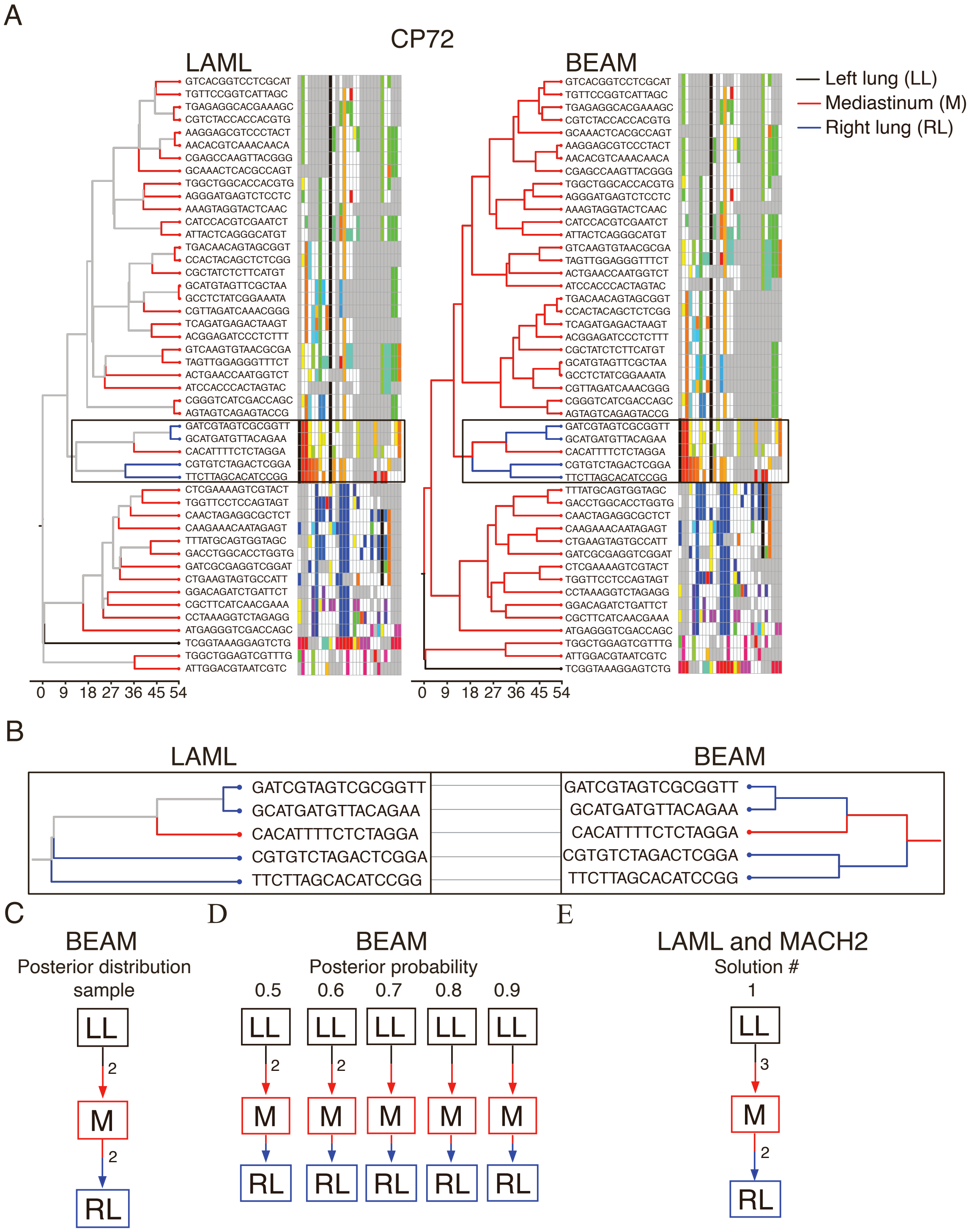

Figure S10. Example lineage trees and migration graphs with similar inferred topologies across methods, Related to Figure 3
(A–E) Same as **Figure S9** but for lung cancer CP72.

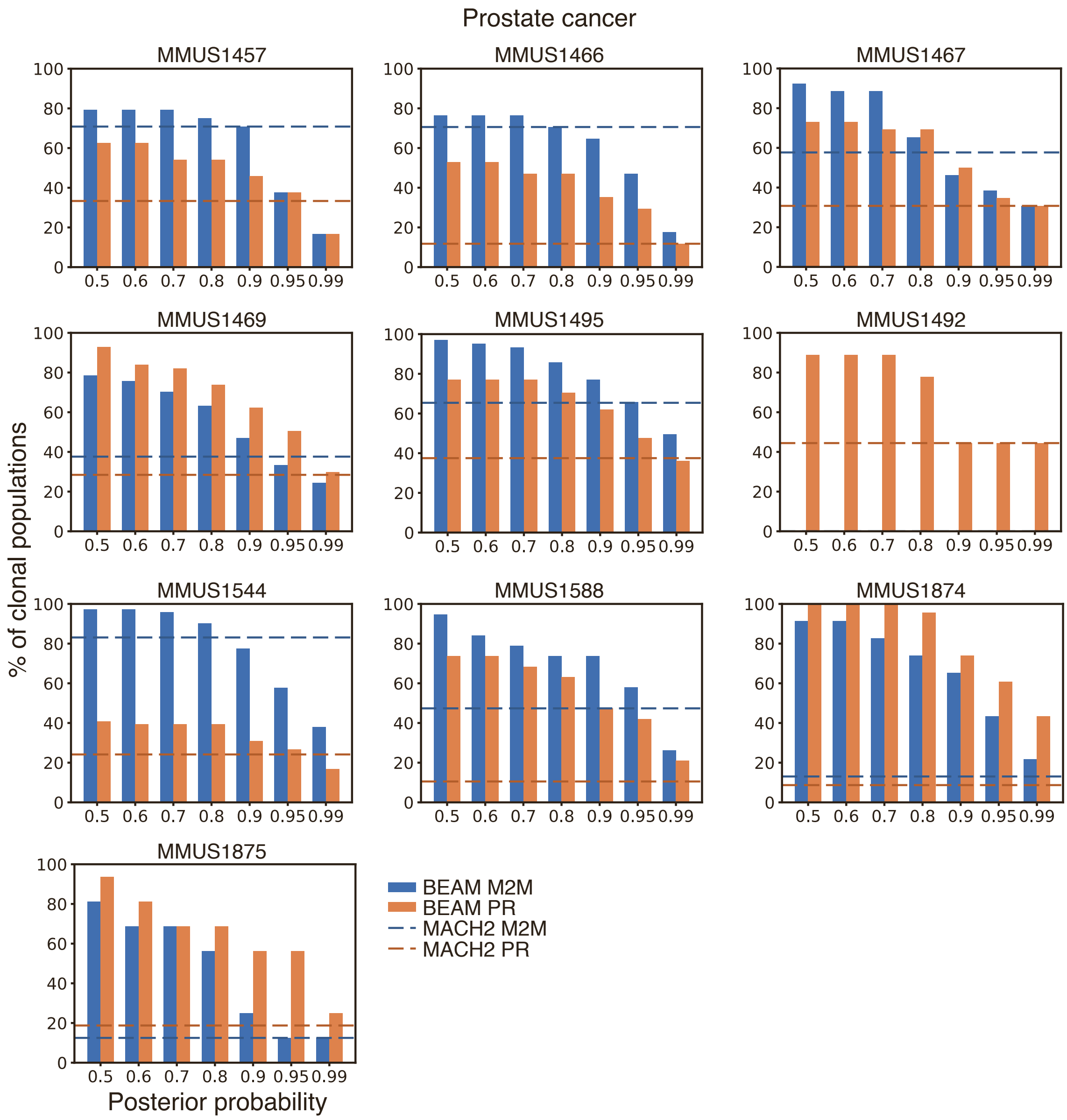

Figure S11. Detection of metastasis-to-metastasis and primary reseeding events in prostate cancer stratified by mouse, Related to Figure 3

Same as **Figure 3A** for the prostate cancer data plotted per mouse.

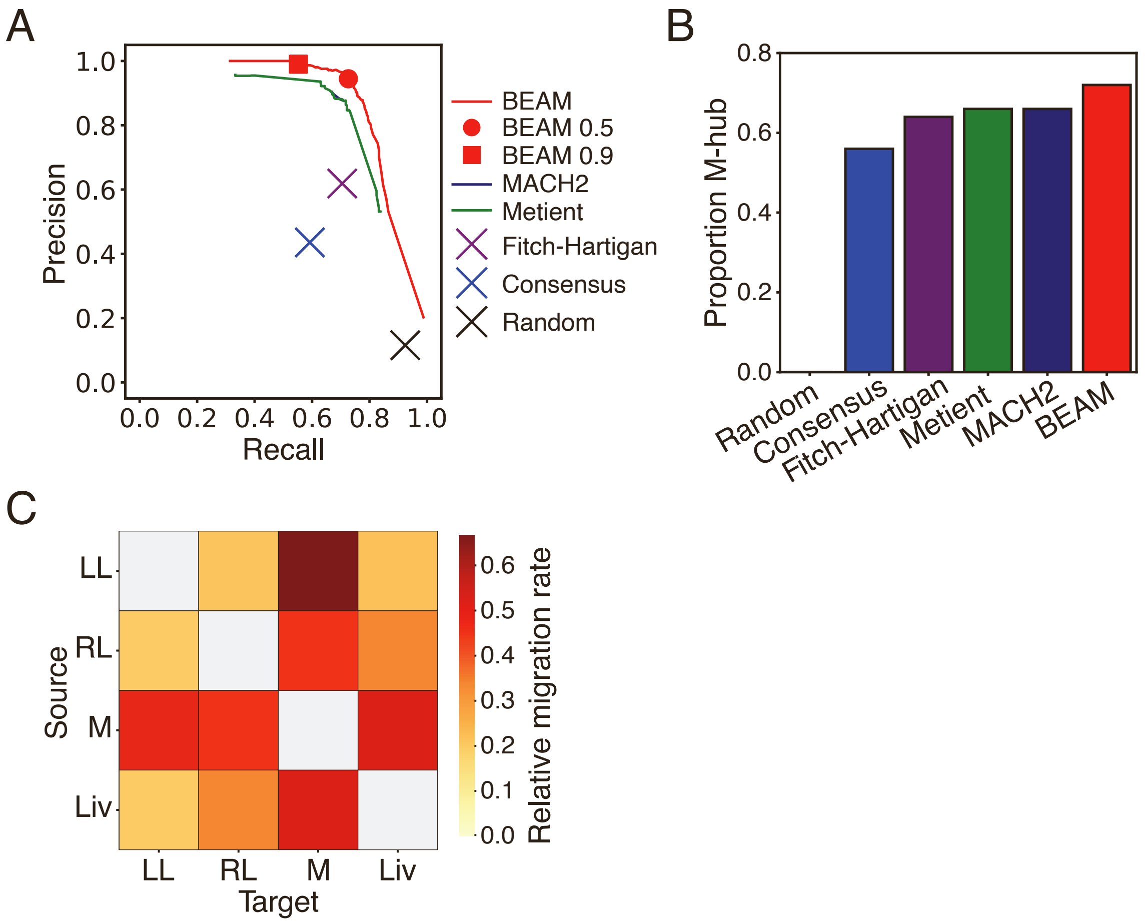

Figure S12. Simulation-based evaluation of M-hub migration patterns, Related to Figure 3 (A) Precision–recall performance for 50 simulations modeled after the real lung cancer dataset, with four tissues, LL as the primary tissue, and only LL→M migrations permitted (transition probability 1.0, all other primary-to-met transitions set to 0 to reproduce the observed M-hub pattern. All other migrations among metastatic tissues were simulated uniformly. Datasets were generated using a mutation rate of 0.0025 and migration rate of 1×10^{-6}, matching our standard conditions. We compared BEAM (showing both the curve and point estimates at posterior probability thresholds 0.5 and 0.9) with MACH2, Metient, Fitch–Hartigan parsimony, Consensus, and Random tissue-labeling baselines. (B) Proportion of simulations in which each method correctly recovered the migration graph as an M-hub, defined as LL seeding only M and subsequent migrations originating from M to other tissues. Bars show the fraction of simulations whose inferred graph matched this pattern. (C) Mean posterior instantaneous migration rates between tissues inferred by BEAM, averaged across all simulations under the normalized CTMC migration model from each source tissue to each target tissue. We use the LL, RL, M, and Liv labels to match the lung-cancer dataset, but the internal simulated tissue labels were generic as in our other simulations, so we simply reassigned labels arbitrarily while respecting the primary tissue and M-hub structure.

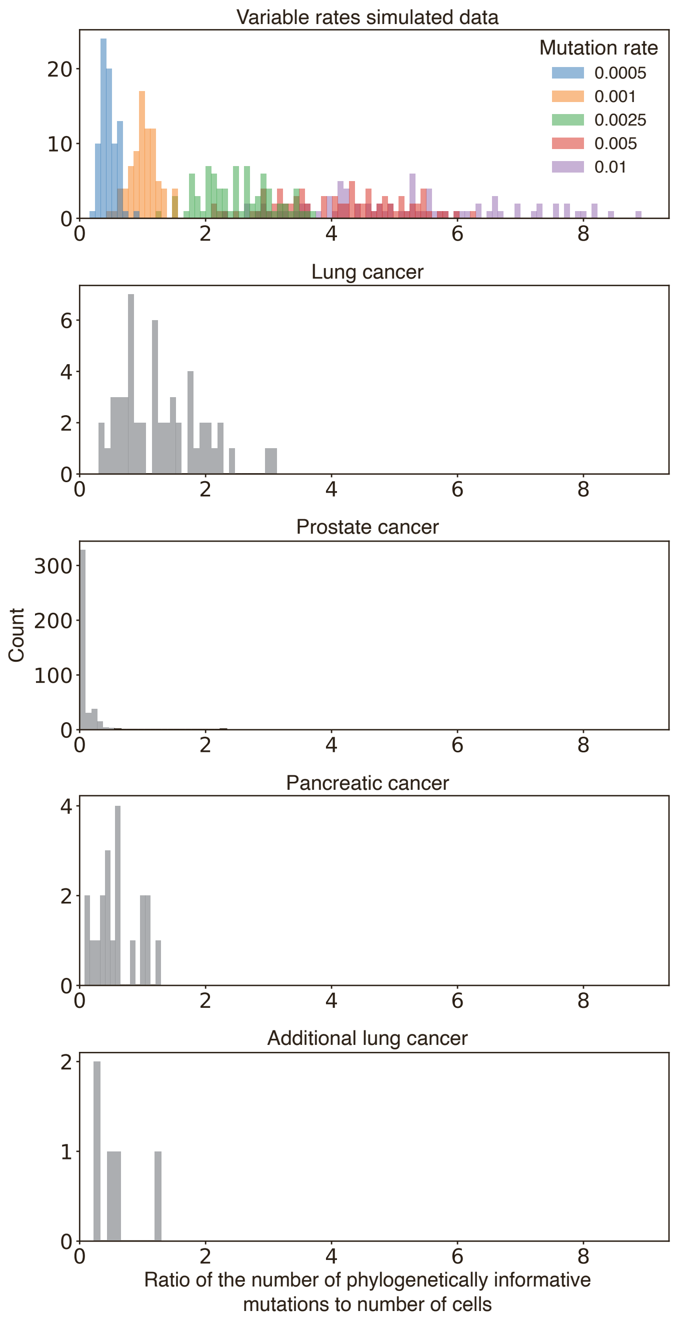

Figure S13. Phylogenetically informative mutation content across real and simulated datasets, Related to Figure 3 and Figure 4

The ratio of phylogenetically informative mutations to the number of cells (tree tips) was calculated for each clonal population in the real and variable-rate simulated datasets included in this study. “Lung cancer” and “Prostate cancer” correspond to the main datasets analyzed[S3,S3], while “Pancreatic cancer” and “Additional lung cancer” are additional external CRISPR barcode datasets[S3,S4]. At each barcode site (column) in the mutation matrix, unique informative mutations were defined as the set of those mutations present in two or more cells (rows), but not all cells. Counts were summed across barcode sites and divided by the number of cells in the clonal population. Bars in the variable-rate simulated dataset are colored by simulated mutation rate for reference.

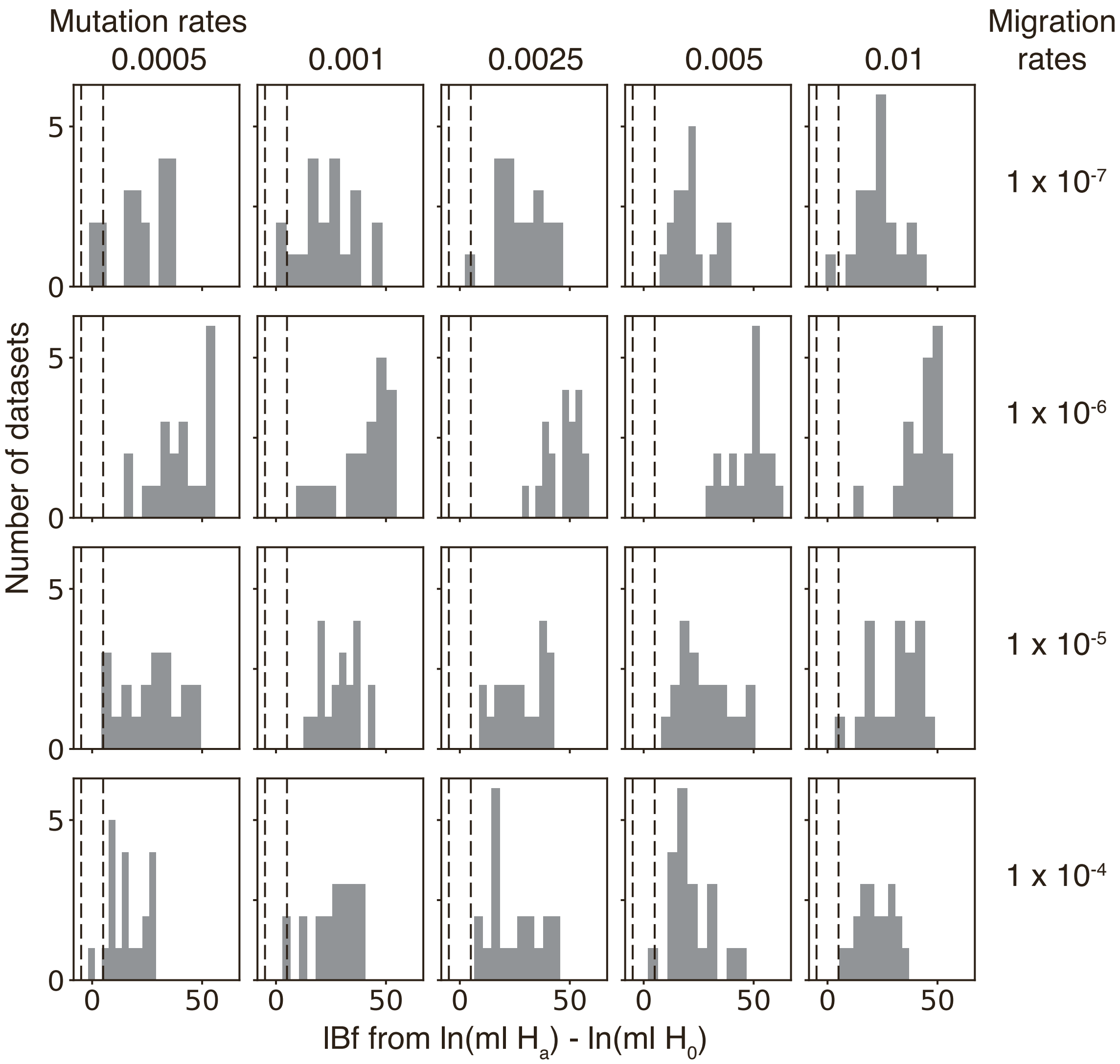

Figure S14. Information-content hypothesis testing in variable-rate simulated datasets, Related to Figure 4

Application of the random vs. GTR information content hypothesis test in **Figure 4A** to the variable rates simulated data from **Figure 2B**. The reported values are the log Bayes factor (lBf) from the comparison of the two models and the dashed lines indicate the classification thresholds at -1.1 in favor of the random model and 1.1 in favor of the GTR model.

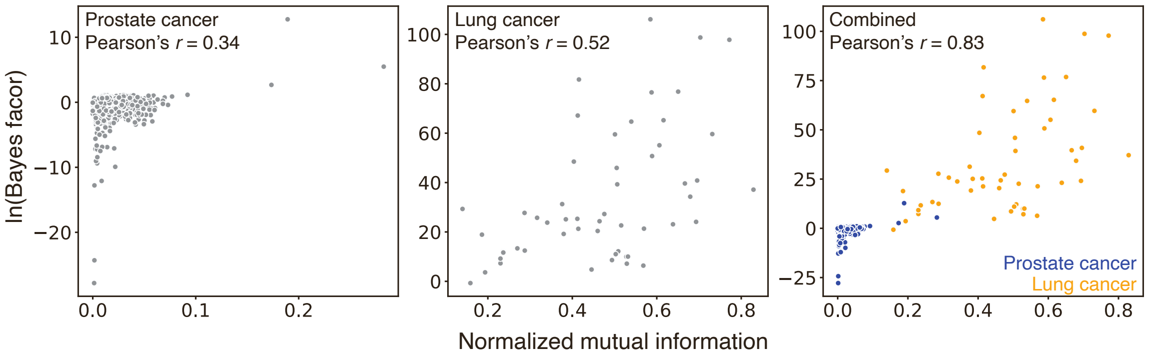

Figure S15. Relationship between Bayes factor and posterior mutual information for quantifying dataset informativeness, Related to Figure 4

Relationship between the log Bayes factor from the random vs. GTR information content hypothesis test in **Figure 4A** (y-axis) and the normalized mutual information (x-axis) computed from tissue transition count matrices across BEAM posterior distributions (see **STAR METHODS**). The prostate cancer data is shown on the left, the lung cancer data in the center, and the combined data on the right.

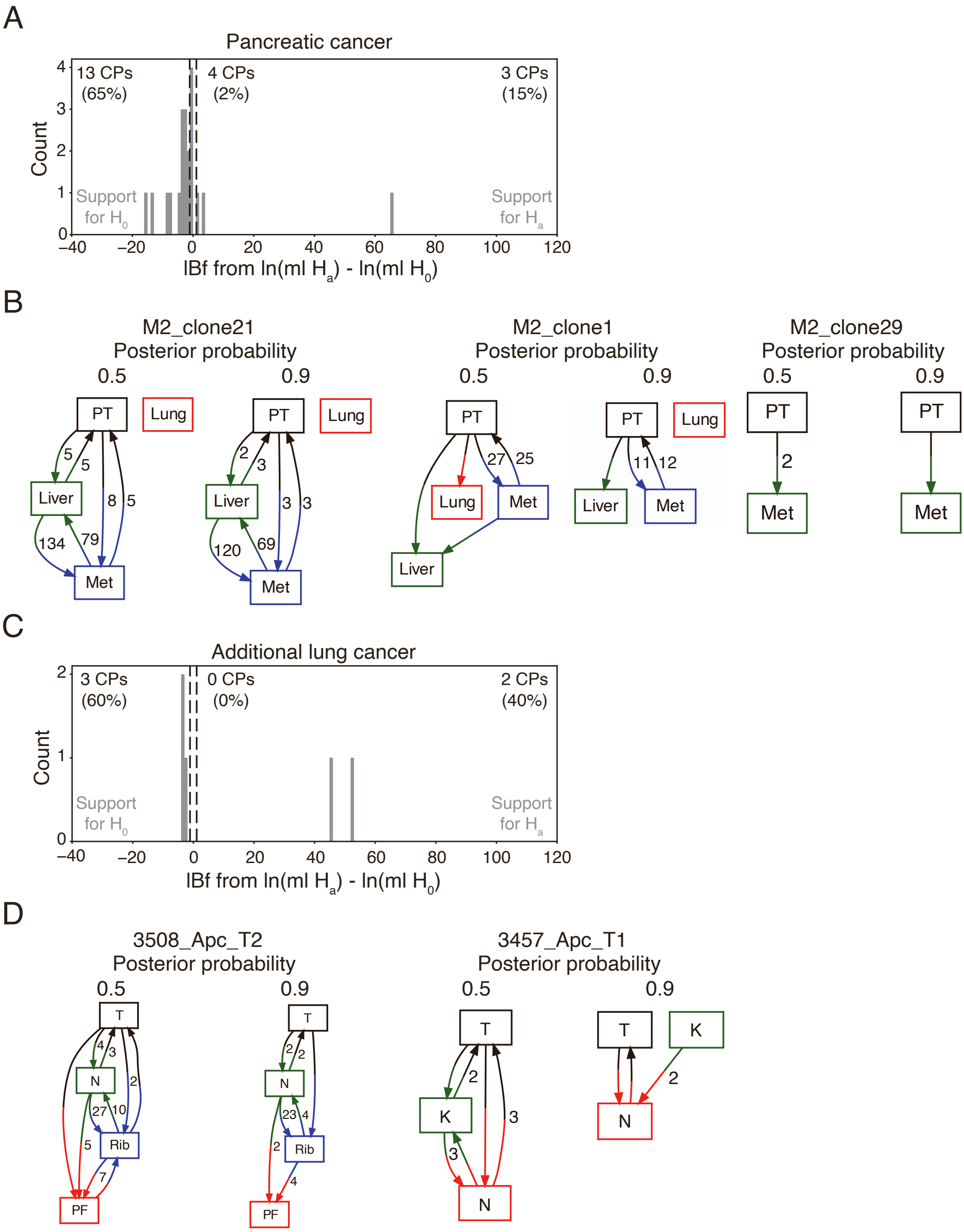

Figure S16. Information-content testing and migration graphs for additional lung and pancreatic cancer datasets, Related to Figure 4

(A) and (C) show the same analysis as in **Figure 4A** for the additional pancreatic[S4] and lung[S3] cancer datasets. (B) and (D) show the 0.5 and 0.9 edgewise probability threshold graphs for those clones passing the tests in (A) and (C) respectively. Passing clones are labeled with their respective IDs in each deposited dataset, which are formatted here as mouse_clone for the pancreatic cancer clones and mouse_genotype_clone for the lung cancer clones. Tissue label acronyms for nodes in the migration graphs are retained from the deposited dataset labels used in the original studies. PT is the primary tissue in (B) and T is the primary tissue in (D).

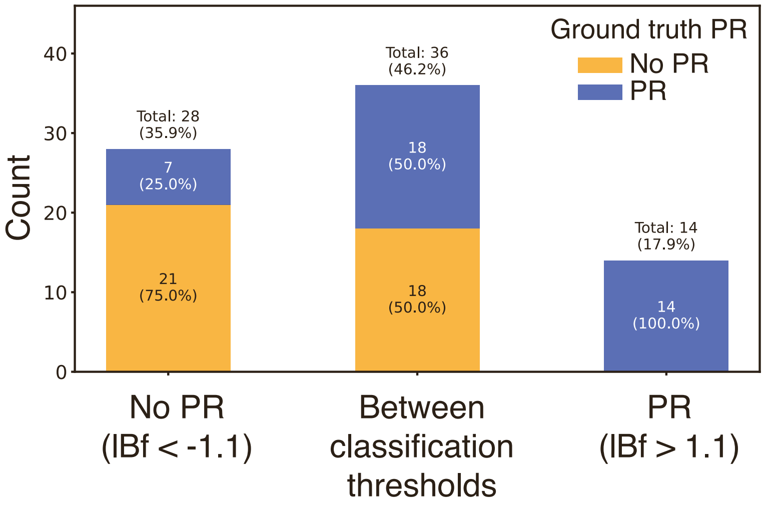

Figure S17. Performance of primary reseeding hypothesis testing on simulated data, Related to Figure 4

Simulated data were classified using a hypothesis test comparing primary reseeding (PR) and no PR models as done for real data in **Figure 4B**. Simulations were selected from the larger variable-rates simulated dataset in **Figure 2B** to include equal numbers of ground truth migration graphs with and without PR. True labels are indicated by color in the legend. Classification results are based on the log Bayes factor (lBf) where simulations are labeled positive for PR if lBF > 1.1, negative for PR if lBF < -1.1, or between classification thresholds if $-1.1 <$ lBF < 1.1.

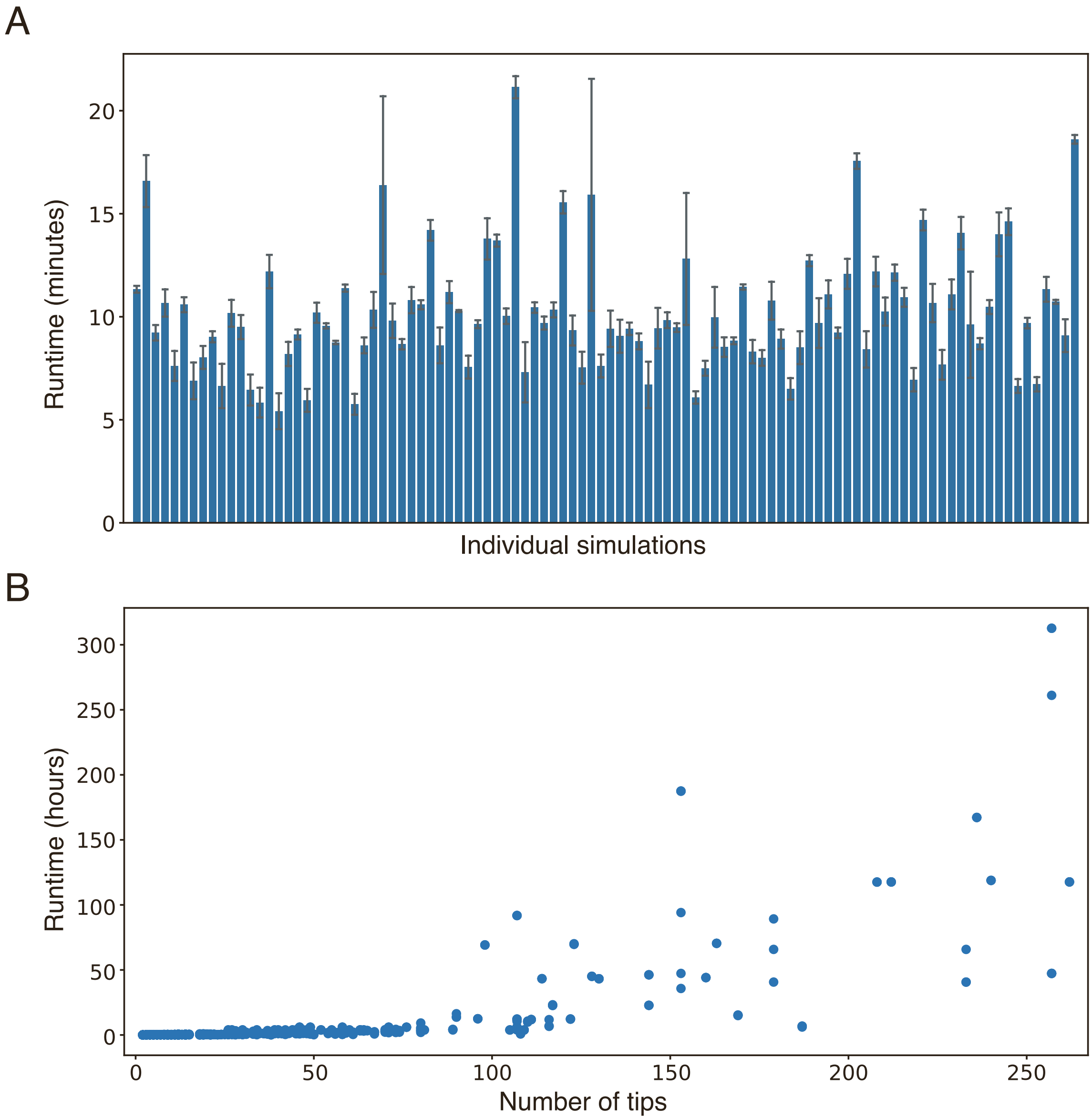

Figure S18. Runtime of BEAM across simulated and real lineage-tracing datasets, Related to Figure 2 and Figure 3
(A) Runtime of BEAM (in minutes) for the 100 simulated datasets shown in **Figure 2A**, each containing 50 tips per tree. Bars show the mean $\pm$ the standard deviation across three independent runs for each simulation. (B) Runtime of BEAM (in hours) for all MCMC chains used in the analysis of the main lung cancer[S3], main prostate cancer[S3], additional pancreatic cancer[S4], and additional lung cancer[S3] datasets in this study, all plotted as a function of the number of tips in each tree. Runtimes were estimated based on the logged runtime per million MCMC samples and the total number of MCMC samples completed at termination. All BEAM analyses were run on a high-performance computing cluster with Intel Xeon 6252, 8260, or 6432 CPU processors. Each job had access to up to five CPU threads (via BEAST 2's native multithreading) and up to 10 GB of memory, though actual memory usage was typically much lower.

SUPPLEMENTARY TABLES

Migration Event	Edgewise Probability Threshold						
	0.5	0.6	0.7	0.8	0.9	0.95	0.99
LL $\rightarrow$ Liv	13	10	8	4	2	0	0
M $\rightarrow$ Liv	27	27	25	25	19	16	9
RL $\rightarrow$ Liv	13	13	10	9	4	2	2

Table S1. Number of liver-directed migration events across posterior probability thresholds, Related to Figure 3
The number of CPs with a transition from left lung (LL), right lung (RL), or mediastinum (M) to Liv at different edgewise posterior-probability thresholds. Counts are out of a total of 35 CPs with the Liv tissue observed.

Mouse	Initial count	GTR selected	Primary reseeding selected
MMUS1469	111	0	0
MMUS1457	24	3	1
MMUS1544	71	1	0
MMUS1495	105	0	0
MMUS1466	17	0	0
MMUS1467	26	0	0
MMUS1492	9	0	0
MMUS1874	23	0	0
MMUS1875	16	0	0
MMUS1588	19	0	0
**Overall count**	421	4	1

Table S2. Per-mouse outcomes of Bayes factor hypothesis testing in prostate cancer, Related to Figure 4

The initial number of total CPs per mouse (left), the number of CPs per mouse selecting the GTR model in the random vs. GTR hypothesis test of data information in **Figure 4A** (middle), and the number of CPs per mouse that selected the primary reseeding (PR) model in the no PR vs. PR hypothesis test in **Figure 4B** (right) out of those that previously selected the GTR model (middle).
