# Supplementary material for: Bayesian inference of tissue-migration histories in metastatic cancer from cell-lineage tracing data
Source: Cell Genom. 2026 Mar 30;6(6):101193. doi: 10.1016/j.xgen.2026.101193 (PMC13261679; doi:10.1016/j.xgen.2026.101193)
Supplement: Document S3. Article plus supplemental information [file mmc3.pdf]

Cell Genomics

Technology

Bayesian inference of tissue-migration histories in metastatic cancer from cell-lineage tracing data

Graphical abstract

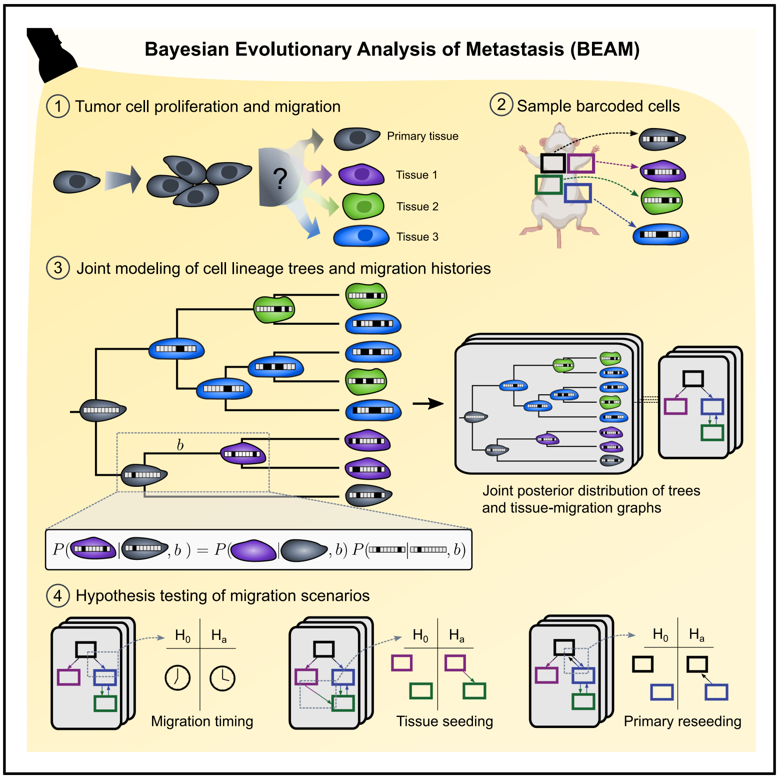

Authors

Stephen J. Staklinski, Armin Scheben, Lise M. Brault, ..., Jiawei Xing, Dawid G. Nowak, Adam Siepel

Correspondence

dgn2001@med.cornell.edu (D.G.N.), asiepel@cshl.edu (A.S.)

In brief

Staklinski et al. present BEAM, a Bayesian approach for uncovering how cancer cells spread between tissues using lineage-tracing data. By accounting for uncertainty and jointly modeling cell lineages and migration, BEAM uncovers complex patterns of metastasis across cancer types and enables hypothesis testing of competing models for how tumors spread.

Highlights

- BEAM jointly infers cell-lineage trees and tissue-migration histories
- BEAM outperforms existing methods across simulated biological scenarios
- Posterior distributions quantify migration-history uncertainty and dataset limitations
- BEAM infers complex and heterogeneous migration histories across cancer types

Staklinski et al., 2026, Cell Genomics *6*, 101193
June 10, 2026 © 2026 The Author(s). Published by Elsevier Inc.
https://doi.org/10.1016/j.xgen.2026.101193

CellPress

Cell Genomics

CellPress OPEN ACCESS

Technology

Bayesian inference of tissue-migration histories in metastatic cancer from cell-lineage tracing data

Stephen J. Staklinski,[1] Armin Scheben,[1] Lise M. Brault,[2] Rebecca Hassett,[1] Ryan N. Serio,[2] Jiawei Xing,[1] Dawid G. Nowak,[2,3,4,*] and Adam Siepel[1,5,*]

[1]Simons Center for Quantitative Biology, Cold Spring Harbor Laboratory, Cold Spring Harbor, NY 11724, USA

[2]Meyer Cancer Center, Weill Cornell Medicine, New York, NY 10065, USA

[3]Department of Pharmacology, Weill Cornell Medicine, New York, NY 10021, USA

[4]Division of Hematology and Medical Oncology, Department of Medicine, New York Presbyterian Hospital, Weill Cornell Medicine, New York, NY 10021, USA

[5]Lead contact

*Correspondence: dgn2001@med.cornell.edu (D.G.N.), asiepel@cshl.edu (A.S.)

https://doi.org/10.1016/j.xgen.2026.101193

SUMMARY

Cell-lineage tracing now enables direct study of tissue migration in metastatic cancer, but current reconstruction algorithms are limited by a reliance on strong parsimony assumptions and pre-estimated cell-lineage phylogenies. Here, we introduce a probabilistic modeling and inference framework, called Bayesian Evolutionary Analysis of Metastasis (BEAM), which provides richer information about complex metastatic histories. Based on the flexible BEAST 2 platform for Bayesian phylogenetics, BEAM infers a full posterior distribution over cell-lineage phylogenies and tissue-migration graphs, complete with timing information. We show using simulated data that BEAM reliably outperforms current methods for inference of tissue-migration graphs, especially for more complex histories. We then apply BEAM to public datasets for lung and prostate cancer, finding support for distinct modes of migration across clones and reseeding of primary tumors. Overall, BEAM serves as a powerful framework for revealing the modes, timing, and directionality of tissue migration in metastatic cancer.

INTRODUCTION

Like all populations of proliferating cells, tumors adapt to their environments through an evolutionary process involving mutation, selection, and genetic drift.[1] During the past two decades, the tools of statistical phylogenetics—originally devised to infer species trees[2–4]—have been adapted for reconstructing tumor evolution.[5–8] Recently, technologies for single-cell lineage tracing and sequencing[9–13] have inspired phylogenetic methods to reconstruct lineage histories for thousands of individual cells.[14–19] These methods have been applied not only to cancer cells but also to problems in developmental biology[20–22] and neurobiology.[23]

As these methods have advanced, interest in tumor lineage reconstruction has turned to the critical question of how cancer cells metastasize from one tissue to another. In many cancers, metastasis represents a transition from a localized tumor that can be effectively treated with radiation or surgery to a systemic disease requiring riskier and less effective treatments such as chemotherapy and immunotherapy.[24] Several common types of cancer—including breast, prostate, colorectal, urinary bladder, and kidney cancer—are highly treatable (with 5-year survival rates >90%) when they are caught before metastasis occurs but have dramatically poorer outcomes (with 5-year survival rates of ∼40% or, in some cases, much less) after metastasis.[25] The causes of cancer mortality are multifaceted and it is an oversimplification to attribute most cancer deaths to metastasis[26,27]; nevertheless, metastasis is a key event in the transition from treatable to untreatable disease.[24]

Despite the importance of metastasis, much remains unknown about how and why it occurs. It is not well understood whether metastases are initiated by single or multiple cells, whether tumor cells primarily spread through vascular or lymphatic pathways, how frequently migrations occur, and whether they predominantly arise from the primary tumor or are dispersed across tissues.[28–37] It also remains unclear why metastatic tumors appear preferentially in certain organs, in a manner that depends strongly on the cancer type, a phenomenon known as organotropism.[38–42] Strikingly, the fundamental question posed by the English surgeon Stephen Paget in his seminal article of 1889—“What is it that decides what organs shall suffer in a case of disseminated cancer?”[43]—still has no good answer.

As shown in recent studies,[34,37,44] the combination of cell-lineage tracing and phylogenetic reconstruction, typically in mouse models, promises to shed new light on these long-standing puzzles. These methods can reveal critical aspects of the rates,

This is an open access article under the CC BY license (http://creativecommons.org/licenses/by/4.0/).

routes, drivers, clonality, and specific molecular changes associated with metastatic events. Notably, the primary object of interest in these studies is typically not the cell-lineage phylogeny itself but rather the induced history of migration events, which can be summarized as a "migration graph"[45]—a collapsed version of the phylogeny with nodes representing tissues and (directed) edges representing migration events. If both the phylogeny and the tissue of residence of each cell are known, then the migration graph is simply derived by traversing the branches of the tree and recording a corresponding migration event if the parent cell and child cell occupy different tissues. The challenge is that neither the tree nor the tissues of ancestral cells are known, and uncertainty about these objects propagates to the migration graph in a complex manner. Interestingly, the problem of inferring the tissue-migration graph is related to the problem of phylogeographic reconstruction of the movements of ancestral species.[46–48] In both cases, the phylogeny provides a guide but it is the induced migration history that is of primary interest.

Current methods for reconstructing tissue-migration graphs follow a two-step process in which one lineage phylogeny is first reconstructed, and then a migration history is inferred conditional on that phylogeny.[45,46,49–51] Typically, many migration histories are compatible with a given phylogeny, so one is selected by maximum parsimony, i.e., by minimizing the number of tissue migrations, possibly together with other criteria. This approach is practical and leverages new tools for cell-lineage reconstruction, but it fails to exploit the tightly coupled nature of the problems of inferring the lineage tree and the migration graph. In particular, there are often many lineage trees compatible with the data, particularly when mutations are sparse, but some of those trees may suggest more likely migration histories. Ideally, the two problems would be solved together so that the impact on migration histories could be considered when inferring the lineage phylogeny (see El-Kebir et al.[45] for an early attempt at a joint solution).

A related problem is that, even when the lineage tree is fixed, many different migration histories are often plausible, and biological questions of interest are typically best addressed by considering a collection of possible histories rather than one of them.[49] For example, cancer biologists may be primarily interested in a higher-level question such as whether metastasis to bone is more likely than metastasis to the liver or how frequently reseeding of the primary tumor occurs. Ideally, an analysis method would permit evaluating the cumulative support for such a question relative to a probability distribution of possible graphs given the data, but no such method yet exists.

We address these limitations by introducing a joint probabilistic model for cell-lineage phylogenies and tissue-migration graphs together with a procedure for Bayesian statistical inference. Our software implementation, called Bayesian Evolutionary Analysis of Metastasis (BEAM), is the first to support inference of the full posterior distribution over lineage trees, tissue-migration graphs, and associated parameters. BEAM is implemented using the BEAST 2 (Bayesian Evolutionary Analysis by Sampling Trees 2) platform for Bayesian phylogenetics,[52] and it leverages BEAST 2's optimized procedures for Markov chain Monte Carlo (MCMC) inference of phylogenetic parameters. In addition, BEAM supports Bayesian hypothesis testing of any derived property of the migration graph, such as the presence of particular edges. We show using simulated data that BEAM accurately reconstructs features of the true migration graph over a broad range of parameters, consistently outperforming available methods. In addition, we apply BEAM to recently published lineage-tracing datasets for lung- and prostate-cancer models and show that it reveals important migration-graph features that were not evident by maximum parsimony. Overall, we show that BEAM is a promising new approach for dissecting the dynamics of metastasis across diverse cancer types.

DESIGN

The problem of inferring both the cell-lineage phylogeny and tissue-migration graph can be recast as inferring a "colored" lineage tree, in which each node (cell) is assigned a color representing its tissue of residence (Figure 1). A coloring of nodes unambiguously induces a tissue-migration graph because migration edges correspond to tree branches whose adjoining nodes have mismatching colors. Colors (tissues) of the tips of the tree are generally known from the sampling procedure, so the problem reduces to inferring a coloring of internal nodes.

To define a probabilistic model for this joint process, it is sufficient to extend a model for CRISPR-based lineage tracing to allow for changes in colors (tissues), together with the accumulation of mutations, along branches of the tree. We extended a simplified version of the continuous-time Markov chain (CTMC) model for CRISPR-Cas9 barcode editing implemented in TiDeTree[53] (see also Chu et al.[19]) with a second, conditionally independent CTMC that describes tissue-migration events. This second CTMC can be parameterized in many ways, but our default is a general time reversible (GTR) parameterization.

With this model, time-dependent transition probabilities along tree branches—representing joint probabilities of mutations and tissue migrations—are products of standard rate-matrix exponentials, and the full likelihood of the data can be calculated using Felsenstein's pruning algorithm.[54] While straightforward, this approach enables joint inference of lineage phylogenies and migration graphs by MCMC sampling, avoiding the two-step approximation of existing methods (Figure 1). We implemented this model in a package called BEAM, which is built on the BEAST 2 platform.[52] An example of the output of BEAM is shown in Figure S1.

RESULTS

BEAM recovers tissue-migration histories from realistic simulations

To evaluate the accuracy of BEAM in reconstructing migration histories, we combined existing simulation tools[14,45,55] to model single-cell birth-death dynamics, tissue migration, and CRISPR barcode editing. We first generated synthetic multi-tissue barcode sequence data under a regime favorable for migration-graph reconstruction, with a relatively low migration rate (1×10^{-6} per cell) and a relatively high mutation rate (0.0025 mutations per barcode site per cell division). We then assessed the precision and recall of BEAM at predicting individual edges of each simulated migration graph across 100 replicates. For comparison, we also evaluated the MACHINA,[45] PathFinder,[49]

CellPress
OPEN ACCESS

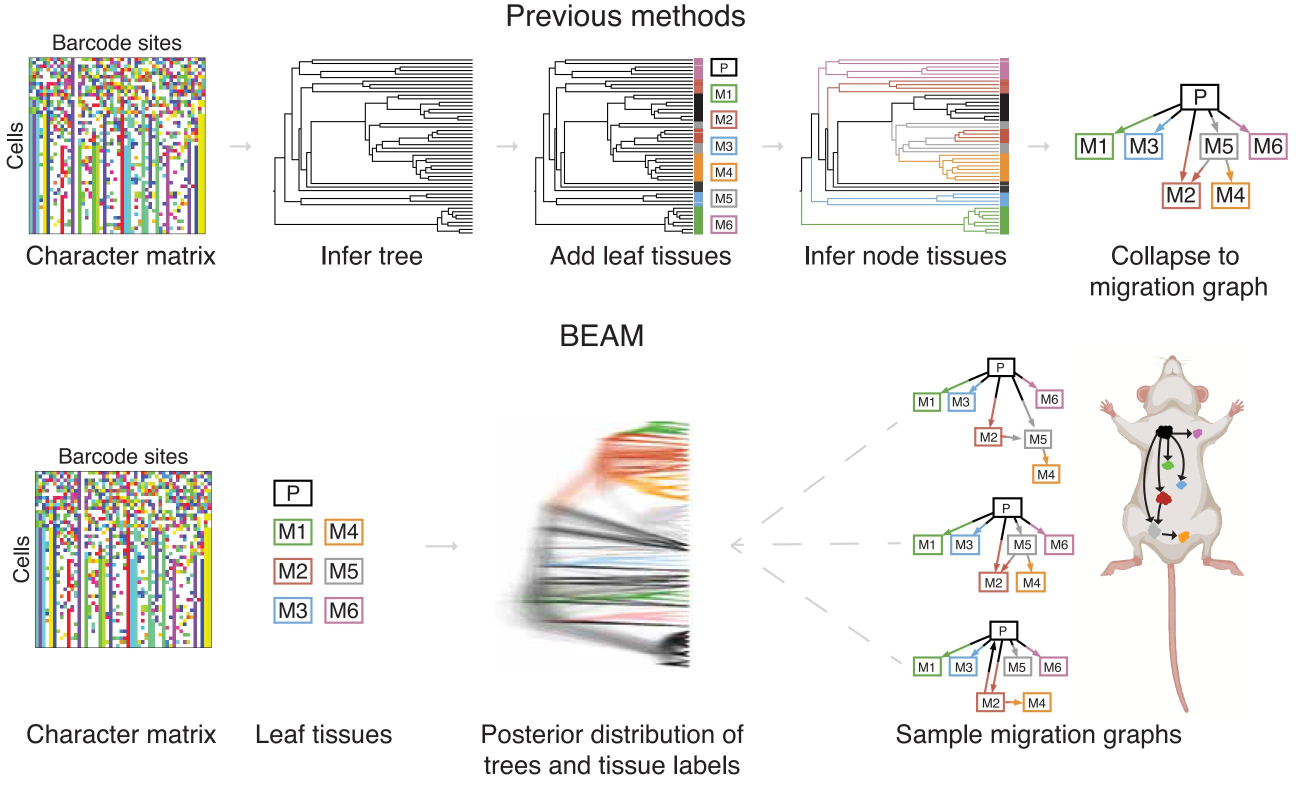

Figure 1. Bayesian inference of lineage trees and tissue-migration histories

Existing methods infer a fixed lineage tree from the mutation matrix, assign observed tissue labels to leaves, and reconstruct one or a few migration graphs. By contrast, BEAM jointly models lineage trees and tissue migration, sampling from a joint posterior distribution. In the character matrix, rows represent cells, columns represent barcode sites, and colors are mutations. Tissue labels denote primary (P) and metastatic (M1–M6) tissues. See also Figure S1.

Metient,[50] and MACH2[51] migration-graph reconstruction methods. As a baseline, we assigned tissues (colors) to ancestral nodes by simple Fitch-Hartigan parsimony,[56,57] the most common descendant tissue (consensus), or a random tissue (random).

All published methods—except PathFinder, which infers its own phylogeny—require a pre-estimated tree. Our default inputs were phylogenies inferred by maximum likelihood using LAML (Lineage Analysis via Maximum Likelihood).[19] To evaluate sensitivity to the provided phylogeny, however, we also assessed performance using a tree inferred by Cassiopeia-Greedy under a parsimony assumption[14] or the ground-truth simulated tree.

We found that BEAM performed well, achieving nearly perfect edgewise precision (>95%) up to a recall rate of ~70%, after which the precision declined rapidly (Figure 2A). In this parameter regime, MACHINA, PathFinder, Metient, and MACH2 generally exhibited comparable but slightly less favorable tradeoffs between precision and recall. Notably, however, MACHINA and PathFinder—because they produce a single reconstructed migration graph—only appear as points on a precision/recall graph. Both Metient and MACH2 can report multiple solutions, but we found that they tended to be similar and fall in a narrow range on the graph, more so for MACH2 than Metient. By contrast, BEAM permits a broad range of tradeoffs between precision and recall by allowing the user to choose the threshold for the estimated posterior probability for each edge. All methods outperformed the consensus and random baselines, although the Fitch-Hartigan parsimony approach was sometimes competitive. For comparison with previous publications,[45,50,51] we also computed the F1 score for all methods, using a posterior-probability threshold of 0.5 for each edge in Metient, MACH2, and BEAM (Figure S2). By this metric, the rank order of methods was similar and BEAM maintained a significant advantage.

Notably, the differences in performance between BEAM and other methods became more pronounced when a lower-quality phylogeny from Cassiopeia-Greedy was used as input. Conversely, when other leading methods were given the ground-truth simulated tree, the performance of MACH2 and Metient was comparable to BEAM. Interestingly, the reconstruction accuracy of the trees themselves, as measured by Robinson-Foulds distance, was also highest for BEAM, followed by LAML, and then Cassiopeia-Greedy (Figure S3). Thus, BEAM is able to improve both lineage phylogenies and migration graphs by reconstructing them together. Examples of ground-truth migration graphs and BEAM-inferred graphs are provided in Figure S4.

To see whether these trends continued with other choices of parameters, we carried out experiments with barcode-mutation rates ranging from 0.0005 to 0.01 mutations per site per cell

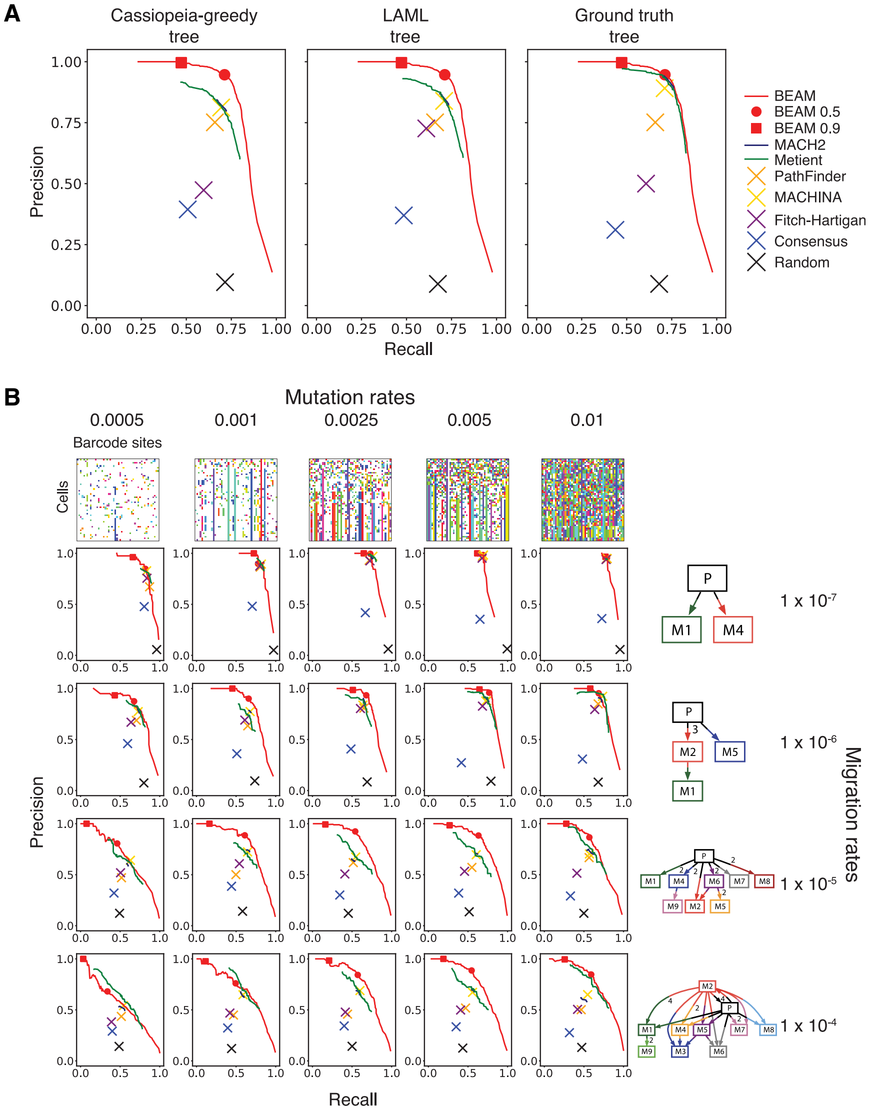

Figure 2. Precision-recall benchmarking across simulated regimes

(A) Precision-recall curves for simulated migration graphs (migration rate 1×10^{-6}; mutation rate 0.0025 per site per division). The BEAM curve is obtained by varying a threshold for the posterior probability of each edge (0.5 and 0.9 thresholds shown as points). Points are shown for the single-solution methods MACHINA[45] Path-Finder,[58] Fitch-Hartigan parsimony, and two baseline methods (consensus and random; see text). Curves are shown for MACH2[51] and Metient,[50] but they are restricted in length. Values represent means over 100 simulations and are conditioned on the input-tree method indicated above each panel. Because PathFinder and BEAM do not require a fixed input tree, their results are identical across panels.

(B) Precision-recall curves under varying mutation and migration rates using LAML-based input trees. Representative mutation matrices and migration graphs are shown.

See also Figures S2, S3, S4, S5, S6, S7, S8, and S18.

division and migration rates ranging from 1×10^{-7} to 1×10^{-4} per cell, executing 20 replicates for each combination and using pre-estimated phylogenies from LAML as the inputs for other methods. These scenarios cover a range from many mutations and few migrations, yielding well-resolved lineage trees and simple migration histories, to few mutations and many migrations, where histories are more complex and uncertainty about them should be greater.

As expected, the prediction performance of most methods improved with increasing mutation rate and decreasing migration rate (Figure 2B). Not surprisingly, all but the baseline methods performed well at high mutation and low migration rates (top right of Figure 2B) but performed considerably worse at low mutation and high migration rates (bottom left of Figure 2B). Nevertheless, BEAM outperformed all other methods across all parameter values. Interestingly, the performance improvement from BEAM was most pronounced at intermediate-to-high mutation and migration rates (particularly toward the center and bottom-right of Figure 2B), indicating that the advantages of the Bayesian approach were most evident when there was at least a modest amount of phylogenetic signal and substantial uncertainty about migration-graph structure. This may reflect the increased number of metastasis-informative mutations—those occurring on metastasis edges (Figure S5). In this regime, MACHINA, Metient, and MACH2 also improved on Fitch-Hartigan parsimony. When we stratified our simulated data based on features of the underlying ground-truth migration graphs, BEAM maintained performance across variable migration counts (Figure S6A), co-migration counts (Figure S6B), number of tissues (Figure S6C), monoclonal or polyclonal seeding events (Figure S6D), metastatic-to-metastatic seeding events (Figure S6E), and primary reseeding events (Figure S6F). Overall, BEAM performed well across a range of migration dynamics and proved to be robust to variable levels of information content in the data.

Although these analyses examined mutation and migration complexity, they did not capture the impact of indel-driven barcode site loss. To evaluate this effect, we reused the 100 simulations in Figure 2A and regenerated barcode data under increasing heritable silencing rates. In these experiments, BEAM remained the top-performing method across missing-data levels, retaining reasonable accuracy up to ~90% missing data (Figure S7), likely because it can draw on tissue labels during tree reconstruction when mutations are sparse. In contrast, all other methods degraded markedly at higher missing-data levels.

To understand how BEAM's predicted migration graphs differed from those reconstructed by parsimony, we carried out a direct comparison on the same trees. We used BEAM to sample lineage trees, and then, for those trees, we calculated both the number of migration events predicted by BEAM and the minimum number possible according to Fitch-Hartigan parsimony.[34] The difference between these quantities is a measure of the excess migrations predicted by BEAM. Not surprisingly, in favorable regimes for migration-graph reconstruction, this excess was low, with values of zero in most cases and occasional deviations of 1–3 migrations (Figure S8A). With lower mutation rates or higher migration rates, however, we observed larger excesses, with the BEAM predictions having as many as five, and sometimes 15 or more, events beyond the minimum possible (Figures S8B and S8C). Given the clear performance advantage of BEAM in this regime (Figure 2B), this observation implies that parsimony assumptions tend to break down when migration rates are high or mutational information is limited, leading to underestimation of migration events. In contrast, BEAM relaxes those assumptions, allowing the data to inform a broader range of plausible histories.

BEAM reveals complex tissue-migration patterns in lung and prostate cancer

Having demonstrated that BEAM performed well on simulated data, we turned to real data from two recent applications of lineage tracing in mouse models of cancer metastasis, one in lung[34] and one in prostate.[37] Despite differences in their strategies for cancer initiation (an orthotopic xenograft model of lung cancer in immunodeficient mice vs. a somatically engineered mouse model of prostate cancer in immunocompetent mice) and barcode sequencing (single-cell RNA sequencing vs. bulk sequencing of PCR-amplified DNA), these studies both produced mutation matrices representing barcoded cells from distinct tissues. In both cases, cells were grouped by barcode similarity into clonal populations (CPs) representing lineage trees from distinct founder cells.

The two datasets had previously been analyzed using different parsimony strategies (Fitch-Hartigan parsimony in Quinn et al.[34] and MACHINA in Serio et al.[37]). For a uniform comparison point for BEAM, we reanalyzed them using LAML[19] (instead of Cassiopeia[14]) for lineage-tree reconstruction and MACH2[51] for migration-graph inference. The tissue-migration graphs reconstructed by BEAM and MACH2 were broadly similar with somewhat more complexity evident in the BEAM reconstructions on average, consistent with our simulation results. For example, in the 0.5-posterior-probability threshold graphs across lung-cancer CPs, MACH2 averaged 12.8 migrations and 3.5 co-migrations, while BEAM averaged 13.9 migrations and 4.6 co-migrations; across prostate-cancer CPs, MACH2 averaged 8.2 migrations and 2.8 co-migrations, compared to BEAM averages of 16.6 migrations and 5.4 co-migrations. When we examined individual CPs, we found that, in some cases, the two methods predicted quite different evolutionary histories (e.g., Figure S9), but in other cases the predictions were similar (e.g., Figure S10). CPs varied considerably in size, mutation content, and migration-graph complexity between the two datasets, providing diverse scenarios to evaluate BEAM.

A key open question is whether migration events predominantly arise from the primary tumor or whether, by contrast, migrations occur at appreciable frequencies between metastatic sites or from metastatic sites back to the primary tumor. The original lung-cancer analysis[34] reported high rates of both metastasis-to-metastasis (M2M; ~90%) and primary reseeding (PR; ~65%) events. BEAM and MACH2 were reasonably consistent with the previous analysis but with lower estimates of the incidence of PR events: MACH2 reported ~95% M2M and ~50% PR, and, at a posterior-probability threshold of 0.8, BEAM reported ~94% and ~45%, respectively (Figure 3A). Notably, however, the BEAM predictions were quite sensitive to the edgewise posterior-probability threshold, with the rate of M2M events varying from ~96% at a threshold of 0.5 to ~66% at a threshold of 0.99, and the rate of PR events varying from ~53% to ~13%. MACH2 typically produced a small set of similar graphs, making results largely insensitive to the edgewise posterior-probability threshold. Overall, our Bayesian framework appears to assign high uncertainty to these secondary migration events, which generally occur later in tumor development and have weaker support in the mutation matrix. In particular, our most conservative estimates of the rate of PR events, at ~13%–23%, are several times lower than the previous estimate of ~65%. Nevertheless, our analysis supports that both M2M and PR events occur with non-negligible frequency.

The prostate-cancer data differed in several ways from the lung-cancer data. For various reasons—including the use of immunocompetent mice, a DNA readout, and a longer-duration experiment (up to 60 weeks) with more opportunity for drop-out of mutated cells—many fewer barcode mutations are captured in this system, making the phylogenetic inference problem more challenging. In addition, a more diverse set of organs was sampled in this study, including the prostate, liver, lungs, bones, bladder, and lymph nodes, whereas the lung-cancer data were dominated by three organs in close proximity (right lung [RL], left lung [LL], and mediastinum [M]). In part for these reasons, and likely also owing to some differences in their analysis pipeline, Serio et al.[37] detected M2M and PR events in only ~7% and ~0.3% of CPs, respectively (by our re-analysis of their migration graphs).

In our reanalysis, both BEAM and MACH2 detected substantially higher rates of M2M and PR events in the prostate-cancer dataset (Figures 3A and S11). MACH2 found M2M events in ~54% of CPs and PR events in ~28% of CPs. At a posterior-probability threshold of 0.8, BEAM estimated even higher frequencies of ~74% and ~65%, respectively. As with the lung-cancer data, however, BEAM's estimates depended strongly on the choice of threshold, declining from ~87% to ~32% for M2M and from ~75% to ~28% for PR as the threshold increased, indicating that many of the M2M and PR events had weak support in the data. As discussed in the next section, these estimates are undoubtedly influenced by limits in the mutational information available in this dataset, but overall they suggest that the previous analysis may have been somewhat conservative in detecting secondary migration events.

One advantage of our Bayesian inference approach is that it naturally allows for estimation of the times at which tissue-migration events occur, within the limits of a molecular-clock

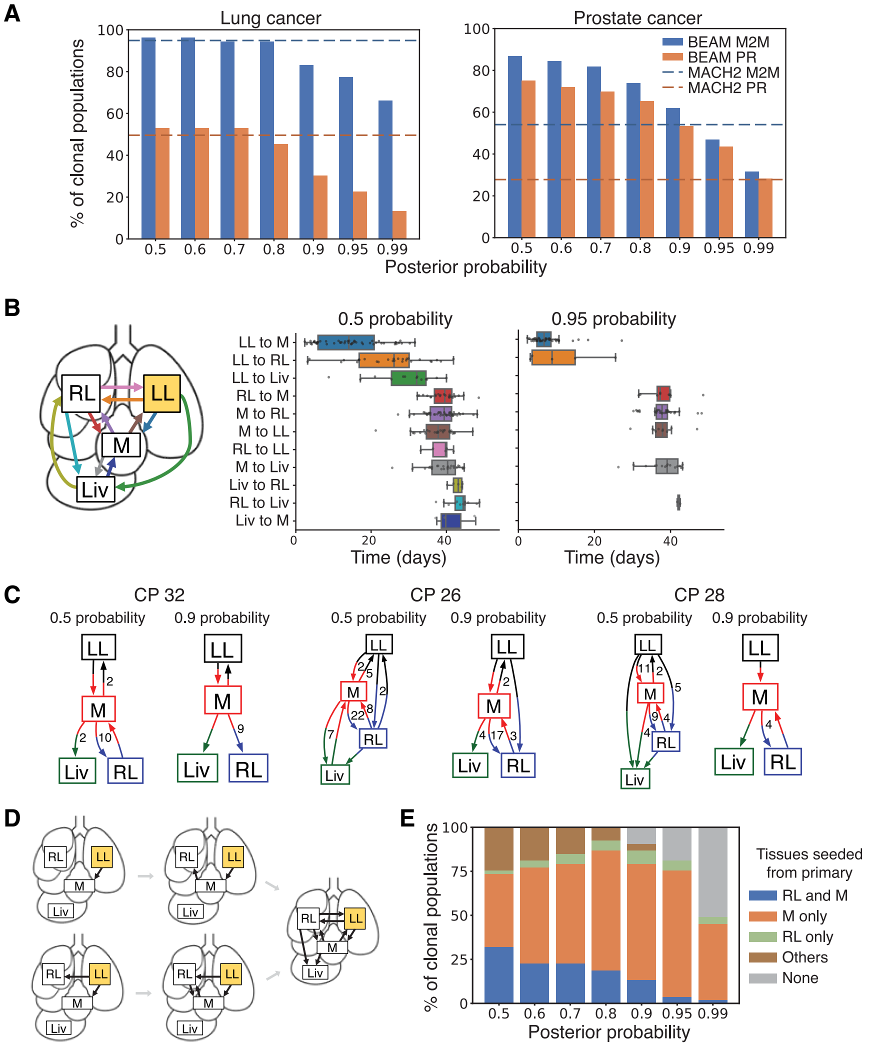

Figure 3. Metastatic progression patterns in lung and prostate cancer

(A) Fractions of clonal populations (CPs) with detected metastasis-to-metastasis (M2M) or primary reseeding (PR) events by BEAM across edgewise posterior-probability thresholds for lung[34] (left) and prostate[37] (right) datasets. MACH2[51] estimates (threshold invariant) are shown as dashed lines.

(B) BEAM-estimated migration times in the lung-cancer data at 0.5 and 0.95 edgewise posterior-probability thresholds. Boxplots show distributions of expected migration times per edge type per CP, averaged over posterior samples of branch midpoint times.

(C) Example 0.5 and 0.9 threshold graphs for lung CP32, CP26, and CP28, illustrating M-hub and direct left lung (LL) → right lung (RL) models. Edge labels indicate multiplicity (>1 lineage-tree branches).

(D) Representative progression of mediastinum (M)-hub and direct LL → RL models.

(E) Classification of all lung-cancer CPs as posterior-probability threshold increases: LL → RL and LL → M (RL and M), M only (LL → M), RL only (LL → RL), and finally whether LL seeded any other tissues (others or none). Liv = liver.

See also Figures S9, S10, S11, S12, and S13 and Table S1.

assumption. When applying BEAM migration timing analysis to the lung-cancer dataset, we noticed that the primary LL tissue was often predicted to seed the lymphatic M and RL tissues early in disease progression (Figure 3B). In further examination of the inferred migration graphs, we found that early migrations followed one of two distinct patterns: one in which M was the only site seeded from LL and acted as a hub for all other migrations and another in which both M and RL were seeded from LL (Figure 3C). Here, we used simulated data to show that BEAM more accurately detects the M-hub migration pattern than other methods and infers relative migration rates that are consistent with expectations (Figure S12). Although the early dynamics differed between these patterns, both eventually resulted in widespread dissemination (Figure 3D). The proportion of CPs in each pattern changed with the posterior-probability threshold, but nearly all CPs fit into one of the two (Figure 3E). We also observed that metastasis to the liver (Liv) was most likely to occur via M, rather than from LL or RL (Table S1). Overall, these patterns were broadly consistent with a previously reported principal-components analysis,[34] but BEAM provided additional information about migration event timing, further revealing the patterns by which these tumors spread.

Bayesian hypothesis testing of higher-level questions in metastasis

The previous section illustrates a recurring issue: while methods generally produce full tissue-migration graphs, investigators are often interested in higher-level questions, such as the frequency of M2M or PR events, or whether or not the M acted as a migration hub. We extended BEAM to address questions of this kind by averaging over possible migration graphs and weighting them by their posterior probabilities using the framework of Bayesian hypothesis testing. In this way, particular hypotheses of interest can be tested without relying on one or a few reconstructed migration graphs, each with high levels of uncertainty.

To illustrate the utility of this approach, we returned to questions that arose in our reanalysis of the lung and prostate-cancer datasets. First, we sought to test whether or not the prostate-cancer dataset supported the hypothesis of PR, given the discrepancies between methods and across BEAM posterior distributions. In this case, however, we had the additional challenge of sparse mutational data, owing to constraints of the prostate-cancer mouse model. Indeed, we observed that a majority of the CPs exhibited either no phylogenetically informative mutations or similar numbers to the lowest mutation-rate category in our simulations, where inference was challenging (left of Figure 2B). The lung-cancer data was more informative by this

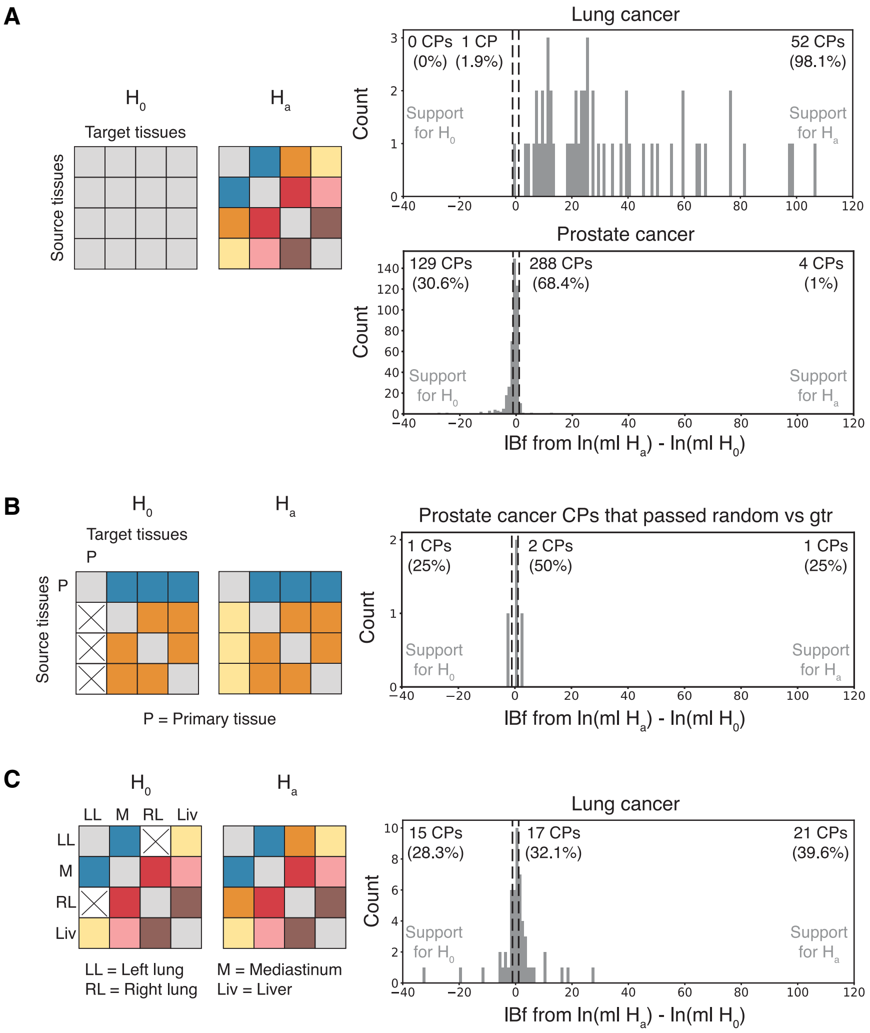

Figure 4. Bayes-factor testing of dataset informativeness and migration structure

(A) Hypothesis test of tissue-migration information comparing an alternative GTR model (right) against a random null model (left) in the lung and prostate datasets.

(B) Test of a model allowing PR (right) against a null model prohibiting it (left) in prostate CPs that passed (A).

(C) Test of a full GTR model allowing all migration types (right) against a null model prohibiting direct LL → RL seeding (left). CPs were classified by log Bayes factor (lBf) as supporting the null, the alternative, or neither, using thresholds lBf <-1.1 and lBf >1.1 (vertical dashed lines).

See also Figures S13, S14,S15, S16, and S17 and Table S2.

measure but still on the low end of our simulated mutation-rate categories. We observed similar limitations in mutation content in additional published datasets for metastatic pancreatic[44] and lung[35] cancer (Figure S13), suggesting that generating enough mutations to enable robust migration-graph inference remains a general challenge in the field.

We therefore defined an initial hypothesis test to distinguish CPs that were sufficiently informative for inference of tissue-migration histories from ones that were not. For this test, we compared an alternative model with a GTR tissue-migration model to a null model in which tissue labels were randomly sampled in proportion to their relative frequencies at the leaves of the tree. This test evaluates whether or not the tissue labels evolve in a Markovian manner along the branches of the tree—that is, whether or not each cell's tissue label depends on the label of its parent and the branch length between them. If such a dependency exists, then there is at least some information in the data about the tissue-migration process, whereas, if it does not exist, no such information is present. When we applied this test to our simulated data, we found that it was minimally restrictive, allowing all but a few datasets to pass at a threshold of log Bayes factor (lBf) >1.1 (Figure S14). Similarly, the test identified ∼98% of the lung-cancer CPs as informative about tissue migration. When we applied it to the prostate-cancer dataset, however, we found that only four CPs (∼1%) passed (Figure 4A; Table S2), indicating that the limited mutational content in this dataset permits only a minority of CPs to be informative about migration history. These results aligned with a simpler measure of mutual information derived from the full posterior distributions (Figure S15). Similarly, only three pancreatic and two additional lung-cancer clones passed this test (Figure S16).

We then focused our hypothesis testing of PR on the four prostate-cancer CPs that were most informative about migration history. In this case, we tested an alternative model allowing for PR against a null model in which PR events were forced to have rates of zero. We found positive support for PR in only one of the four CPs (Figure 4B), which, interestingly, came from a different mouse than the single case of PR previously identified using MACHINA.[37] As validation, we performed the same hypothesis test on simulated datasets with and without reseeding events, and we found that all simulations with support for PR did indeed exhibit PR, while 75% of the simulations supporting the null hypothesis did not exhibit PR (Figure S17). Among the simulations without positive support in either direction, 50% exhibited PR, suggesting that our test is conservative. Thus, our Bayes-factor analysis does support PR in the prostate-cancer model, albeit in a small fraction of CPs. Notably, the Bayesian hypothesis test is considerably more stringent than simply applying a posterior-probability threshold to the edges of the migration graph, as in the previous section.

For a second illustration, we designed a hypothesis test to distinguish between the two main patterns we observed in lung

cancer. We tested an alternative hypothesis in which LL → RL events were allowed (corresponding to the blue bars in Figure 3E) against a null hypothesis in which they were not, in which case we presume the data will be explained by the M-hub model (orange bars in Figure 3E). This test compared a fully parameterized model (representing the alternative hypothesis) against a model forced to have a rate of zero for LL → RL events (representing the null hypothesis). We found that the alternative hypothesis of direct LL → RL seeding obtained positive support in ∼40% of eligible CPs, whereas the null hypothesis was positively supported in ∼28% of CPs (Figure 4C). The remaining ∼32% supported neither model. This analysis further supports the finding that both the LL → RL and M-hub patterns are present in the data. It additionally suggests that somewhat more CPs exhibit the LL → RL than the M-hub-only pathway.

DISCUSSION

In this article, we introduced BEAM, a fully Bayesian framework that jointly models cell-lineage and tissue-migration histories to reconstruct the timing and routes of metastatic progression from single-cell lineage-tracing data. To evaluate BEAM, we developed a data simulator that integrates an agent-based model of cancer metastasis with CRISPR-based lineage recording in DNA barcodes. We found that BEAM consistently recovered simulated migration histories with higher precision and recall than existing methods and that the Bayesian approach uniquely allowed for control over the confidence associated with each migration event. In real data for lung and prostate cancer, BEAM uncovered complex and highly connected migration graphs even under stringent posterior-probability thresholds. In lung cancer, we identified two distinct patterns of metastatic progression driven by early divergence and found that liver metastases were preferentially seeded by secondary passage from a lymphatic tumor. In prostate cancer, BEAM highlighted diffuse posterior distributions over migration histories, which we traced to phylogenetic uncertainty resulting from limited mutational information. In both cases, BEAM effectively distinguished between signal and noise, preserving meaningful structure when data were informative while avoiding overfitting when they were not. We also showed that BEAM enabled direct hypothesis testing of features of the migration model, using it to assess the informativeness of lineage data, test for metastatic-to-PR events, and classify progression patterns.

Overall, BEAM makes three contributions to migration-history-inference methods. First, it simultaneously addresses the intertwined problems of lineage-tree and migration-graph inference, enabling lineage trees to be evaluated in part by the likelihood of their induced migration graphs. Second, BEAM samples from a full posterior distribution over migration graphs, which supports decision-making based on posterior-probability thresholds and avoids overconfidence in cases with weak data. We recommend edgewise posterior-probability thresholds of 0.50 for exploratory summaries and 0.90 for conservative results, corresponding to points shown on the plots for simulated data. Third, BEAM supports formal hypothesis testing by marginalizing over tree topologies and phylogenetic parameters, allowing rigorous evaluation of questions about migration structure, data informativeness, and specific evolutionary events. Notably, BEAM is also modular by design, making it adaptable to new lineage-tracing technologies as they evolve. Together, these features make BEAM a powerful framework for extracting meaningful insights from complex single-cell lineage-tracing data.

In our reanalysis of available lineage-tracing datasets, sparse mutation information was a persistent barrier to robust inference of tissue-migration histories (see also Salvador-Martínez et al., Jiang et al., and Gao and Feder[59–61]). This limitation was especially notable with the prostate-cancer dataset,[37] which derived from a system designed to maximize biological realism at some expense to mutation rates. Nevertheless, we found that even more mutation-rich datasets for lung[34,35] and pancreatic[44] cancer compared unfavorably with simulated data regimes in which migration graphs could be reconstructed accurately. We conducted extensive simulations spanning a range of mutation and migration rates, allowing readers to assess where future datasets may fall within these regimes. Developing improved barcoding techniques is a highly active area of research[62–66] (see Askary et al.[67] for a recent review), and we expect that the problem of sparse information will fade in importance as experimental methods improve. At present, however, it is critical for investigators to ensure that reconstructed migration histories are well supported by the data before drawing strong biological conclusions from them. To our knowledge, the Bayes-factor-based test proposed here is the first formal statistical test for this purpose, and we anticipate that it and tests like it will be important in ensuring that migration-graph inference is well grounded in the available data.

An important distinction between BEAM and multi-criteria parsimony approaches is that BEAM does not distinguish single-cell migration events from multiple-cell co-migrations. El-Kebir et al.[45] introduced the idea of explicitly modeling co-migrations to address the problem that polyclonal events can cause the number of migrations to be over-estimated by standard methods. Their co-migration-aware formulation of the parsimony problem, however, is challenging to solve and led them to employ a computationally intensive mixed integer linear programming approach (see also Koyyalagunta et al. and Roddur et al.[50,51]). In principle, it would be possible to model co-migrations in BEAM, but a good deal of additional complexity would be required. Instead, we chose simply to model all migration events as occurring independently. Interestingly, even though co-migrations were frequent in our simulations, BEAM performed well in recovering the true migration histories, suggesting that explicit modeling of co-migrations might not be necessary, provided a model is sufficiently flexible in other respects. Despite these encouraging results, future work could explore an extension of the Bayesian approach that models co-migrations. One interesting application would be to evaluate the degree to which the data support the hypothesis of co-migration.

Modeling co-migrations is closely tied to a debate in the field surrounding polyclonal vs. monoclonal metastatic seeding. Early studies favored monoclonal seeding,[68,69] whereas recent studies supported polyclonal seeding, with clones disseminating simultaneously or sequentially.[28,31,45,70–74] Nevertheless, polyclonal seeding does not always dominate.[70,75] For example, a recent genomic study uncovered homogeneous, monoclonal

CellPress
OPEN ACCESS

metastatic sites for several cancer types.[42] However, experimental sampling methods can introduce classification bias.[76] Metastasis stage and therapy also influence observations,[28,30,32,70,75,77–79] with distant or post-therapy metastases favoring monoclonal origins.[30,78,80] In cell-lineage tracing, both monoclonal and polyclonal origins were observed even when inferred using criteria that favored co-migrations.[81] Evidently, both tissue-seeding modes occur across disease settings, calling for flexible modeling strategies that can accommodate both.

Another major source of computational complexity in methods that infer a migration graph by parsimony based on a given lineage tree is the presence in the starting tree of polytomies, or nodes with more than two children. Polytomies reflect uncertainty about the structure of the tree and are common in trees estimated from lineage-tracing data when mutations are sparse. To find parsimonious migration graphs, these methods have to consider the set of all possible refinements of polytomy-containing trees into binary trees, a set that grows exponentially with the number of polytomies. In practice, trees with many polytomies, such as those from the prostate-cancer dataset we analyzed, can lead to extremely long running times. BEAM circumvents this problem by directly exploring the space of tissue-labeled binary lineage trees and considering their likelihoods under both the barcode-mutation and tissue-migration models. As a result, it can avoid regions of the solution space where the lineage tree fits the mutation data but implies an unlikely migration graph, improving efficiency.

Despite these advantages, BEAM has similar limits in scalability to the other available migration-history-inference methods. On one hand, BEAM benefits from simultaneous consideration of lineage trees and migration graphs, native handling of binary trees, a simple model of migration, and relatively fast likelihood calculations. On the other, its reliance on exploration of the full space of tissue-labeled lineage trees by MCMC prohibits it from scaling to thousands of cells. BEAM's precise scaling limits depended on several factors, including tree size, mutation data, and observed tissues, but we found that it could typically resolve lineage trees with up to 300 cells and was generally impractical to apply beyond that scale (Figure S18). In our hands, these limits were broadly comparable to those of the parsimony-based approaches MACHINA and Metient and higher than that of PathFinder, despite major differences between algorithmic strategies, suggesting current limits of the field that will require new innovations for major improvements in scalability (see Schmidt and Raphael[81] for one recent example). We note, however, that Metient has been optimized to use GPU resources for improved scalability but that we did not use them in this study. Building on our recent work replacing MCMC with variational inference in Bayesian phylogenetics,[82] we plan to extend this approach to tissue-migration modeling, as in BEAM, to improve scalability.

Although BEAM could, in principle, be extended to analyze bulk human tumor sequencing data for reconstructing metastatic seeding histories, doing so requires integrating allele-frequency-based mixture models of clonal evolution rather than the discrete sequence-based substitution models currently employed. Developing such extensions represents a substantial methodological shift beyond the scope of the present study.

Beyond modeling tissue migration, the Bayesian approach here is applicable to any discrete-state process evolving along a lineage tree, including cell-state transitions. In principle, BEAM could be adapted to infer cell-state hierarchies from lineage-tracing data, as in recent work.[35,83–86] However, such applications require extensions to model unobserved intermediate states that arise during differentiation, which are not explicitly represented in the current framework.

CRISPR-Cas9-based lineage-tracing technology is only a decade old, and its application to metastasis is even more recent. Related computational methods remain in their infancy, with considerable room for improvement. Nevertheless, BEAM represents an important step forward by introducing simultaneous Bayesian inference of lineage phylogenies and tissue-migration graphs, as well as Bayesian hypothesis testing, to the field. Because uncertainty about the true structure of the lineage tree and migration graph tends to be high, the Bayesian approach is particularly powerful. We expect the ideas introduced here to encourage continued methods development and to improve our understanding of metastasis.

Limitations of the study

Although we developed a simulation framework integrating an agent-based model of metastasis with CRISPR-based lineage tracing, it was designed using existing tools to benchmark BEAM across broad-parameter regimes rather than to exhaustively explore all biologically realistic seeding scenarios. In particular, polyclonal seeding was treated in a simplified manner, as we did not systematically vary the number of clones or their relative contributions to individual metastatic sites. While BEAM performed robustly under the diverse conditions examined, developing a more flexible, *de novo* simulation framework remains an important direction for future work.

RESOURCE AVAILABILITY

Lead contact

Requests for further information and resources will be fulfilled by the lead contact, Adam Siepel (asiepel@cshl.edu).

Materials availability

This study did not generate new materials.

Data and code availability

- The datasets reanalyzed in this study were found in the original reports.[34,35,37,44] All other data will be shared by the lead contact upon request.
- All code was archived on Zenodo: https://doi.org/10.5281/zenodo.18744445. Up-to-date versions are available on GitHub at https://github.com/CshlSiepelLab/beam, https://github.com/CshlSiepelLab/graphposterior, and https://github.com/CshlSiepelLab/beam_experiments.
- Additional information is available from the lead contact upon request.

ACKNOWLEDGMENTS

This work was performed with assistance from US National Institutes of Health (NIH) National Cancer Institute (NCI) grants R01-CA272466 (to D.G.N.) and 5P30CA045508 (to David Tuveson of CSHL), NIH National Institute of General Medical Sciences grant R35-GM127070 (to A.S.), Starr Cancer Consortium grant I16-0060 (to D.G.N.), an American Cancer Society Research Scholar

Grant (to D.G.N.), a Weill Cornell Medicine Walter B. Wriston Research Scholar Award (to D.G.N.), Department of Defense Prostate Cancer Research Program Early Investigator Research Award W81XWH-22-1-0068 (to R.N.S.), NIH/NCI Cancer Pharmacology Training Grant CA062948 (to R.N.S.), a National Science Foundation Graduate Research Fellowship (to S.J.S.), a Starr Centennial Scholarship from the Starr Foundation (to S.J.S.), and the Simons Center for Quantitative Biology at CSHL. The content is solely the responsibility of the authors and does not necessarily represent the official views of the NIH. We thank David M. McCandlish, Bruce Stillman, Hannah V. Meyer, and members of the Siepel and Nowak labs for support. We thank Mrinmoy S. Roddur for assistance with MACH2 and Divya Koyyalagunta for assistance with Metient.

AUTHOR CONTRIBUTIONS

Conceptualization, S.J.S., A. Scheben, D.G.N., and A. Siepel; methodology, S.J.S., A. Scheben, R.H., and A. Siepel; investigation, S.J.S., A. Scheben, L.M.B., R.H., R.N.S., J.X., D.G.N., and A. Siepel; writing – original draft, S.J.S. and A. Siepel; writing – review & editing, S.J.S., A. Scheben, L.M.B., R.H., R.N.S., J.X., D.G.N., and A. Siepel; funding acquisition, S.J.S., R.N.S., D.G.N., and A. Siepel; resources, D.G.N. and A. Siepel; supervision, A. Scheben, D.G.N., and A. Siepel.

DECLARATION OF INTERESTS

The authors declare no competing interests.

DECLARATION OF GENERATIVE AI AND AI-ASSISTED TECHNOLOGIES IN THE WRITING PROCESS

ChatGPT and Claude were used to assist in software implementation and manuscript preparation. The authors edited the content as needed and take full responsibility for the publication.

STAR★METHODS

Detailed methods are provided in the online version of this paper and include the following:

- KEY RESOURCES TABLE
- METHOD DETAILS
  - Modeling barcode mutation and tissue migration as conditionally independent processes
  - BEAM parameters and priors
  - Simulation model for cancer evolution and CRISPR lineage tracing
  - Simulated data
  - Alternative methods for benchmarking
  - Precision, recall, and F1 score calculations
  - Calculating excess migrations relative to parsimony
  - Application to lung, prostate, and pancreatic cancer datasets
  - Previously reported levels of M2M and PR for real data
  - Hypothesis testing with Bayes factors
  - Calculating mutual information of tissue transitions
  - Evaluating the PR test on simulated data
- QUANTIFICATION AND STATISTICAL ANALYSIS

SUPPLEMENTAL INFORMATION

Supplemental information can be found online at https://doi.org/10.1016/j.xgen.2026.101193.

Received: September 16, 2025
Revised: January 7, 2026
Accepted: March 3, 2026
Published: March 30, 2026

REFERENCES

1. Nowell, P.C. (1976). The clonal evolution of tumor cell populations. Science *194*, 23–28. https://doi.org/10.1126/science.959840.
2. Felsenstein, J. (1981). Evolutionary trees from DNA sequences: A maximum likelihood approach. J. Mol. Evol. *17*, 368–376. https://doi.org/10.1007/bf01734359.
3. Felsenstein, J. (2004). Inferring Phylogenies (Sinauer Associates).
4. Yang, Z. (2014). Molecular Evolution: A Statistical Approach (Oxford University Press).
5. Beerenwinkel, N., Schwarz, R.F., Gerstung, M., and Markowetz, F. (2015). Cancer evolution: mathematical models and computational inference. Syst. Biol. *64*, e1–e25. https://doi.org/10.1093/sysbio/syu081.
6. Graham, T.A., and Sottoriva, A. (2017). Measuring cancer evolution from the genome. J. Pathol. *241*, 183–191. https://doi.org/10.1002/path.4821.
7. Junker, J.P., Spanjaard, B., Peterson-Maduro, J., Alemany, A., Hu, B., Florescu, M., and van Oudenaarden, A. (2017). Massively parallel clonal analysis using CRISPR/Cas9 induced genetic scars. bioRxiv. https://doi.org/10.1101/056499.
8. Li, L., Xie, W., Zhan, L., Wen, S., Luo, X., Xu, S., Cai, Y., Tang, W., Wang, Q., Li, M., et al. (2024). Resolving tumor evolution: a phylogenetic approach. J Nat Cancer Cent *4*, 97–106. https://doi.org/10.1016/j.jncc.2024.03.001.
9. McKenna, A., Findlay, G.M., Gagnon, J.A., Horwitz, M.S., Schier, A.F., and Shendure, J. (2016). Whole-organism lineage tracing by combinatorial and cumulative genome editing. Science *353*, aaf7907. https://doi.org/10.1126/science.aaf7907.
10. Spanjaard, B., Hu, B., Mitic, N., Olivares-Chauvet, P., Janjuha, S., Ninov, N., and Junker, J.P. (2018). Simultaneous lineage tracing and cell-type identification using CRISPR-Cas9-induced genetic scars. Nat Biotech *36*, 469–473. https://doi.org/10.1038/nbt.4124.
11. Frieda, K.L., Linton, J.M., Hormoz, S., Choi, J., Chow, K.H.K., Singer, Z.S., Budde, M.W., Elowitz, M.B., and Cai, L. (2017). Synthetic recording and in situ readout of lineage information in single cells. Nature *541*, 107–111. https://doi.org/10.1038/nature20777.
12. McKenna, A., and Gagnon, J.A. (2019). Recording development with single cell dynamic lineage tracing. Development *146*, dev169730. https://doi.org/10.1242/dev.169730.
13. Kuipers, J., Jahn, K., and Beerenwinkel, N. (2017). Advances in understanding tumour evolution through single-cell sequencing. Biochim. Biophys. Acta *1867*, 127–138. https://doi.org/10.1016/j.bbcan.2017.02.001.
14. Jones, M.G., Khodaverdian, A., Quinn, J.J., Chan, M.M., Hussmann, J.A., Wang, R., Xu, C., Weissman, J.S., and Yosef, N. (2020). Inference of single-cell phylogenies from lineage tracing data using Cassiopeia. Genome Biol. *21*, 92. https://doi.org/10.1186/s13059-020-02000-8.
15. Feng, J., Dewitt, W.S., McKenna, A., Simon, N., Willis, A.D., and Matsen, F.A. (2021). Estimation of cell lineage trees by maximum-likelihood phylogenetics. Ann. Appl. Stat. *15*, 343–362. https://doi.org/10.1214/20-aoas1400.
16. Gong, W., Kim, H.J., Garry, D.J., and Kwak, I.Y. (2022). Single cell lineage reconstruction using distance-based algorithms and the R package. BMC Bioinf. *23*, 103. https://doi.org/10.1186/s12859-022-04633-x.
17. Prusokiene, A., Prusokas, A., and Retkute, R. (2023). Machine learning based lineage tree reconstruction improved with knowledge of higher level relationships between cells and genomic barcodes. NAR Genom. Bioinform. *5*, lqad077. https://doi.org/10.1093/nargab/lqad077.
18. Sashittal, P., Schmidt, H., Chan, M., and Raphael, B.J. (2023). Startle: A star homoplasy approach for CRISPR-Cas9 lineage tracing. Cell Syst. *14*, 1113–1121.e9. https://doi.org/10.1016/j.cels.2023.11.005.
19. Chu, G., Mai, U., Schmidt, H., and Raphael, B.J. (2025). Maximum likelihood inference of time-scaled cell lineage trees with mixed-type missing data using LAML. Genome Biol. *26*, 189. https://doi.org/10.1186/s13059-025-03649-9.

CellPress
OPEN ACCESS

20. Alemany, A., Florescu, M., Baron, C.S., Peterson-Maduro, J., and van Oudenaarden, A. (2018). Whole-organism clone tracing using single-cell sequencing. Nature *556*, 108–112. https://doi.org/10.1038/nature25969.

21. Chan, M.M., Smith, Z.D., Grosswendt, S., Kretzmer, H., Norman, T.M., Adamson, B., Jost, M., Quinn, J.J., Yang, D., Jones, M.G., et al. (2019). Molecular recording of mammalian embryogenesis. Nature *570*, 77–82. https://doi.org/10.1038/s41586-019-1184-5.

22. Gong, W., Granados, A.A., Hu, J., Jones, M.G., Raz, O., Salvador-Martínez, I., Zhang, H., Chow, K.H.K., Kwak, I.Y., Retkute, R., et al. (2021). Benchmarked approaches for reconstruction of in vitro cell lineages and in silico models of C. elegans and M. musculus developmental trees. Cell Syst. *12*, 810–826.e4. https://doi.org/10.1016/j.cels.2021.05.008.

23. Xie, L., Liu, H., You, Z., Wang, L., Li, Y., Zhang, X., Ji, X., He, H., Yuan, T., Zheng, W., et al. (2023). Comprehensive spatiotemporal mapping of single-cell lineages in developing mouse brain by CRISPR-based barcoding. Nat. Methods *20*, 1244–1255. https://doi.org/10.1038/s41592-023-01947-3.

24. Ganesh, K., and Massagué, J. (2021). Targeting metastatic cancer. Nat. Med. *27*, 34–44. https://doi.org/10.1038/s41591-020-01195-4.

25. Siegel, R.L., Kratzer, T.B., Giaquinto, A.N., Sung, H., and Jemal, A. (2025). Cancer statistics, 2025. CA: A. Cancer J Clin *75*, 10–45. https://doi.org/10.3322/caac.21871.

26. Dillekås, H., Rogers, M.S., and Straume, O. (2019). Are 90% of deaths from cancer caused by metastases? Cancer Med. *8*, 5574–5576. https://doi.org/10.1002/cam4.2474.

27. Boire, A., Burke, K., Cox, T.R., Guise, T., Jamal-Hanjani, M., Janowitz, T., Kaplan, R., Lee, R., Swanton, C., Vander Heiden, M.G., and Sahai, E. (2024). Why do patients with cancer die? Nat. Rev. Cancer *24*, 578–589. https://doi.org/10.1038/s41568-024-00708-4.

28. Gundem, G., Van Loo, P., Kremeyer, B., Alexandrov, L.B., Tubio, J.M.C., Papaemmanuil, E., Brewer, D.S., Kallio, H.M.L., Högnäs, G., Annala, M., et al. (2015). The evolutionary history of lethal metastatic prostate cancer. Nature *520*, 353–357. https://doi.org/10.1038/nature14347.

29. Wei, Q., Ye, Z., Zhong, X., Li, L., Wang, C., Myers, R.E., Palazzo, J.P., Fortuna, D., Yan, A., Waldman, S.A., et al. (2017). Multiregion whole-exome sequencing of matched primary and metastatic tumors revealed genomic heterogeneity and suggested polyclonal seeding in colorectal cancer metastasis. Ann. Oncol. *28*, 2135–2141. https://doi.org/10.1093/annonc/mdx278.

30. Hu, Z., Li, Z., Ma, Z., and Curtis, C. (2020). Multi-cancer analysis of clonality and the timing of systemic spread in paired primary tumors and metastases. Nat. Genet. *52*, 701–708. https://doi.org/10.1038/s41588-020-0628-z.

31. Sanborn, J.Z., Chung, J., Purdom, E., Wang, N.J., Kakavand, H., Wilmott, J.S., Butler, T., Thompson, J.F., Mann, G.J., Haydu, L.E., et al. (2015). Phylogenetic analyses of melanoma reveal complex patterns of metastatic dissemination. Proc. Natl. Acad. Sci. USA *112*, 10995–11000. https://doi.org/10.1073/pnas.1508074112.

32. McPherson, A., Roth, A., Laks, E., Masud, T., Bashashati, A., Zhang, A.W., Ha, G., Biele, J., Yap, D., Wan, A., et al. (2016). Divergent modes of clonal spread and intraperitoneal mixing in high-grade serous ovarian cancer. Nat. Genet. *48*, 758–767. https://doi.org/10.1038/ng.3573.

33. Turajlic, S., Xu, H., Litchfield, K., Rowan, A., Chambers, T., Lopez, J.I., Nicol, D., O'Brien, T., Larkin, J., Horswell, S., et al. (2018). Tracking cancer evolution reveals constrained routes to metastases: TRACERx Renal. Cell *173*, 581–594.e12. https://doi.org/10.1016/j.cell.2018.03.057.

34. Quinn, J.J., Jones, M.G., Okimoto, R.A., Nanjo, S., Chan, M.M., Yosef, N., Bivona, T.G., and Weissman, J.S. (2021). Single-cell lineages reveal the rates, routes, and drivers of metastasis in cancer xenografts. Science *371*, eabc1944. https://doi.org/10.1126/science.abc1944.

35. Yang, D., Jones, M.G., Naranjo, S., Rideout, W.M., Min, K.H.J., Ho, R., Wu, W., Replogle, J.M., Page, J.L., Quinn, J.J., et al. (2022). Lineage tracing reveals the phylodynamics, plasticity, and paths of tumor evolution. Cell *185*, 1905–1923.e25. https://doi.org/10.1016/j.cell.2022.04.015.

36. Hessey, S., Fessas, P., Zaccaria, S., Jamal-Hanjani, M., and Swanton, C. (2023). Insights into the metastatic cascade through research autopsies. Trends Cancer *9*, 490–502. https://doi.org/10.1016/j.trecan.2023.03.002.

37. Serio, R.N., Scheben, A., Lu, B., Gargiulo, D.V., Patruno, L., Buckholtz, C.L., Chaffee, R.J., Jibilian, M.C., Persaud, S.G., Staklinski, S.J., et al. (2024). Clonal lineage tracing with somatic delivery of recordable barcodes reveals migration histories of metastatic prostate cancer. Cancer Discov. *14*, 1990–2009. https://doi.org/10.1158/2159-8290.CD-23-1332.

38. Sleeman, J.P. (2000). The lymph node as a bridgehead in the metastatic dissemination of tumors. Recent Results Cancer Res. *157*, 55–81. https://doi.org/10.1007/978-3-642-57151-0_6.

39. Disibio, G., and French, S.W. (2008). Metastatic patterns of cancers: results from a large autopsy study. Arch. Pathol. Lab Med. *132*, 931–939. https://doi.org/10.5858/2008-132-931-MPOCRF.

40. Dunbar, K.J., Efe, G., Cunningham, K., Esquea, E., Navaridas, R., and Rustgi, A.K. (2025). Regulation of metastatic organotropism. Trends Cancer *11*, 216–231. https://doi.org/10.1016/j.trecan.2024.11.012.

41. Gao, Y., Bado, I., Wang, H., Zhang, W., Rosen, J.M., and Zhang, X.H.F. (2019). Metastasis organotropism: Redefining the congenial soil. Dev. Cell *49*, 375–391. https://doi.org/10.1016/j.devcel.2019.04.012.

42. Nguyen, B., Fong, C., Luthra, A., Smith, S.A., DiNatale, R.G., Nandakumar, S., Walch, H., Chatila, W.K., Madupuri, R., Kundra, R., et al. (2022). Genomic characterization of metastatic patterns from prospective clinical sequencing of 25,000 patients. Cell *185*, 563–575.e11. https://doi.org/10.1016/j.cell.2022.01.003.

43. Paget, S. (1889). The distribution of secondary growths in cancer of the breast. Lancet *133*, 571–573. https://doi.org/10.1016/S0140-6736(00)49915-0.

44. Simeonov, K.P., Byrns, C.N., Clark, M.L., Norgard, R.J., Martin, B., Stanger, B.Z., Shendure, J., McKenna, A., and Lengner, C.J. (2021). Single-cell lineage tracing of metastatic cancer reveals selection of hybrid EMT states. Cancer Cell *39*, 1150–1162.e9. https://doi.org/10.1016/j.ccell.2021.05.005.

45. El-Kebir, M., Satas, G., and Raphael, B.J. (2018). Inferring parsimonious migration histories for metastatic cancers. Nat. Genet. *50*, 718–726. https://doi.org/10.1038/s41588-018-0106-z.

46. Chroni, A., Vu, T., Miura, S., and Kumar, S. (2019). Delineation of tumor migration paths by using a Bayesian biogeographic approach. Cancers *11*, 1880. https://doi.org/10.3390/cancers11121880.

47. Chroni, A., and Kumar, S. (2021). Tumors are evolutionary island-like ecosystems. Genome Biol. Evol. *13*, evab276. https://doi.org/10.1093/gbe/evab276.

48. Chroni, A., Miura, S., Oladeinde, O., Aly, V., and Kumar, S. (2021). Migrations of cancer cells through the lens of phylogenetic biogeography. Sci. Rep. *11*, 17184. https://doi.org/10.1038/s41598-021-96215-9.

49. Kumar, S., Chroni, A., Tamura, K., Sanderford, M., Oladeinde, O., Aly, V., Vu, T., and Miura, S. (2020). Pathfinder: Bayesian inference of clone migration histories in cancer. Bioinformatics *36*, i675–i683. https://doi.org/10.1093/bioinformatics/btaa795.

50. Koyyalagunta, D., Ganesh, K., and Morris, Q. (2025). Inferring cancer type-specific patterns of metastatic spread using metient. Nat. Methods *23*, 574–584. https://doi.org/10.1038/s41592-025-02924-8.

51. Roddur, M.S., Ramavarapu, V., Bunkum, A., Huebner, A., Mineyev, R., McGranahan, N., Zaccaria, S., and El-Kebir, M. (2024). Characterizing the solution space of migration histories of metastatic cancers with MACH2. bioRxiv. https://doi.org/10.1101/2024.11.19.624301.

52. Bouckaert, R., Vaughan, T.G., Barido-Sottani, J., Duchêne, S., Fourment, M., Gavryushkina, A., Heled, J., Jones, G., Kühnert, D., De Maio, N., et al. (2019). BEAST 2.5: An advanced software platform for Bayesian evolutionary analysis. PLoS Comput. Biol. *15*, e1006650. https://doi.org/10.1371/journal.pcbi.1006650.

53. Seidel, S., and Stadler, T. (2022). TiDeTree: a Bayesian phylogenetic framework to estimate single-cell trees and population dynamic parameters from genetic lineage tracing data. Proc. Biol. Sci. *289*, 20221844. https://doi.org/10.1098/rspb.2022.1844.

54. Felsenstein, J. (1973). Maximum likelihood and minimum-steps methods for estimating evolutionary trees from data on discrete characters. Syst. Biol. *22*, 240–249. https://doi.org/10.1093/sysbio/22.3.240.

55. Reiter, J.G., Bozic, I., Chatterjee, K., and Nowak, M.A. (2013). Ttp: Tool for tumor progression. arXiv. http://arxiv.org/abs/1303.5251.

56. Fitch, W.M. (1971). Toward defining the course of evolution: Minimum change for a specific tree topology. Syst. Zool. *20*, 406–416. https://doi.org/10.2307/2412116.

57. Hartigan, J.A. (1973). Minimum mutation fits to a given tree. Biometrics *29*, 53–65. https://doi.org/10.2307/2529676.

58. Miura, S., Vu, T., Choi, J., Townsend, J.P., Karim, S., and Kumar, S. (2022). A phylogenetic approach to study the evolution of somatic mutational processes in cancer. Commun. Biol. *5*, 617. https://doi.org/10.1038/s42003-022-03560-0.

59. Salvador-Martínez, I., Grillo, M., Averof, M., and Telford, M.J. (2019). Is it possible to reconstruct an accurate cell lineage using CRISPR recorders? eLife *8*, e40292. https://doi.org/10.7554/elife.40292.

60. Jiang, J., Ye, X., Kong, Y., Guo, C., Zhang, M., Cao, F., Zhang, Y., and Pei, W. (2025). scLTdb: a comprehensive single-cell lineage tracing database. Nucleic Acids Res. *53*, D1173–D1185. https://doi.org/10.1093/nar/gkae913.

61. Gao, Y., and Feder, A.F. (2025). Detecting branching rate heterogeneity with tree balance statistics in lineage tracing trees. bioRxiv. https://doi.org/10.1101/2024.06.27.601073.

62. Choi, J., Chen, W., Minkina, A., Chardon, F.M., Suiter, C.C., Regalado, S.G., Domcke, S., Hamazaki, N., Lee, C., Martin, B., et al. (2022). A time-resolved, multi-symbol molecular recorder via sequential genome editing. Nature *608*, 98–107. https://doi.org/10.1038/s41586-022-04922-8.

63. Chadly, D.M., Frieda, K.L., Gui, C., Klock, L., Tran, M., Sui, M.Y., Takei, Y., Bouckaert, R., Lois, C., Cai, L., and Elowitz, M.B. (2024). Reconstructing cell histories in space with image-readable base editor recording. bioRxiv. https://doi.org/10.1101/2024.01.03.573434.

64. Koblan, L.W., Yost, K.E., Zheng, P., Colgan, W.N., Jones, M.G., Yang, D., Kumar, A., Sandhu, J., Schnell, A., Sun, D., et al. (2025). High-resolution spatial mapping of cell state and lineage dynamics in vivo with PEtracer. Science *390*, eadx3800. https://doi.org/10.1126/science.adx3800.

65. Chen, C., Liao, Y., Zhu, M., Wang, L., Yu, X., Li, M., and Peng, G. (2025). Dual-nuclease single-cell lineage tracing by Cas9 and Cas12a. Cell Rep. *44*, 115105. https://doi.org/10.1016/j.celrep.2024.115105.

66. Winter, E., Emiliani, F., Cook, A., Abderrahim, A., and McKenna, A. (2025). BASELINE: A CRISPR base editing platform for mammalian-scale single-cell lineage tracing. bioRxiv. https://doi.org/10.1101/2025.03.19.644238.

67. Askary, A., Chen, W., Choi, J., Du, L.Y., Elowitz, M.B., Gagnon, J.A., Schier, A.F., Seidel, S., Shendure, J., Stadler, T., and Tran, M. (2025). The lives of cells, recorded. Nat. Rev. Genet. *26*, 203–222. https://doi.org/10.1038/s41576-024-00788-w.

68. Yamamoto, N., Yang, M., Jiang, P., Xu, M., Tsuchiya, H., Tomita, K., Moossa, A.R., and Hoffman, R.M. (2003). Determination of clonality of metastasis by cell-specific color-coded fluorescent-protein imaging. Cancer Res. 63, 7785–7790.

69. Liu, W., Laitinen, S., Khan, S., Vihinen, M., Kowalski, J., Yu, G., Chen, L., Ewing, C.M., Eisenberger, M.A., Carducci, M.A., et al. (2009). Copy number analysis indicates monoclonal origin of lethal metastatic prostate cancer. Nat. Med. *15*, 559–565. https://doi.org/10.1038/nm.1944.

70. Maddipati, R., and Stanger, B.Z. (2015). Pancreatic Cancer Metastases Harbor Evidence of Polyclonality. Cancer Discov. *5*, 1086–1097. https://doi.org/10.1158/2159-8290.CD-15-0120.

71. Cheung, K.J., Padmanaban, V., Silvestri, V., Schipper, K., Cohen, J.D., Fairchild, A.N., Gorin, M.A., Verdone, J.E., Pienta, K.J., Bader, J.S., and Ewald, A.J. (2016). Polyclonal breast cancer metastases arise from collective dissemination of keratin 14-expressing tumor cell clusters. Proc. Natl. Acad. Sci. USA *113*, E854–E863. https://doi.org/10.1073/pnas.1508541113.

72. Hoadley, K.A., Siegel, M.B., Kanchi, K.L., Miller, C.A., Ding, L., Zhao, W., He, X., Parker, J.S., Wendl, M.C., Fulton, R.S., et al. (2016). Tumor Evolution in Two Patients with Basal-like Breast Cancer: A Retrospective Genomics Study of Multiple Metastases. PLoS Med. *13*, e1002174. https://doi.org/10.1371/journal.pmed.1002174.

73. Echeverria, G.V., Powell, E., Seth, S., Ge, Z., Carugo, A., Bristow, C., Peoples, M., Robinson, F., Qiu, H., Shao, J., et al. (2018). High-resolution clonal mapping of multi-organ metastasis in triple negative breast cancer. Nat. Commun. *9*, 5079. https://doi.org/10.1038/s41467-018-07406-4.

74. Heyde, A., Reiter, J.G., Naxerova, K., and Nowak, M.A. (2019). Consecutive seeding and transfer of genetic diversity in metastasis. Proc. Natl. Acad. Sci. USA *116*, 14129–14137. https://doi.org/10.1073/pnas.1819408116.

75. Woodcock, D.J., Riabchenko, E., Taavitsainen, S., Kankainen, M., Gundem, G., Brewer, D.S., Ellonen, P., Lepistö, M., Golubeva, Y.A., Warner, A.C., et al. (2020). Prostate cancer evolution from multilineage primary to single lineage metastases with implications for liquid biopsy. Nat. Commun. *11*, 5070. https://doi.org/10.1038/s41467-020-18843-5.

76. Birkbak, N.J., and McGranahan, N. (2020). Cancer genome evolutionary trajectories in metastasis. Cancer Cell *37*, 8–19. https://doi.org/10.1016/j.ccell.2019.12.004.

77. Turajlic, S., Xu, H., Litchfield, K., Rowan, A., Horswell, S., Chambers, T., O'Brien, T., Lopez, J.I., Watkins, T.B.K., Nicol, D., et al. (2018). Deterministic evolutionary trajectories influence primary tumor growth: TRACERx Renal. Cell *173*, 595–610.e11. https://doi.org/10.1016/j.cell.2018.03.043.

78. Hu, Z., Ding, J., Ma, Z., Sun, R., Seoane, J.A., Scott Shaffer, J., Suarez, C.J., Berghoff, A.S., Cremolini, C., Falcone, A., et al. (2019). Quantitative evidence for early metastatic seeding in colorectal cancer. Nat. Genet. *51*, 1113–1122. https://doi.org/10.1038/s41588-019-0423-x.

79. Tang, Y.J., Huang, J., Tsushima, H., Ban, G.I., Zhang, H., Oristian, K.M., Puviindran, V., Williams, N., Ding, X., Ou, J., et al. (2019). Tracing Tumor Evolution in Sarcoma Reveals Clonal Origin of Advanced Metastasis. Cell Rep. *28*, 2837–2850.e5. https://doi.org/10.1016/j.celrep.2019.08.029.

80. Reiter, J.G., Hung, W.T., Lee, I.H., Nagpal, S., Giunta, P., Degner, S., Liu, G., Wassenaar, E.C.E., Jeck, W.R., Taylor, M.S., et al. (2020). Lymph node metastases develop through a wider evolutionary bottleneck than distant metastases. Nat. Genet. *52*, 692–700. https://doi.org/10.1038/s41588-020-0633-2.

81. Schmidt, H., and Raphael, B.J. (2025). The tree labeling polytope: a unified approach to ancestral reconstruction problems. bioRxiv. https://doi.org/10.1101/2025.02.14.638328.

82. Siepel, A., Hassett, R., and Staklinski, S.J. (2025). Variational inference with node embeddings (VINE) for scalable Bayesian phylogenetics. bioRxiv. https://doi.org/10.64898/2025.12.24.696405.

83. Fang, W., Bell, C.M., Sapirstein, A., Asami, S., Leeper, K., Zack, D.J., Ji, H., and Kalhor, R. (2022). Quantitative fate mapping: A general framework for analyzing progenitor state dynamics via retrospective lineage barcoding. Cell *185*, 4604–4620.e32. https://doi.org/10.1016/j.cell.2022.10.028.

84. Schiffman, J.S., D'Avino, A.R., Prieto, T., Pang, Y., Fan, Y., Rajagopalan, S., Potenski, C., Hara, T., Suvà, M.L., Gawad, C., and Landau, D.A. (2024). Defining heritability, plasticity, and transition dynamics of cellular phenotypes in somatic evolution. Nat. Genet. *56*, 2174–2184. https://doi.org/10.1038/s41588-024-01920-6.

85. Sashittal, P., Zhang, R.Y., Law, B.K., Schmidt, H., Strzalkowski, A., Bolondi, A., Chan, M.M., and Raphael, B.J. (2025). Inferring cell differentiation maps from lineage tracing data. Nat. Methods *23*, 532–541. https://doi.org/10.1038/s41592-025-02903-z.

CellPress
OPEN ACCESS

86. Howard-Snyder, W., Zhang, R., Schmidt, H., Chan, M., and Raphael, B.J. (2025). Inferring cell differentiation dynamics with unobserved progenitors. bioRxiv. https://doi.org/10.64898/2025.12.09.693214.

87. Kuzmin, K., Schmidt, H., Kafi Kang, M., Snir, S., Raphael, B.J., and Skums, P. (2025). A graph homomorphism approach for unraveling histories of metastatic cancers and viral outbreaks under evolutionary constraints. Nat. Commun. *16*, 8027. https://doi.org/10.1038/s41467-025-63411-4.

88. Cho, H., Herzka, T., Zheng, W., Qi, J., Wilkinson, J.E., Bradner, J.E., Robinson, B.D., Castillo-Martin, M., Cordon-Cardo, C., and Trotman, L.C. (2014). RapidCaP, a novel GEM model for metastatic prostate cancer analysis and therapy, reveals Myc as a driver of Pten-mutant metastasis. Cancer Discov. *4*, 318–333. https://doi.org/10.1158/2159-8290.CD-13-0346.

89. Felsenstein, J. (1988). Phylogenies from molecular sequences: inference and reliability. Ann Rev Genet *22*, 521–565. https://doi.org/10.1146/annurev.ge.22.120188.002513.

90. Hey, J., and Nielsen, R. (2007). Integration within the Felsenstein equation for improved Markov chain Monte Carlo methods in population genetics. Proc. Natl. Acad. Sci. USA *104*, 2785–2790. https://doi.org/10.1073/pnas.0611164104.

91. Russel, P.M., Brewer, B.J., Klaere, S., and Bouckaert, R.R. (2019). Model selection and parameter inference in phylogenetics using nested sampling. Syst. Biol. *68*, 219–233. https://doi.org/10.1093/sysbio/syy050.

92. Skilling, J. (2006). Nested sampling for general Bayesian computation. Bayesian Anal. *1*, 833–859. https://doi.org/10.1214/06-BA127.

93. Kass, R.E., and Raftery, A.E. (1995). Bayes factors. J. Am. Stat. Assoc. *90*, 773–795.

94. Barido-Sottani, J., Bošková, V., Plessis, L.D., Kühnert, D., Magnus, C., Mitov, V., Müller, N.F., PečErska, J., Rasmussen, D.A., Zhang, C., et al. (2017). Taming the BEAST—a community teaching material resource for BEAST 2. Syst. Biol. *67*, 170–174. https://doi.org/10.1093/sysbio/syx060.

STAR★METHODS

KEY RESOURCES TABLE

REAGENT or RESOURCE	SOURCE	IDENTIFIER
**Deposited data**		
Processed data and raw scRNA-seq libraries of metastatic A549 cell lines	Quinn and Jones et al.[34]	Gene Expression Omnibus (GEO): GSE161363
Processed data for KP-Tracer tumors	Yang et al.[35]	Zenodo: https://doi.org/10.5281/zenodo.5847461
Processed data for KPCY pancreatic tumor lineage tracing	Simeonov et al.[44]	Mendeley data: https://doi.org/10.17632/t98pjcd7t6.1
Processed data for EvoCaP prostate cancer lineage tracing	Serio et al.[37]	Available upon request and Code Ocean: codeocean.com/capsule/5050757/tree/v2
**Software and algorithms**		
BEAM	This manuscript	Zenodo: https://doi.org/10.5281/zenodo.18744445 and GitHub: github.com/CshlSiepelLab/beam
graphposterior	This manuscript	Zenodo: https://doi.org/10.5281/zenodo.18744445 and GitHub: github.com/CshlSiepelLab/graphposterior
beam_experiments	This manuscript	Zenodo: https://doi.org/10.5281/zenodo.18744445 and GitHub: github.com/CshlSiepelLab/beam_experiments
Cassiopeia	Jones et al.[14]	GitHub: github.com/YosefLab/Cassiopeia
LAML	Chu and Mai et al.[19]	GitHub: github.com/raphael-group/LAML
MACHINA	El-Kebir et al.[45]	GitHub: github.com/raphael-group/machina
PathFinder	Miura et al.[58]	GitHub: github.com/SayakaMiura/PathFinder
Metient	Koyyalagunta et al.[50]	GitHub: github.com/morrislab/metient
MACH2	Roddur et al.[51]	GitHub: github.com/elkebir-group/MACH2

METHOD DETAILS

Modeling barcode mutation and tissue migration as conditionally independent processes

The barcode-mutation and tissue-migration processes were modeled as conditionally independent continuous-time Markov chains (CTMCs), given the phylogeny and branch lengths. Consider a single branch of the tree with length b, leading from a parent node u to a child node v. We represent the joint conditional probability of a barcode mutation state B_v and tissue label T_v at the child, given a barcode mutation state B_u and tissue label T_u at the parent, as a product of the two conditional probabilities,

$$P(T_v, B_v | T_u, B_u, b) = P(T_v | T_u, b)\, P(B_v | B_u, b).$$

As a result, the likelihood of observed barcode data $\mathbf{B}$ and tissue labels $\mathbf{T}$ at the tips of a tree with topology $\mathcal{T}$ and branch lengths $\mathbf{b}$ can be expressed as a product of phylogenetic likelihoods for the barcode and tissue labels, respectively,

$$\mathcal{L}(\theta, \mathcal{T}, \mathbf{b};\ \mathbf{B}, \mathbf{T}) = P(\mathbf{B}, \mathbf{T} | \theta_{\mathbf{B}}, \theta_{\mathbf{T}}, \mathcal{T}, \mathbf{b}) = P(\mathbf{B} | \theta_{\mathbf{B}}, \mathcal{T}, \mathbf{b})\, P(\mathbf{T} | \theta_{\mathbf{T}}, \mathcal{T}, \mathbf{b}),$$

where $\theta = \{\theta_{\mathbf{T}}, \theta_{\mathbf{B}}\}$ such that $\theta_{\mathbf{B}}$ contains parameters for the barcode mutation process and $\theta_{\mathbf{T}}$ for the tissue migration process. Each CTMC is defined by a corresponding rate matrix and an overall strict clock rate, as detailed below.

The barcode mutation model, adapted from TiDeTree[53] and similar to LAML,[19] was designed to describe an irreversible CRISPR-induced mutation process with the potential for silencing at individual sites. In particular, the model is defined by an infinitesimal generator Q_B, such that:

$$Q_B = \begin{bmatrix} -(1+l) & s_1 & \dots & s_N & l \\ 0 & -l & \dots & 0 & l \\ 0 & 0 & \dots & 0 & l \\ \vdots & \vdots & \vdots & \ddots & \vdots \\ 0 & 0 & \dots & -l & l \\ 0 & 0 & \dots & 0 & 0 \end{bmatrix}.$$

The first state represents the unmutated barcode, the last state represents a heritably silenced barcode, and the remaining N states represent unique indel (insertion or deletion) outcomes. Notice that all edits and silencing are assumed to be irreversible. Model

parameters include the silencing rate l and relative indel rates $\mathbf{s}_N$, where $s_i \in \mathbf{s}_N$ is scaled such that $\sum_{i=1}^{N} s_i = 1$. This scaling ensures that the expected editing rate is one and therefore that estimated branch lengths can be interpreted in units of expected indels per site. We omit the notion of a "scarring window" used in ref.[53] and allow mutations to occur at any time.

Tissue transitions were modeled with a separate infinitesimal generator Q_T, whose state space corresponds to the set of available tissues. Each off-diagonal entry q_{ij} in Q_T represents the instantaneous migration rate from tissue i to tissue j. By default, we assume a general time reversible (GTR) parameterization, where each entry is given by $q_{ij} = r_{ij}\pi_j$, with symmetric exchangeability rates $r_{ij} = r_{ji}$, and equilibrium tissue frequencies π_j such that $\sum_j \pi_j = 1$. The full rate matrix takes the form:

$$Q_T = \begin{bmatrix} -\sum_{j\neq 1} r_{1j}\pi_j & r_{12}\pi_2 & r_{13}\pi_3 & \cdots & r_{1n}\pi_n \\ r_{21}\pi_1 & -\sum_{j\neq 2} r_{2j}\pi_j & r_{23}\pi_3 & \cdots & r_{2n}\pi_n \\ r_{31}\pi_1 & r_{32}\pi_2 & -\sum_{j\neq 3} r_{3j}\pi_j & \cdots & r_{3n}\pi_n \\ \vdots & \vdots & \vdots & \ddots & \vdots \\ r_{n1}\pi_1 & r_{n2}\pi_2 & r_{n3}\pi_3 & \cdots & -\sum_{j\neq n} r_{nj}\pi_j \end{bmatrix},$$

where the diagonal terms ensure that each row sums to zero. The full rate matrix is normalized such that, under the tissue equilibrium frequencies, the expected number of transitions per unit time is equal to one. BEAM explicitly parameterizes the equilibrium frequency of the first tissue, π_1, which is assumed to be the primary source of the tumor, and sets the remaining frequencies to be equal:

$$\pi_j = \frac{1-\pi_1}{N-1} \quad \text{for } j \neq 1.$$

Likelihoods were computed using Felsenstein's pruning algorithm.[54] In the case of the barcode mutation model, for a leaf node i with observed barcode state B_i, the partial likelihood $L_i(x)$—indicating the probability of the data beneath node i given that node i has state x—is initialized as:

$$L_i(x) = \begin{cases} 1 & \text{if } x = B_i \\ 0 & \text{otherwise.} \end{cases}$$

In the case of the tissue migration model, $L_i(x)$ is analogously set to 1 if, and only if, x is equal to the tissue label at leaf i, T_i. In both cases, the recurrence relation for internal node j with children k and l is:

$$L_j(x) = \prod_{i\in\{k,l\}} \left(\sum_y L_i(y) P(x \to y | b_i) \right),$$

where $P(x \to y|b_i)$ represents the conditional probability of state y at child node $i \in \{k,l\}$ given state x at parent node j and branch length b_i. These conditional probabilities are obtained, in the usual way, by computing the matrix exponential $P_B(b_i) = exp(Q_B b_i)$ or $P_T(b_i) = exp(Q_T b_i)$.[4]

At the root node r, the final likelihood is given by:

$$P(\mathbf{B}|\theta_{\mathbf{B}}, \mathcal{T}, \mathbf{b}) = \sum_x \pi(x) L_r(x), \text{or} \quad P(\mathbf{T}|\theta_{\mathbf{T}}, \mathcal{T}, \mathbf{b}) = \sum_x \pi(x) L_r(x),$$

where $\pi(x)$ is the equilibrium frequency of root state x. Because, in our setting, we typically can assume that the root has the unmutated state, we force $\pi(x)$ to be equal to 1 for that state and zero otherwise. For the tissue migration process, we do the same for the primary tissue.

A general implementation of the pruning algorithm was used for the tissue-migration process, but for the barcode mutation process, optimizations were available due to irreversibility of the process. In particular, under this model, many ancestral states are not possible and can be excluded to improve efficiency.[19] Let S be the set of all observed indel states at the tips of the tree, including the unedited state (0) and missing data (−1), and let S_j be the set of observed descendant states for internal node j. The allowed ancestral states at node j, A_j, are:

$$A_j = \begin{cases} \{0\} & \text{if } 0 \in S_j \text{ or } \left| S_j \backslash \{-1\} \right| > 1 \\ \{0, s\} & \text{if } S_j \backslash \{-1\} = \{s\} \text{ and } s \neq 0 \\ \mathcal{S} & \text{if } S_j = \{-1\}. \end{cases}$$

The summation in the recursive likelihood calculation can therefore be restricted to $y \in A_j$, assuming that $L_j(y) = 0$ for $y \notin A_j$, substantially improving runtime.

With these assumptions, implementation in BEAST 2 was straightforward. For each sampled or proposed tree topology $\mathcal{T}$, branch lengths $\mathbf{b}$, barcode mutation parameters $\theta_{\mathbf{B}}$, and tissue-migration parameters $\theta_{\mathbf{T}}$, we simply calculated the unnormalized posterior density by multiplying the prior densities and the phylogenetic likelihoods of the conditionally independent barcode data and tissue labels,

$$P(\theta_{\mathbf{B}}, \theta_{\mathbf{T}}, \mathcal{T}, \mathbf{b}|\mathbf{B}, \mathbf{T}) \propto P(\theta_{\mathbf{B}}, \theta_{\mathbf{T}}, \mathcal{T}, \mathbf{b})\ P(\mathbf{B}|\theta_{\mathbf{B}}, \mathcal{T}, \mathbf{b})\ P(\mathbf{T}|\theta_{\mathbf{T}}, \mathcal{T}, \mathbf{b}).$$

We relied on the existing functionality in BEAST 2 to explore the space of tree topologies and parameters. Convergence was assessed by checking that the effective sample size for all parameters exceeds 200, and by visually confirming that parameter traces had reached stationarity.

BEAM parameters and priors

BEAM includes several free parameters that govern the tree topology, mutation dynamics, and tissue migration process. The phylogenetic tree topology is denoted by $\mathcal{T}$, with associated branch lengths $\mathbf{b}=(b_1, b_2, \ldots, b_n)$. The prior over tree topologies and branch lengths is modeled using a birth-death process, with birth rate λ and death rate μ, which are treated as free parameters. The initial tree $\mathcal{T}_0$ can be provided directly in Newick format. In this study, we used LAML to infer a starting tree by approximate maximum likelihood. The barcode mutation model includes a silencing rate parameter l, while the relative rates of non-silencing edit outcomes, $s_1 \ldots s_N$, are fixed and normalized based on the relative frequencies in the observed mutation matrix, as similarly done in Chu et al.[19] and Seidel and Stadler.[53] A strict molecular clock rate ν_b converts real-time branch lengths, which are directly operated on by MCMC proposals for a tree with fixed height from a specified experiment duration time, into number of substitutions before applying the rate matrix exponential to compute transition probabilities for the branch. Tissue migration dynamics are modeled with the relative migration rates between tissues denoted r_{ij}, and a strict clock rate for tissue migration is represented by ν_t. The equilibrium frequency of the primary tissue is denoted π_1, with the frequencies of the remaining tissue types set to a uniform value to ensure that all frequencies sum to one.

The prior distributions assigned to these parameters were as follows:

$$\lambda \sim \text{Uniform}(0,1), \mu \sim \text{Uniform}(0,1),$$

$$l \sim \text{Exponential}(\beta_s), \text{where } \beta_s = 0.1,$$

$$\nu_b \sim \text{Exponential}(\beta_v), \text{where } \beta_v = 0.1,$$

$$r_{ij} \sim \text{Exponential}\left(\beta_q\right), \text{where } \beta_q = 1.0,$$

$$\nu_t \sim \text{Exponential}(\beta_t), \text{where } \beta_t = 0.1,$$

$$\pi_1 \sim \text{Uniform}(0,1).$$

Initial values for parameters were selected based on empirical convergence behavior observed in pilot runs. We used minimally informative priors in this study, but BEAST 2 allows these prior distributions to be adjusted easily, as needed.

Simulation model for cancer evolution and CRISPR lineage tracing

We developed an agent-based model of cancer cell dynamics built on three tools: the Tool for Tumor Progression,[55] the MACHINA simulator,[45] and the Cassiopeia barcode simulator.[14] MACHINA extended the original tumor progression model to include metastasis. Below, we briefly describe the key modeling components.

Cell birth and death follow a multiplicative fitness landscape with logistic constraints. The birth rate of cell c is

$$b(c) = \min\left(1, \max\left(0, 0.5 \times \left(1+0.1\left(1 - \frac{N(\sigma_c)}{50,000 \times |\sigma_c|}\right)\right)^{|\sigma_c|+1}\right)\right),$$

where $N(\sigma_c)$ is the number of cells in the same tissue with driver mutation set σ_c. A cell divides if $r \sim \text{Uniform}(0,1) < b(c)$; otherwise, it dies. Upon division, one daughter retains the parent's genotype, while the other mutates with probability $p = 0.1$. Mutations are drivers with probability $p_d = (2 \times 10^{-7})(|\sigma_c|+1)$, and passengers otherwise. Each generation, birth/death decisions are made for all cells, followed by metastasis decisions for each existing tissue. The probability of migration from tissue t is

$$P_{\text{migration}}(t) = \mu_t \prod_{y \in Y(t)} (N(t,y) \times |y|),$$

where $N(t,y)$ is the number of cells in tissue t with driver mutation set y, and μ_t is the migration rate per cell per driver. Migration occurs from a tissue if $r \sim \text{Uniform}(0,1) < P_{\text{migration}}(t)$ and the number of migrating cells is then drawn from Poisson(1) and migrated to a destination tissue chosen from a uniform transition matrix across ten tissues.

Simulations run for 250 generations, after which cells are downsampled. The lineage tree with branch lengths in cell divisions is then used to simulate CRISPR barcodes at the tips using Cassiopeia, assuming a uniform mutation rate across sites, default heritable silencing rate of 0.0001, and default stochastic silencing rate of 0.01. The output includes a cells-by-barcode matrix, tissue labels, and ground-truth phylogeny and migration history.

Simulated data

To generate simulated datasets under ideal conditions, we ran 100 simulations with a mutation rate of 0.0025 mutations per barcode site per cell division and a migration rate of 1×10^{-6} per cell. These simulations produced a ~50% saturated character matrix and a migration graph of intermediate complexity (see representative examples in Figure S4). To simulate data with variable complexity of barcode mutation and tissue migration, we simulated 20 datasets for each combination of barcode mutation rates {0.0005,0.001,0.0025,0.005,0.01} per barcode site per cell division and tissue migration rates $\{1 \times 10^{-7}, 1 \times 10^{-6}, 1 \times 10^{-5}, 1 \times 10^{-4}\}$ per cell (representatives shown in Figure 2B). Additional data simulation conditions are described within figure captions alongside respective results. For all conditions, we simulated trees with 50 cells after downsampling and used a traversal of the downsampled tissue-labeled tree to obtain the ground-truth migration graph. Simulations in which no migrations occurred were discarded and replaced with new ones until the desired number was reached. We note that for the results shown in Figures 2A and 2B, all simulations were processed with all migration history inference methods with the exception of seven simulations for PathFinder in Figure 2A, and three simulations for MACHINA and 69 simulations for PathFinder in Figure 2B due to long runtimes.

Alternative methods for benchmarking

For consistency and efficiency in benchmarking, we developed a pipeline to process the same simulated datasets by several methods in parallel. Because all methods except BEAM required a fixed lineage phylogeny as input, we selected a single phylogeny inference method for preprocessing and, for each simulated mutation matrix, we applied this method once and used the resulting tree for all downstream steps, except when a different phylogeny inference method was indicated in Figure 2A. Based on published benchmarks and our preference for a probabilistic reconstruction method, we chose the LAML method[19] for phylogeny inference. Notably, LAML uses an approximate maximum-likelihood method to estimate both a topology and branch lengths under a similar mutation model to the one assumed by BEAM. For initialization of LAML, we used the Cassiopeia-Greedy algorithm[14] to generate an approximate tree topology without branch lengths from the mutation matrix.

Once this cell-lineage tree was obtained, we applied the following methods for migration-graph inference (listed in order of increasing sophistication).

Random

Tissue labels at the tips of the input phylogeny were used to randomly assign tissue states to internal nodes, ignoring the tree structure. This approach served as a baseline for migration-graph accuracy under random ancestral state assignments and provided an approximate lower bound for the performance of other methods.

Consensus

For each internal node, tissue labels for all tips in its sub-tree were collected and the most common tissue label among those tips was assigned to the node. This was the simplest possible use of the tree structure for tissue labeling.

Parsimony

We applied our own implementation of the Fitch-Hartigan algorithm[56,57] for the "small parsimony problem" of discrete ancestral state reconstruction. This method minimized the number of migration events on an input cell lineage tree, without refining that tree to resolve polytomies or considering other parsimony criteria.

MACHINA

We applied MACHINA[45] v1.0 in Parsimonious Migration History with Tree Refinement (PMH-TR) mode to both refine a given clone tree into a binary tree, resolving polytomies, and label its internal nodes with tissue assignments to infer a migration history. The MACHINA objective is to minimize, in order: the number of migration events (directed edges in the migration graph), the number of co-migrations (unique edges in the migration graph, so that a single directed edge and a directed multi-edge each only contribute a count of one), and the number of seeding sites (distinct tissues serving as sources of migration) using mixed integer linear programming under a parsimony criterion. We used MACHINA in unrestricted migration mode to allow the migration graph to take any topology rather than being restricted to predefined patterns. MACHINA returned a single most parsimonious labeling.

PathFinder

We used PathFinder[49] v2.0 to infer migration histories from sequence alignments with known tissue labels. PathFinder first builds a Neighbor-Joining tree from the alignment, then applies a probabilistic algorithm to infer migration events and resolve polytomies by permutation of the tree. To generate the input sequence alignment, we converted the indel matrix containing multiple mutations per site into a binary format, representing each mutation as a separate site with "A" for unmutated and "T" for mutated states. We ran PathFinder with default settings, enabling the `relax_threshold` flag to retain low-probability histories that proved necessary to prevent run failures. Although it was originally described as a Bayesian approach, we received clarification from the PathFinder authors that the method was intended to output a single best migration history rather than a properly calibrated posterior distribution of migration histories, so we computed performance metrics on the single best solution.

Metient

We applied Metient[50] v0.1.3.4.13 in a similar way to MACHINA. Metient minimizes the same three criteria of migrations, co-migrations, and seeding sites while resolving polytomies in the tree, similar to MACHINA, but it allows flexibility in how these criteria are weighted. We ran Metient-evaluate using the default parsimony weights in `pancancer_genetic_uniform_weighting()` (0.5448 for migrations, 0.2727 for co-migrations, and 0.1825 for seeding sites) that were recommended by the authors for non-human data. We also tested Metient-calibrate on our simulated data, which found different optimal weights but led to worse performance, so

we report results using the default weights and Metient-evaluate. Tissue locations for tips were encoded in the input metadata as present and nodes were marked absent from all tissue locations. We used the default settings for Metient-evaluate except that we applied `solve_polytomies = True`. For precision and recall based performance metrics, we integrated across all output solutions to obtain an edgewise probability graph. Specifically, to get probabilities for all output solutions, the loss scores were min-max normalized before applying temperature-scaled softmax with a fixed temperature of 0.5, as recommended by the authors.

MACH2

Finally, we applied MACH2[51] v1.0.1, an extension of MACHINA which solves the same PMH-TR problem but enumerates the full set of equally parsimonious migration histories rather than returning a single solution. All solutions are derived from the same integer linear programming formulation used in MACHINA, but MACH2 leverages combinatorial properties of the solution space to exhaustively enumerate all optimal solutions. We ran MACH2 in the default mode and the output solutions were all weighted equally (as in[51]) to construct an edgewise probability migration graph summarizing the full solution set.

Precision, recall, and F1 score calculations

To evaluate migration graph inference, we assessed the precision and recall of inferred edges in terms of true positive (TP), false positive (FP), and false negative (FN) edges in the graph. Importantly, both the simulated and inferred migration graphs are actually *directed multigraphs*, with the possibility of multiple edges between each pair of nodes. Therefore, motivated by ref.,[51] we computed precision and recall using *counts* of each type of edge per graph rather than by considering their presence/absence. This strategy penalizes cases where the types of migration events are correctly inferred but the numbers of events are not—a kind of error that is particularly relevant when evaluating the clonality of seeding.

Given a true migration graph G and an inferred migration graph G^* let E_{ij} and E^*_{ij} represent the numbers of directed edges in G and G^*, respectively, that run from tissue i to j where $i \neq j$. The counts TP, FP, and FN were derived from these edge counts as follows:

$$\mathrm{TP} = \sum_{i \neq j} \min\left(E_{ij}, E^*_{ij}\right), \mathrm{FP} = \sum_{i \neq j} \max\left(E^*_{ij} - E_{ij}, 0\right), \mathrm{FN} = \sum_{i \neq j} \max\left(E_{ij} - E^*_{ij}, 0\right).$$

The precision (Pr), recall (Re), and F1 score were then calculated by the standard formulas:

$$\mathrm{Pr} = \frac{\mathrm{TP}}{\mathrm{TP+FP}}, \mathrm{Re} = \frac{\mathrm{TP}}{\mathrm{TP+FN}}, \mathrm{F1} = 2 \times \frac{\mathrm{Pr} \times \mathrm{Re}}{\mathrm{Pr+Re}}.$$

For precision-recall curves from MCMC samples, we first converted each sample s of a tissue-labeled tree into a migration multigraph, with an integer count $E^{(s)}_{ij}$ for each type of edge (i,j). We then let $f_{ij}(c)$ be the fraction of such sampled multigraphs that have c or more counts of edge (i,j), that is, the fraction with $E^{(s)}_{ij} \geq c$. Because it is derived from samples from the approximate posterior distribution, this fraction $f_{ij}(c)$ can be interpreted as an estimate of the posterior probability that the inferred edge count $E^*_{ij} \geq c$. We therefore subjected this value to a varying posterior-probability threshold, p, such that $0 \leq p \leq 1$. For each choice of p, we estimated E^*_{ij} as the maximum number of edges c such that $f_{ij}(c) \geq p$. We then evaluated the counts TP, FP, and FN using that value of E^*_{ij}. For the F1 score calculations, we fixed the threshold p at 0.5.

We used the same strategy for MACH2, treating each reported solution as if it were a draw from a posterior distribution (so that they were equally weighted). For Metient, we obtained the posterior distribution over solutions through min-max normalization and temperature-scaled softmax, as recommended by the authors, and then weighed solutions by their posterior probability when computing an edgewise probability graph to then apply thresholds to.

We followed ref.[87] in using precision-recall curves rather than receiver operating characteristic (ROC) curves, which are problematic in the multigraph setting.

Calculating excess migrations relative to parsimony

To calculate the excess migrations predicted by BEAM, we began with a sample from the joint posterior distribution over tissue-labeled trees and model parameters given the data. For each sampled tree, the number of BEAM-inferred migration events was obtained in the usual way, by counting branches in the tree for which the parent and child tissue labels disagreed. The tissue labels were then removed from the internal nodes of the tree and our implementation of the Fitch-Hartigan parsimony algorithm was applied to obtain an estimate of the minimum possible number of migration events given the leaf labels only. This minimum possible count was subtracted from the BEAM estimate to obtain an estimate of the excess migrations per sample. Finally, an estimate of the posterior expected excess migration count was obtained by averaging these per-sample excess counts across all samples.

Application to lung, prostate, and pancreatic cancer datasets

As detailed in ref.,[34] the lung cancer data was derived from orthotopically xenografted KRAS-mutant A549 lung adenocarcinoma cells, engineered with CRISPR barcodes and surgically implanted into the left lungs of four mice. As in the published analysis, we focused on data for one mouse (labeled "5k") with 100 clonal populations (CPs), which were tracked over 54 days. We began with the provided allele table and metadata for mouse 5k. We excluded the 17 CPs excluded in the original paper (5, 16, 18, 25, 33, 38, 39, 41, 50, 53, 65, 69, 75, 81, 87, 88, 93), 9 CPs with only one observed tissue label (22, 29, 46, 48, 49, 78, 85, 94, 96), and 21 large CPs (1–4, 6–15, 17, 19–23, 27, 31) that were computationally prohibitive to analyze, and analyzed the remaining 53

CPs with BEAM. To infer tissue migration graphs, we used the coarse-grained tissue annotations, specifically the metadata labels LL, M, RL, and Liv, which were mapped to each cell barcode.

The prostate cancer data was obtained from a somatically engineered mouse with knockout of PTEN/P53[88] and the introduction of CRISPR barcodes. Cancer developed in 10 mice over an average of ~380 days, producing a variable number of CPs per mouse. The prostate cancer datasets for all mice were re-processed using the EvoTraceR pipeline (v1.0.4), following the authors' original analysis protocol, resulting in 522 CPs. We excluded 2 CPs (MMUS1875 CP1 and MMUS1466 CP1) that were computationally prohibitive to analyze and we excluded 99 CPs with only one observed tissue label. This left us with 421 CPs to analyze across the 10 mice with BEAM. With MACH2 analyses, we also had to exclude 3 more CPs (MMUS1469 CP1 and 3; MMUS1495 CP1) due to long runtimes.

For both datasets, these preprocessing steps produced mutation matrices and associated tissue labels for each CP. We then inferred a baseline tree for each CP using the Cassiopeia-Greedy algorithm[14] and supplied this tree to LAML[19] for tree and branch-length estimation by maximum likelihood. These LAML-estimated lineage trees were later used both as the starting trees for BEAM and as the fixed input trees for all other migration-graph inference methods.

We incorporated two additional reference datasets for a supporting comparison of CRISPR barcode mutational content in metastatic CPs. From a published pancreatic cancer dataset[44] with 83 total clones from two mice, we retained 20 metastatic clones (clones 2, 4, 5, 9, 10, 11, and 22 from mouse M1 and clones 1, 2, 4, 5, 6, 9, 15, 19, 21, 23, 25, 29, 63 from mouse M2) after filtering out 57 clones with only one tissue label, one clone that was too large to analyze with BEAM, and 27 clones with no phylogenetically informative mutations (22 of which overlap with those clones having only one tissue label). We then used the provided mutation matrices directly with minor preprocessing to convert them into the necessary format for our analyses. From an additional lung cancer dataset,[35] we retained five metastatic clones (3457_Apc_T1, 3457_Apc_T4, 3508_Apc_T2, 3513_NT_T1, and 3519_Lkb_T1 all formatted as mouse_genotype_clone) from the nine clones available in the deposited data after filtering out four CPs too large to analyze with BEAM. We derived mutation matrices from the provided overall allele table in a similar manner to our pre-processing of the main lung cancer dataset,[34] since both datasets originated from the same laboratory and reported similar pre-processing steps within Cassiopeia,[14] and we again used the coarse-grained tissue annotations here that merge multiple samples from the same tissue. All preprocessing scripts were made publicly available, see Data and code availability.

Previously reported levels of M2M and PR for real data

For the lung cancer dataset, we used the numbers of CPs reported to exhibit metastasis-to-metastasis (M2M) seeding and primary reseeding (PR) under the labels "metastatic cascade" and "reseeding" in Figure 7 of ref.[34] across all CPs that they analyzed. For the prostate cancer dataset, however, the published analysis reported fractions of edges in full lineage trees within or across CPs,[37] which did not directly correspond to our metrics. Therefore, we obtained the MACHINA migration-graph output files from the authors and recalculated the percentage of CPs showing evidence of M2M or PR events across all CPs in their study. Specifically, a CP was labeled as M2M if it had any edge excluding the primary tissue, and as PR if it had an edge with a non-primary source tissue and the primary tissue as the target tissue.

Hypothesis testing with Bayes factors

Bayes factors are a flexible and powerful means for model comparison and hypothesis testing in a Bayesian setting, but they require evaluation of the marginal likelihood of the data, integrating over parameters and any other latent variables. In our case, the marginal likelihood given the model M has the formidable form:

$$P(\mathbf{B},\mathbf{T}|M) = \sum_{\mathcal{T}} \iiint P(\mathbf{B}|\theta_{\mathbf{B}},\mathbf{b},\mathcal{T}) P(\mathbf{T}|\theta_{\mathbf{T}},\mathbf{b},\mathcal{T}) P(\theta_{\mathbf{B}},\theta_{\mathbf{T}},\mathbf{b},\mathcal{T})\, d\theta_{\mathbf{B}}\, d\theta_{\mathbf{T}}\, d\mathbf{b}.$$

This expression—a version of what is sometimes called the "Felsenstein equation"[89,90]—is both a sum over all possible tree topologies and an integral over all possible combinations of branch lengths and other parameters, making it highly intractable.

Nested sampling, however, offers an effective approach for approximating this integral by MCMC, and a convenient package for nested sampling is already available for BEAST 2.[91] This approach works by mapping the parameter space to a prior mass using a cumulative density function,[92] thereby transforming the high-dimensional integral into a one-dimensional integral over prior mass. The integral is approximated by sequentially sampling parameter sets of increasing likelihood and computing a weighted sum of these likelihoods, where the weights correspond to the changes in prior mass between samples.

Once marginal likelihoods are available for two models M_1 and M_2, a Bayes factor can easily be computed as a ratio of their marginal likelihoods. In the log space used by BEAST 2,

$$\ln(\text{Bayes factor}) = \ln\left[\frac{P(\mathbf{B},\mathbf{T}|M_1)}{P(\mathbf{B},\mathbf{T}|M_2)}\right] = \ln[P(\mathbf{B},\mathbf{T}|M_1)] - \ln[P(\mathbf{B},\mathbf{T}|M_2)].$$

We simply interpreted values of *ln*(Bayes factor)<-1.1 as indicating positive support for M_2, values of *ln*(Bayes factor) > 1.1 as indicating positive support for M_1 and values between -1.1 and 1.1 (inclusive) as not positively supporting either model (see[93]).

In our case, we began by running nested sampling with 10,000 MCMC iterations per sub-chain and a single active particle. We then increased the number of active particles as much as computationally feasible and following the recommendations of the "Taming the BEAST" tutorial.[94]

Calculating mutual information of tissue transitions

For a simple, model-free evaluation of the information in the data relevant to migration history in the posterior distribution, we computed the mutual information between the sampled tissue assignments at parent and child nodes across all branches of the tree. First, a collection of trees sampled by BEAM was traversed to fill an $n \times n$ matrix of counts C, where n is the number of distinct tissue labels, and C_{ij} represented the total number of inferred transitions from tissue i to tissue j across branches. The count matrix was then normalized to obtain a matrix of joint probabilities of pairs of tissues, $P_{ij} = \frac{C_{ij}}{\sum_{i,j} C_{ij}}$, as well as vectors of marginal probabilities, $P_{i.} = \sum_j P_{ij}$ and $P_{.j} = \sum_i P_{ij}$. The mutual information between the source tissue X and target tissue Y was then calculated as:

$$I(X;Y) = \sum_{i=1}^{n} \sum_{j=1}^{n} P_{ij} \log_2 \left(\frac{P_{ij}}{P_{i.} P_{.j}} \right).$$

To normalize for differences in entropy between datasets, the mutual information was scaled by the average entropy of the marginals:

$$I'(X;Y) = \frac{2 \times I(X;Y)}{H(X)+H(Y)},$$

where $H(X) = -\sum_i P_{i.} log_2(P_{i.})$ and $H(Y) = -\sum_j P_{.j} log_2(P_{.j})$.

Since BEAM models tissue transitions as a Markov process, where ancestral tissue labels are more likely to persist within the same tissue along the cell lineage tree rather than to result from frequent migration between tissues, we expected a well-fit model to produce a high normalized mutual information score.

Evaluating the PR test on simulated data

All ground-truth PR simulations from the variable rates simulated dataset in Figure 2B were collected, excluding any with a mutation rate of 0.0005 or a migration rate of 1×10^{-4} due to poor performance in prior evaluations or a migration rate of 1×10^{-7} due to a lack of ground truth migration graphs with PR. This resulted in 39 valid PR simulations. All non-PR ground truth simulations were then gathered, excluding those with migration rate of 1×10^{-4}, migration rate of 1×10^{-7}, or mutation rate of 0.0005 and 39 of them were randomly sampled to match the number of PR simulations for class balance. We then applied the PR hypothesis testing procedure to all 78 simulations. Classification was based on the Bayes factor, where simulations with ln(Bayes factor) > 1.1 were classified as PR and ln(Bayes factor)<-1.1 as non-PR. This classification scheme ignores simulations classified as not supporting either model with $-1.1 \leq ln$(Bayes factor)≤ 1.1, so we provide results for all three outcome categories.

QUANTIFICATION AND STATISTICAL ANALYSIS

Quantitative and statistical analyses are described in the relevant Method details sections and figure legends. All analyses were performed in Python.

Cell Genomics, Volume *6*

Supplemental information

Bayesian inference of tissue-migration histories in metastatic cancer from cell-lineage tracing data

Stephen J. Staklinski, Armin Scheben, Lise M. Brault, Rebecca Hassett, Ryan N. Serio, Jiawei Xing, Dawid G. Nowak, and Adam Siepel

SUPPLEMENTARY FIGURES

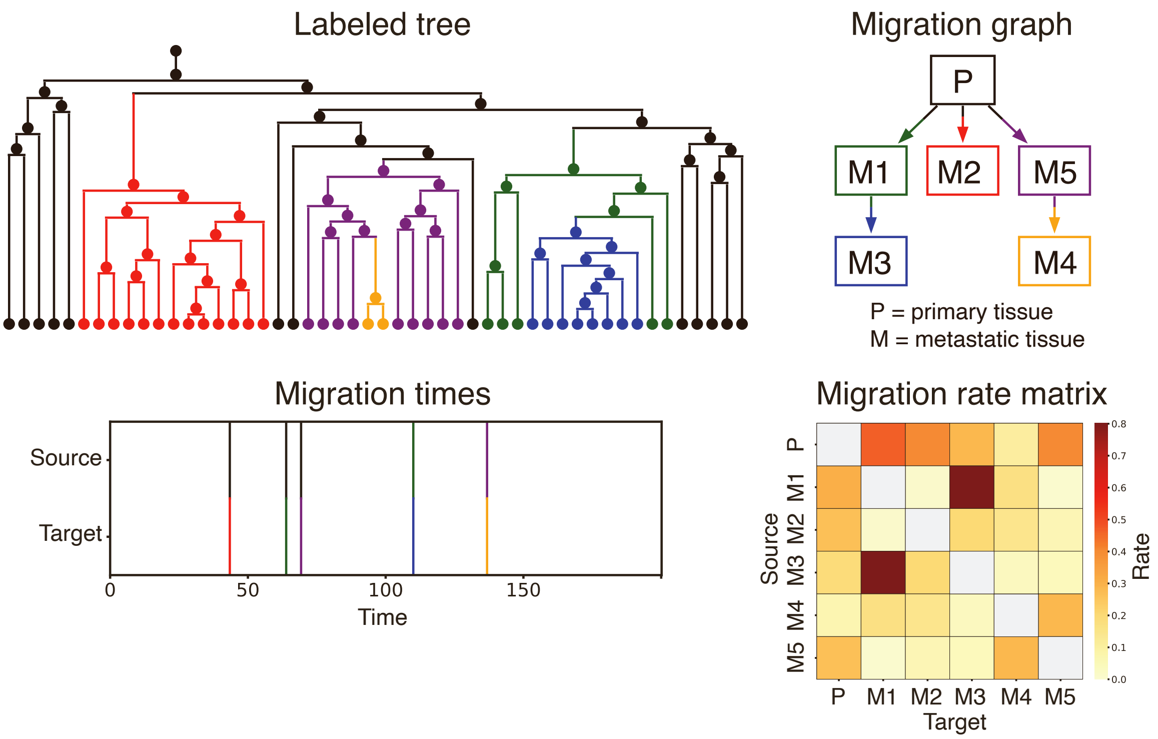

Figure S1. Example posterior samples from joint inference of lineage trees, migration graphs, and model parameters, Related to Figure 1
An example of a sample from BEAM's posterior distribution, including a labeled tree (top left), the corresponding migration graph (top right) and derived migration times (bottom left). The migration rate parameters (bottom right) are also sampled from the posterior distribution.

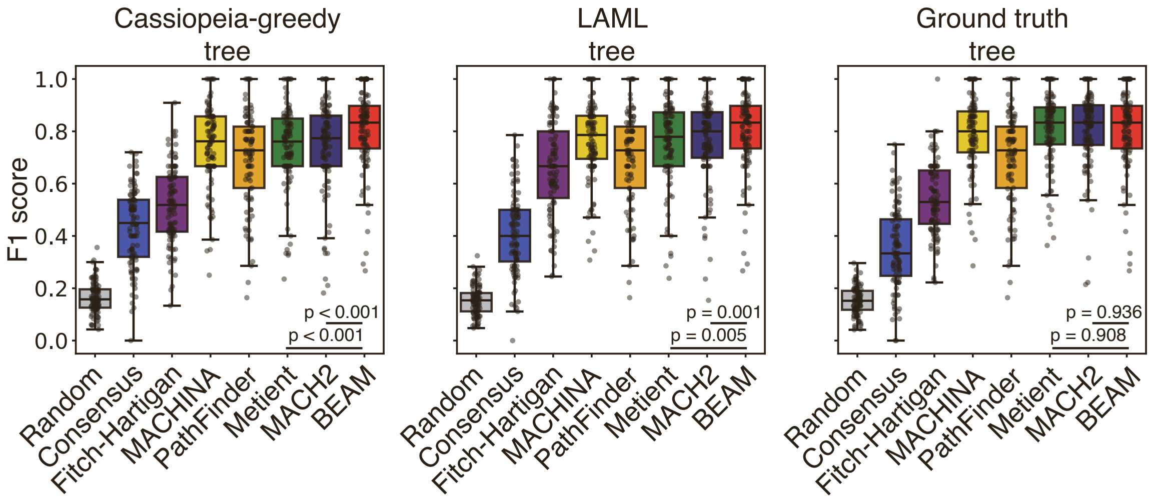

Figure S2. F1 score comparison for migration-graph inference methods run on simulated data, Related to Figure 2

F1 scores for the same experiment as in **Figure 2A**, based on a threshold of 0.5 posterior probability for Metient, MACH2, and BEAM. Reported p-values are from paired t-tests only between MACH2 and Metient compared to BEAM. Again, inference results are shown for each method, conditioned on the input tree topology inference method indicated above each plot. Because PathFinder and BEAM do not rely on a fixed input tree, the same results are displayed for these two methods across all plots.

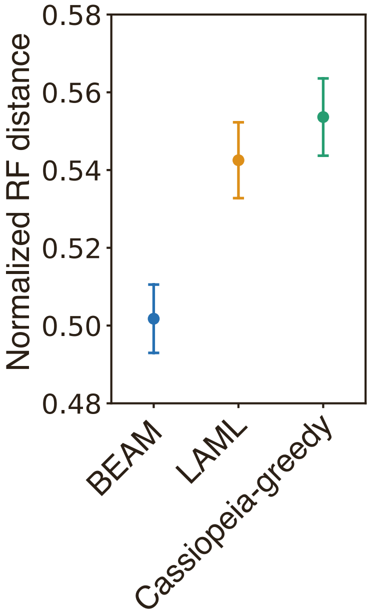

Figure S3. Tree reconstruction accuracy across inference methods measured by Robinson–Foulds distance, Related to Figure 2
Mean normalized Robinson–Foulds (RF) distance measuring tree reconstruction accuracy for the simulations shown in **Figure 2A**, comparing Cassiopeia-greedy, LAML, and BEAM inference results. The normalized RF distance is calculated as the symmetric difference between the sets of bipartitions in the true and inferred trees, divided by the total number of bipartitions across both trees. Trivial bipartitions (those separating a single leaf from all others) are excluded from the calculation. The expectation over the distances for all posterior distribution samples was calculated for BEAM. Points represent the mean ± standard error of the mean across replicate simulations.

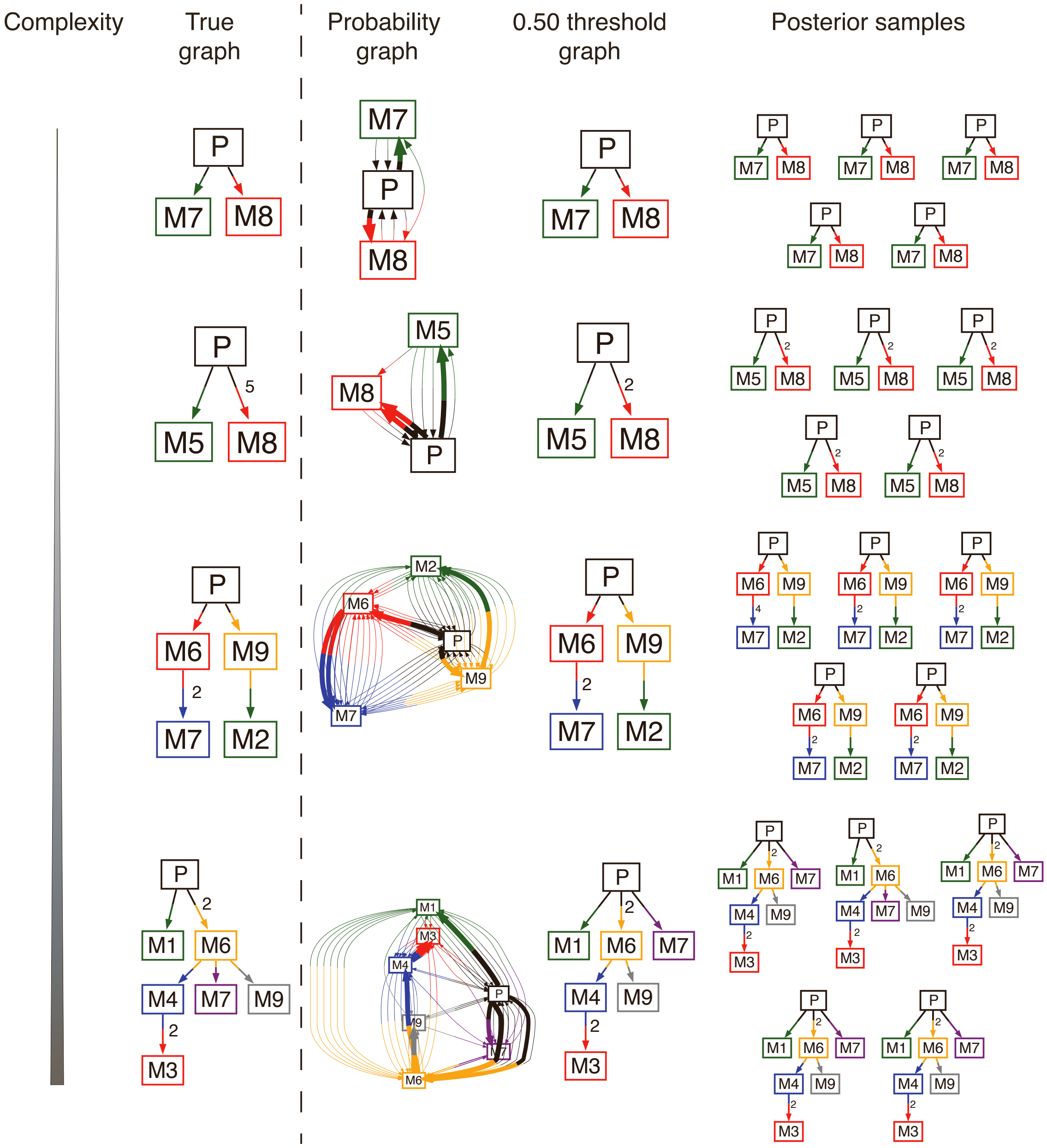

Figure S4. Representative posterior migration graphs for simulated datasets of increasing complexity, Related to Figure 2

Representative graphs sampled from BEAM's posterior distribution for simulated datasets of increasing complexity in the favorable parameter regime shown in **Figure 2A**. Shown for each case are the true migration graph, the edgewise probability-weighted graph, the 0.5-threshold graph, and individual samples from the posterior distribution. Numbers next to edges indicate multi-edge counts. Edges without a number represent single migration events. The P label represents the primary tissue and labels beginning with M are metastatic tissues in the simulations.

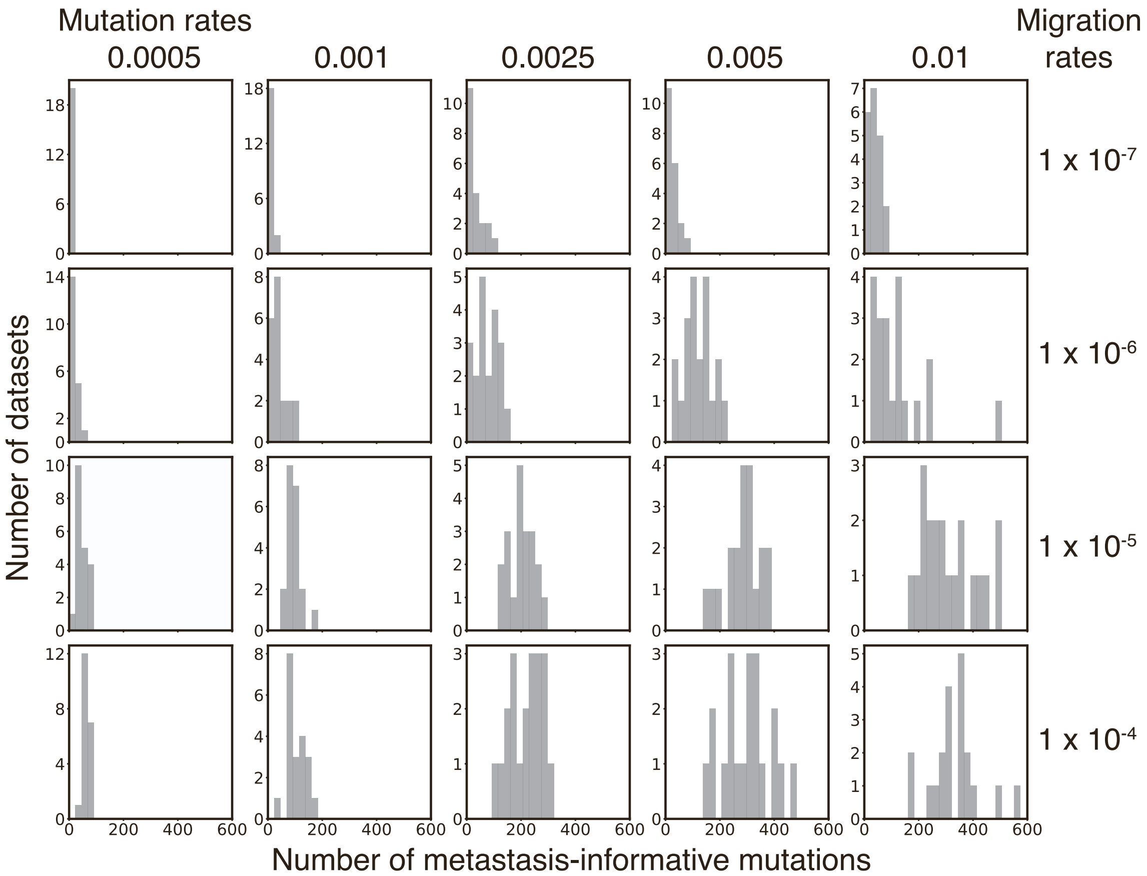

Figure S5. Number of metastasis-informative mutations across simulated mutation and migration regimes, Related to Figure 2

Number of metastasis-informative mutations per simulation for the variable parameter simulations shown in **Figure 2B**. A metastasis-informative mutation was defined as a mutation occurring on a migration branch in the ground-truth tree, where the parent and child tissues differ. Migration branches were identified directly from the simulation output. Mutations were mapped to branches by inferring the accumulated indel states at each internal node using a post-order traversal that intersected child node states, followed by a pre-order pruning step to retain only new mutations arising along each branch. The total number of metastasis-informative mutations was then calculated as the sum of new mutations occurring on all migration branches.

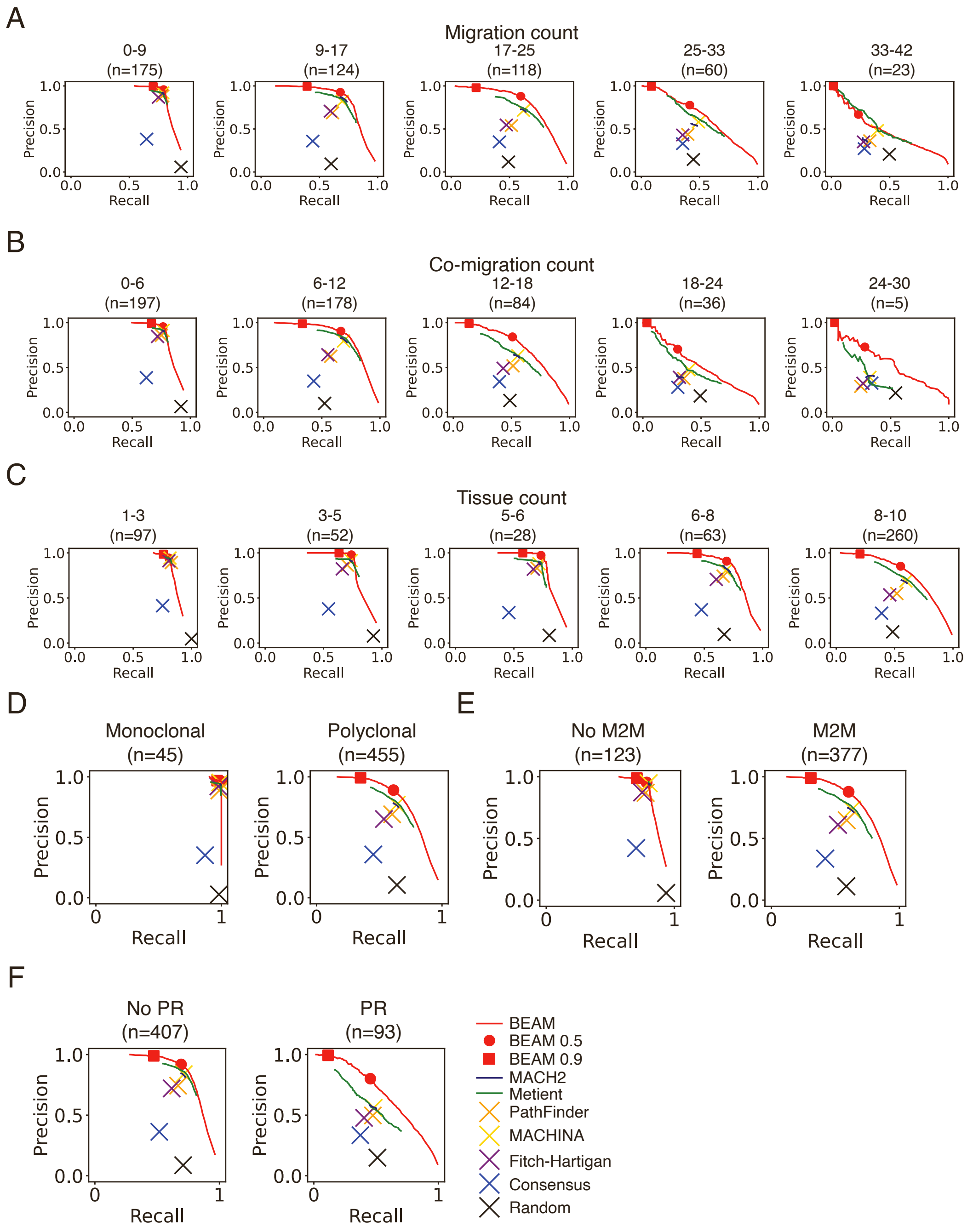

Figure S6. Precision–recall performance stratified by simulated migration-graph properties, Related to Figure 2

Precision-recall curves for 500 total simulated datasets from the combination of **Figure 2A–B** stratified by: (A) number of migration events (directed edges in the graph), (B) number of co-migration events (unique edges in the graph, so that a single directed edge and a directed multi-edge each only contribute a count of one as in[S4?,S5]), (C) number of tissues, (D) clonality (whether any multi-edges exist or not in the graph for polyclonal and monoclonal respectively), (E) presence of metastasis-to-metastasis (M2M) seeding, and (F) presence of primary reseeding (PR). For plots where groups are defined by ranges, each range excludes its left endpoint and includes its right endpoint. For methods that require an input phylogeny, the results are shown using the tree inferred by LAML.

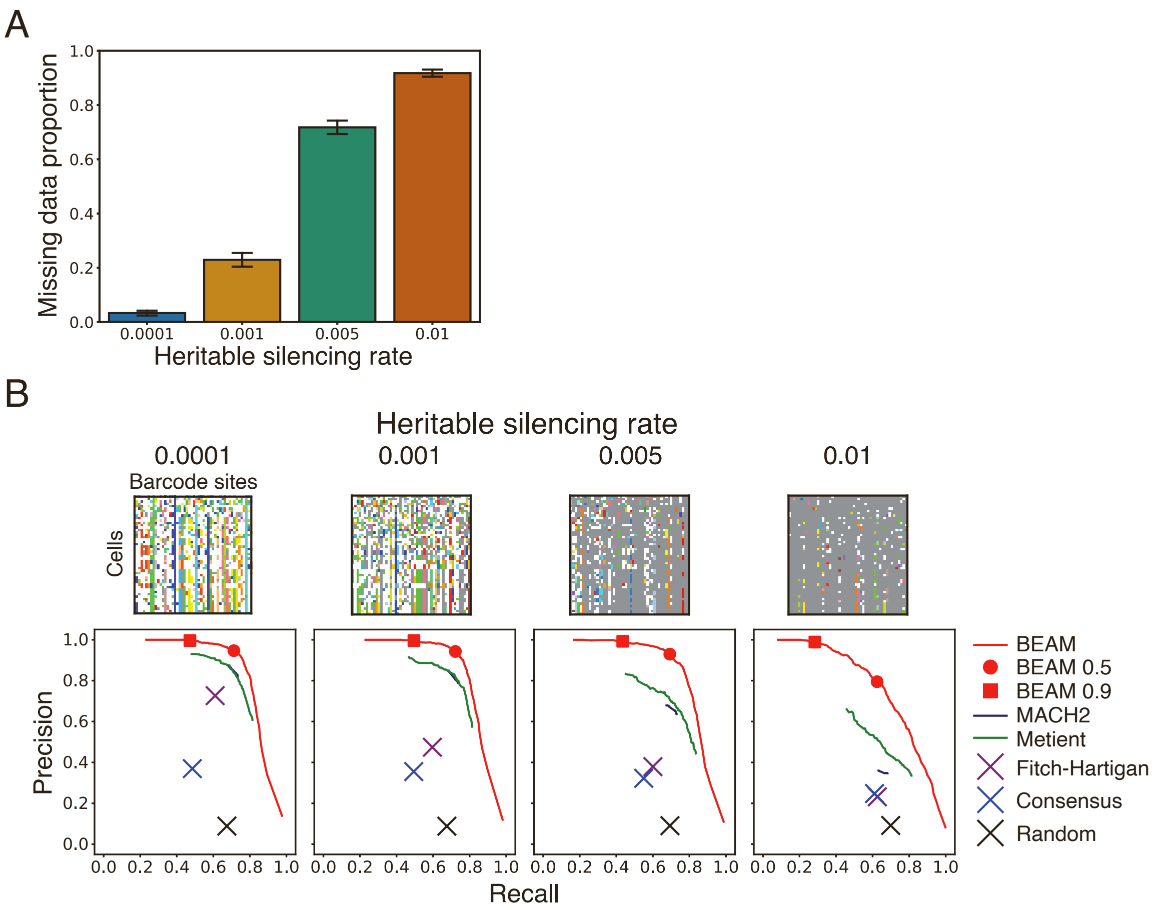

Figure S7. Effects of CRISPR barcode missing data on migration-graph inference accuracy, Related to Figure 2

(A) Proportion of missing mutation matrix entries across simulated datasets for each heritable silencing rate. Bars summarize 100 simulations generated using the same ground truth migration histories as in **Figure 2A**, with new barcode data overlaid using the indicated heritable silencing rates. Bars are shown as mean $\pm$ standard deviation (B) Representative barcode matrices (top), with gray color indicating missing data entries, and precision–recall curves (bottom) for BEAM, MACH2, Metient, Fitch–Hartigan parsimony, Consensus, and a Random baseline across the same heritable silencing-rate range. BEAM point estimates at posterior probability thresholds 0.5 and 0.9 are shown. Methods requiring an input phylogeny use the LAML-inferred tree. Results for the 0.0001 heritable silencing rate come directly from **Figure 2A**. MACHINA and PathFinder are excluded in the 0.0001 silencing rate plot for consistency across other silencing-rate groups, as they were not run for all settings due to their generally lower performance and longer runtimes relative to MACH2 and Metient.

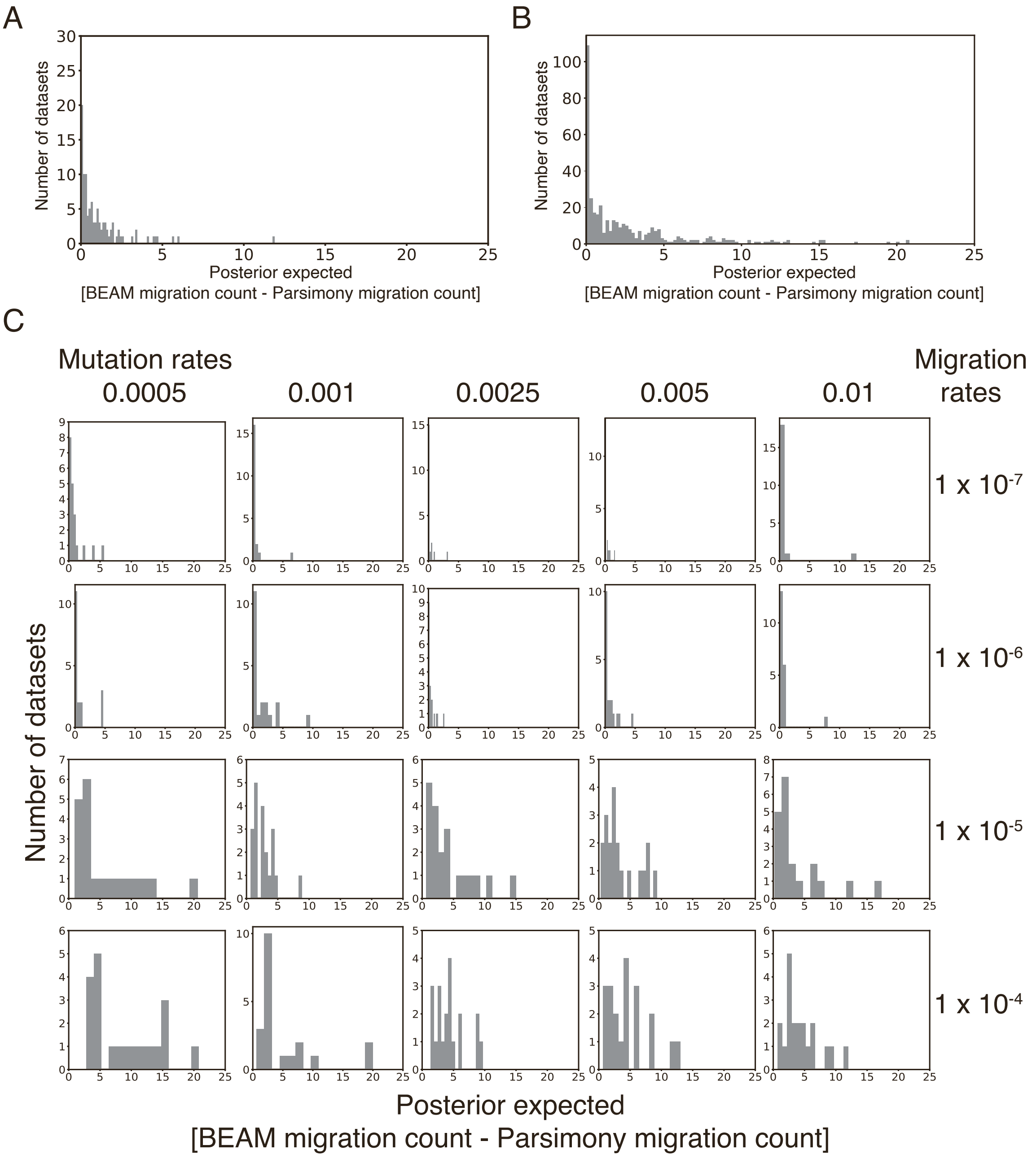

Figure S8. Posterior excess migration counts relative to parsimony-based solutions, Related to Figure 2

Histogram of the excess migrations predicted by BEAM relative to the Fitch-Hartigan parsimony solution for the same trees (see text) in: (A) the favorable parameter regime shown in **Figure 2A**; (B) the variable parameter regime shown in **Figure 2B**; and (C) the variable parameter regime stratified by mutation and migration rate.

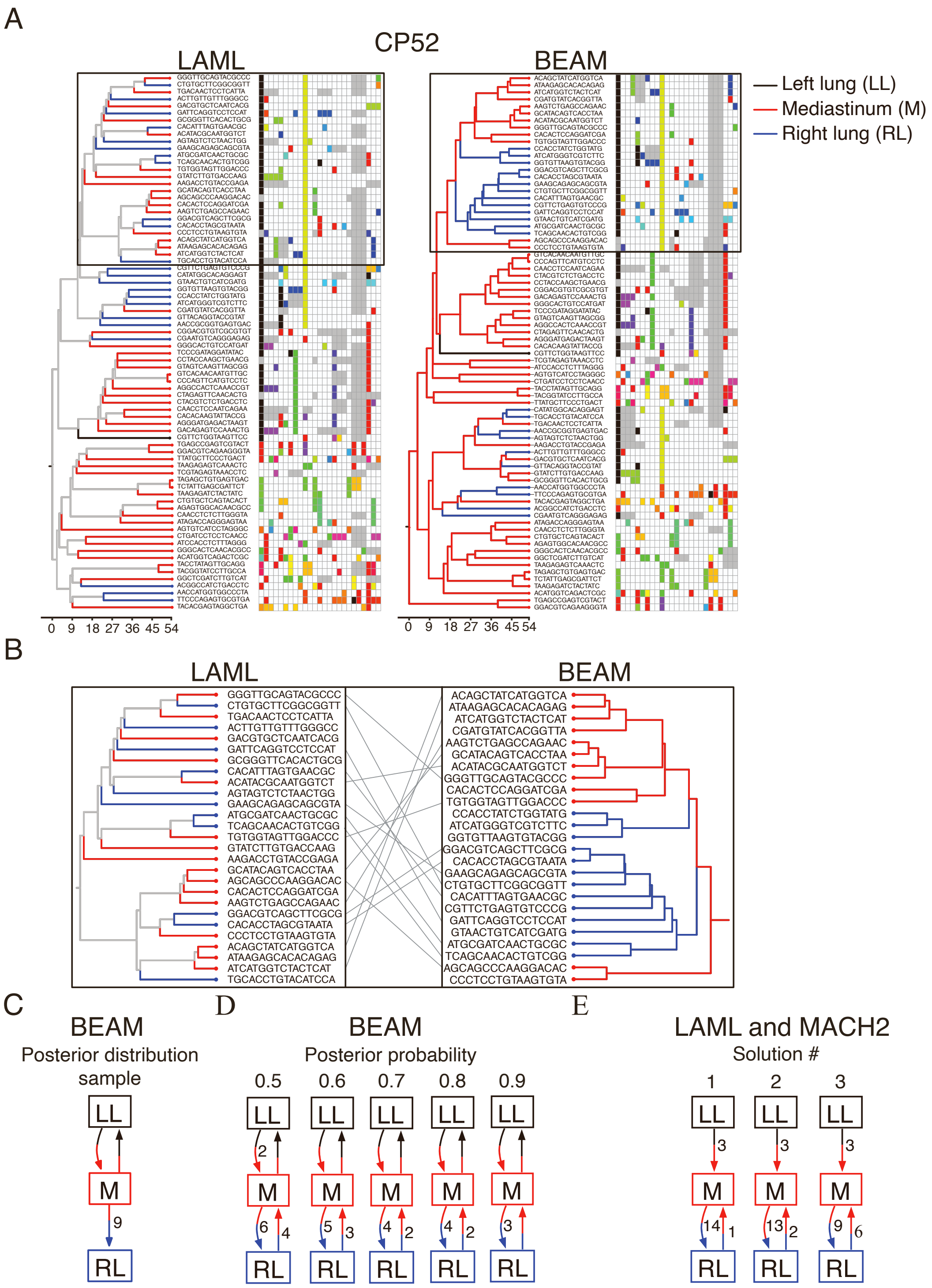

Figure S9. Example lineage trees and migration graphs with differing inferred topologies across methods, Related to Figure 3

(A) Lung cancer lineage trees for CP52 as inferred by LAML (left) and BEAM (right). LAML inferred only the tree topology, so internal nodes are gray, while BEAM resolved internal nodes by tissue. The matrix next to each tree shows barcode mutations per cell with white as unedited, gray as missing, and each color representing a mutation (with colors repeating if there are too many unique mutations). (B) Zoomed-in view of a clade with topological differences between LAML and BEAM. Gray lines map tips present for both methods. (C) Migration graph for the BEAM lineage tree posterior sample in (A). (D) BEAM migration graphs at increasing edgewise probability-retention thresholds. (E) MACH2 migration graph resolved from the LAML tree.

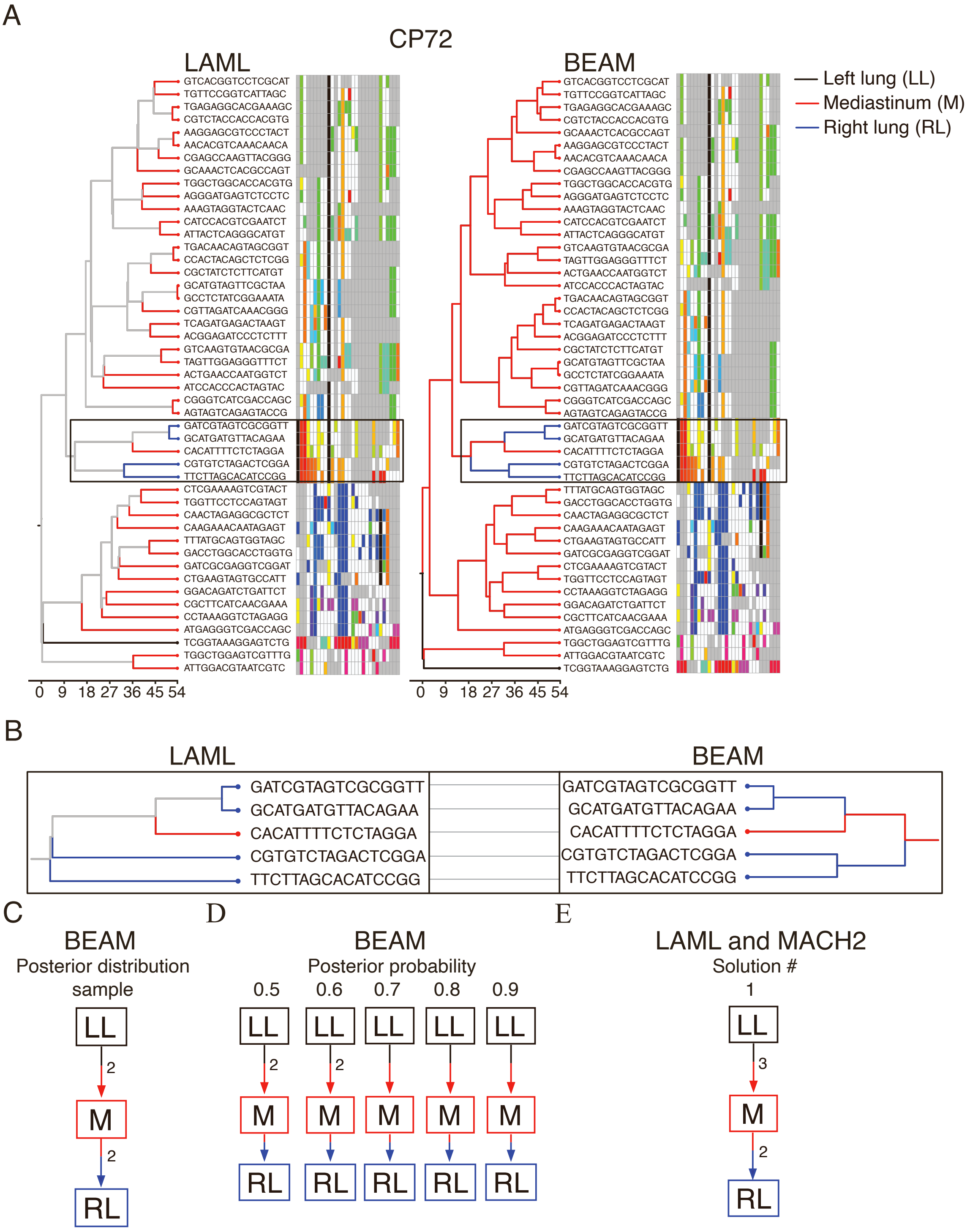

Figure S10. Example lineage trees and migration graphs with similar inferred topologies across methods, Related to Figure 3
(A–E) Same as **Figure S9** but for lung cancer CP72.

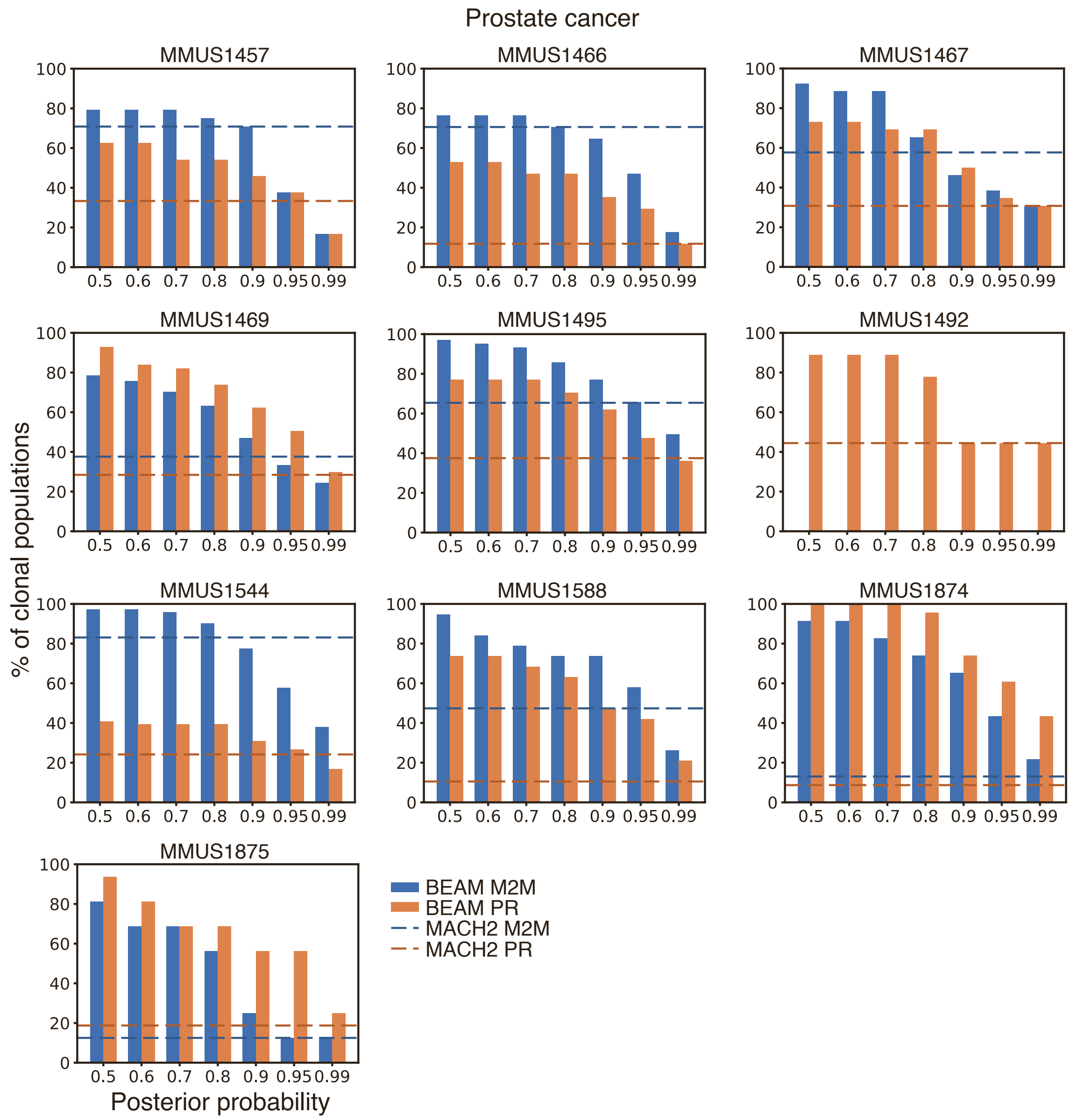

Figure S11. Detection of metastasis-to-metastasis and primary reseeding events in prostate cancer stratified by mouse, Related to Figure 3
Same as **Figure 3A** for the prostate cancer data plotted per mouse.

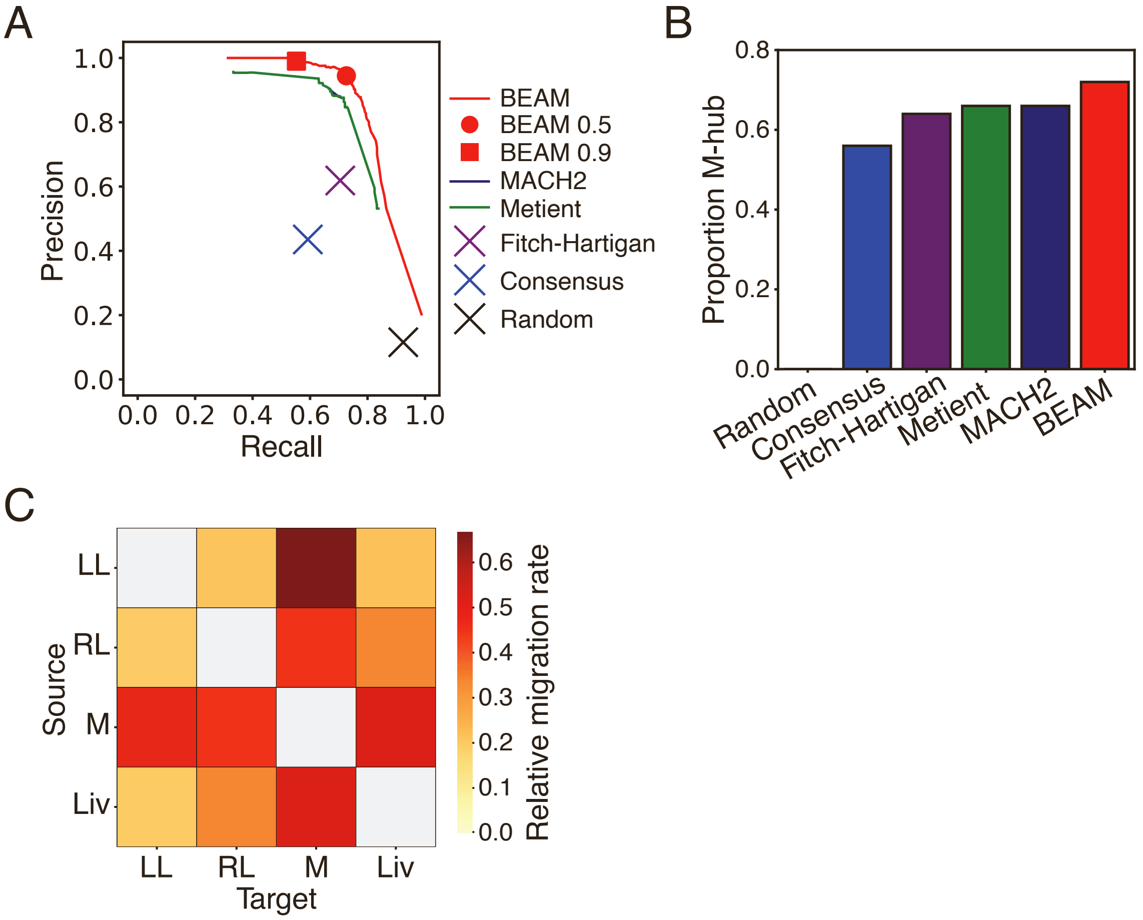

Figure S12. Simulation-based evaluation of M-hub migration patterns, Related to Figure 3 (A) Precision–recall performance for 50 simulations modeled after the real lung cancer dataset, with four tissues, LL as the primary tissue, and only LL$\rightarrow$M migrations permitted (transition probability 1.0, all other primary-to-met transitions set to 0 to reproduce the observed M-hub pattern. All other migrations among metastatic tissues were simulated uniformly. Datasets were generated using a mutation rate of 0.0025 and migration rate of 1×10^{-6}, matching our standard conditions. We compared BEAM (showing both the curve and point estimates at posterior probability thresholds 0.5 and 0.9) with MACH2, Metient, Fitch–Hartigan parsimony, Consensus, and Random tissue-labeling baselines. (B) Proportion of simulations in which each method correctly recovered the migration graph as an M-hub, defined as LL seeding only M and subsequent migrations originating from M to other tissues. Bars show the fraction of simulations whose inferred graph matched this pattern. (C) Mean posterior instantaneous migration rates between tissues inferred by BEAM, averaged across all simulations under the normalized CTMC migration model from each source tissue to each target tissue. We use the LL, RL, M, and Liv labels to match the lung-cancer dataset, but the internal simulated tissue labels were generic as in our other simulations, so we simply reassigned labels arbitrarily while respecting the primary tissue and M-hub structure.

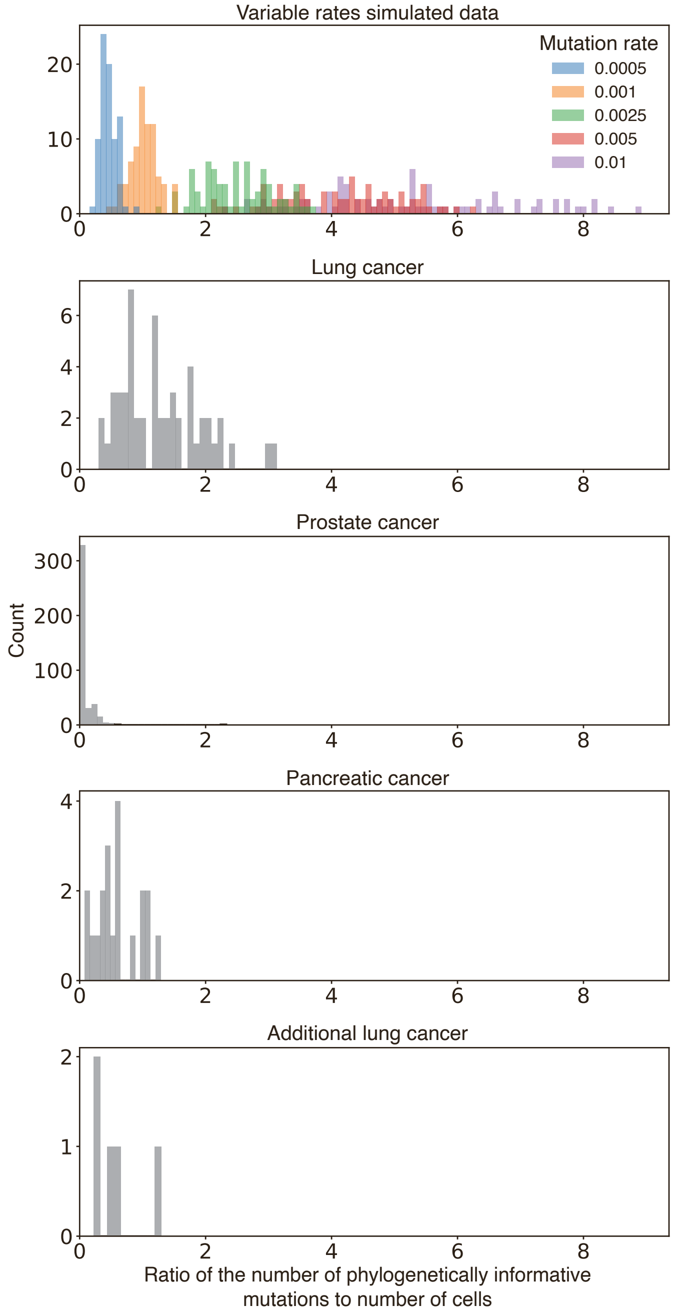

Figure S13. Phylogenetically informative mutation content across real and simulated datasets, Related to Figure 3 and Figure 4
The ratio of phylogenetically informative mutations to the number of cells (tree tips) was calculated for each clonal population in the real and variable-rate simulated datasets included in this study. "Lung cancer" and "Prostate cancer" correspond to the main datasets analyzed[S3,S3], while "Pancreatic cancer" and "Additional lung cancer" are additional external CRISPR barcode datasets[S3,S4]. At each barcode site (column) in the mutation matrix, unique informative mutations were defined as the set of those mutations present in two or more cells (rows), but not all cells. Counts were summed across barcode sites and divided by the number of cells in the clonal population. Bars in the variable-rate simulated dataset are colored by simulated mutation rate for reference.

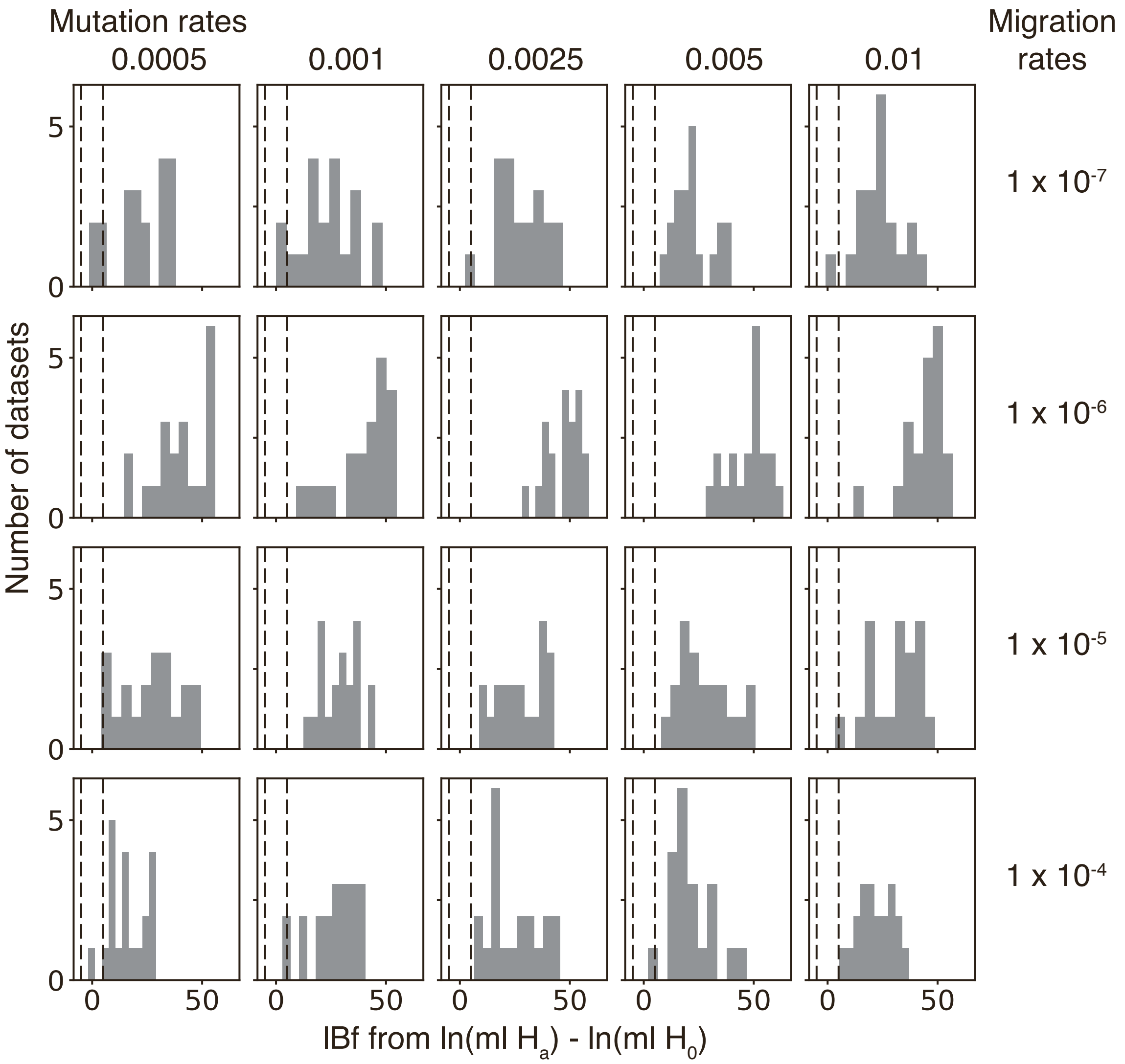

Figure S14. Information-content hypothesis testing in variable-rate simulated datasets, Related to Figure 4

Application of the random vs. GTR information content hypothesis test in **Figure 4A** to the variable rates simulated data from **Figure 2B**. The reported values are the log Bayes factor (lBf) from the comparison of the two models and the dashed lines indicate the classification thresholds at -1.1 in favor of the random model and 1.1 in favor of the GTR model.

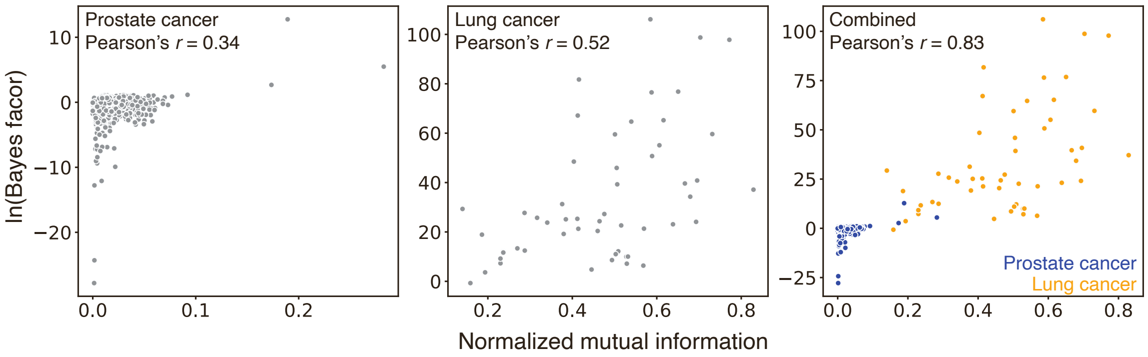

Figure S15. Relationship between Bayes factor and posterior mutual information for quantifying dataset informativeness, Related to Figure 4
Relationship between the log Bayes factor from the random vs. GTR information content hypothesis test in **Figure 4A** (y-axis) and the normalized mutual information (x-axis) computed from tissue transition count matrices across BEAM posterior distributions (see **STAR METHODS**). The prostate cancer data is shown on the left, the lung cancer data in the center, and the combined data on the right.

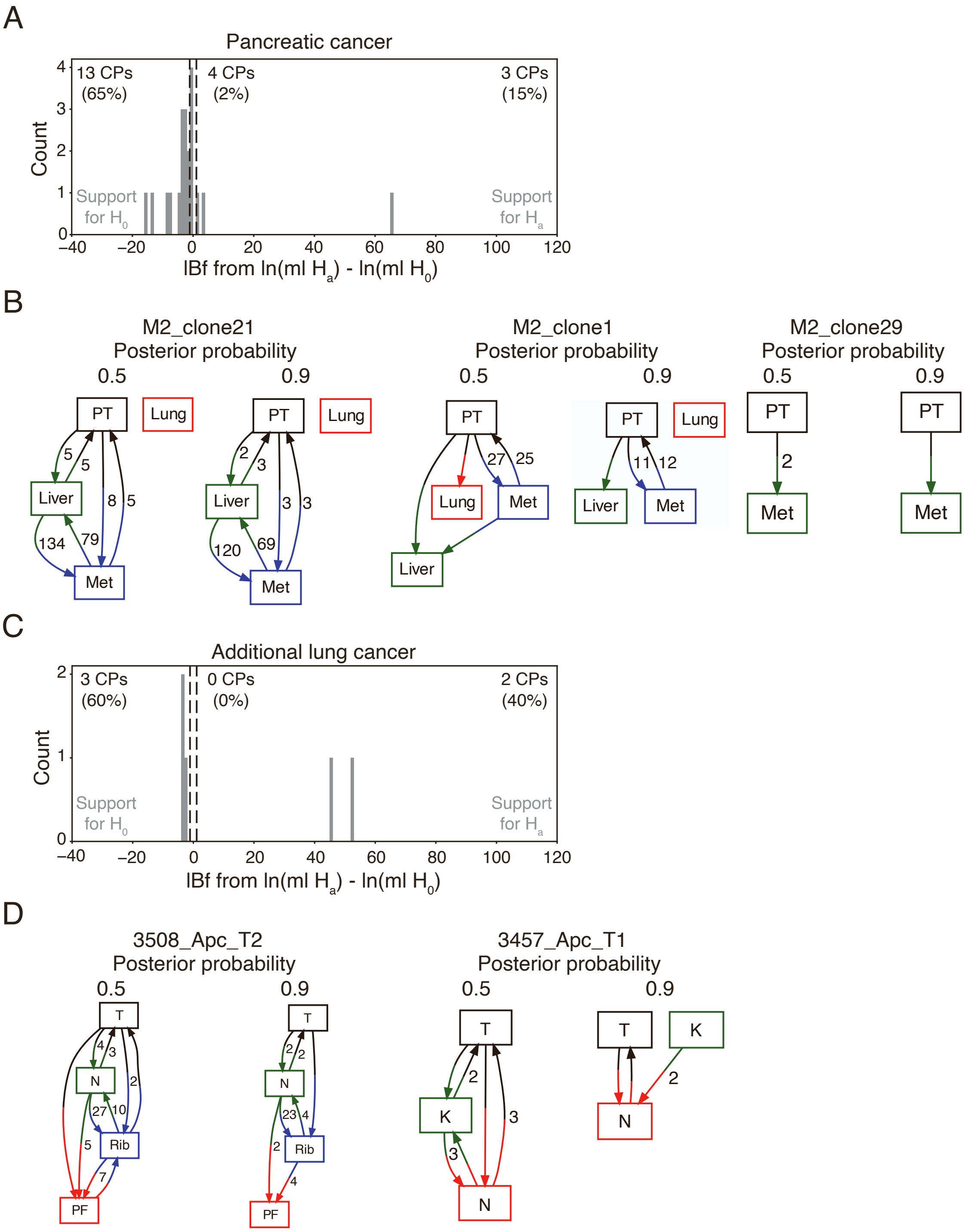

Figure S16. Information-content testing and migration graphs for additional lung and pancreatic cancer datasets, Related to Figure 4

(A) and (C) show the same analysis as in **Figure 4A** for the additional pancreatic[S4] and lung[S3] cancer datasets. (B) and (D) show the 0.5 and 0.9 edgewise probability threshold graphs for those clones passing the tests in (A) and (C) respectively. Passing clones are labeled with their respective IDs in each deposited dataset, which are formatted here as mouse_clone for the pancreatic cancer clones and mouse_genotype_clone for the lung cancer clones. Tissue label acronyms for nodes in the migration graphs are retained from the deposited dataset labels used in the original studies. PT is the primary tissue in (B) and T is the primary tissue in (D).

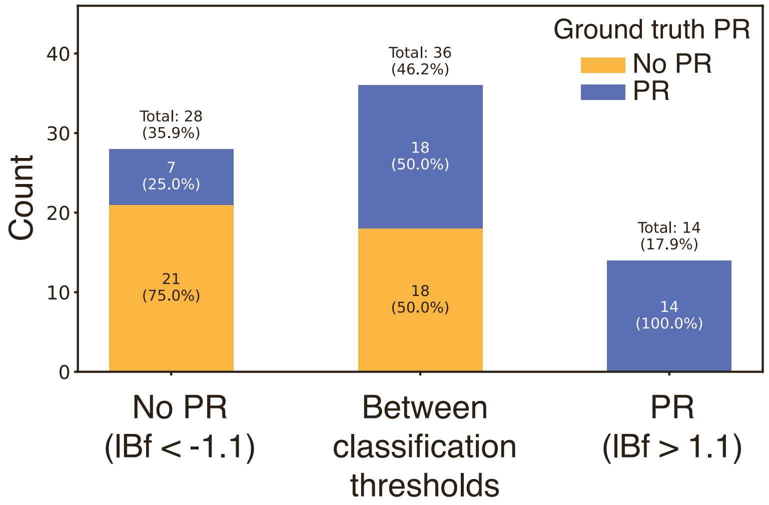

Figure S17. Performance of primary reseeding hypothesis testing on simulated data, Related to Figure 4

Simulated data were classified using a hypothesis test comparing primary reseeding (PR) and no PR models as done for real data in **Figure 4B**. Simulations were selected from the larger variable-rates simulated dataset in **Figure 2B** to include equal numbers of ground truth migration graphs with and without PR. True labels are indicated by color in the legend. Classification results are based on the log Bayes factor (lBf) where simulations are labeled positive for PR if lBF > 1.1, negative for PR if lBF < -1.1, or between classification thresholds if $-1.1 <$ lBF < 1.1.

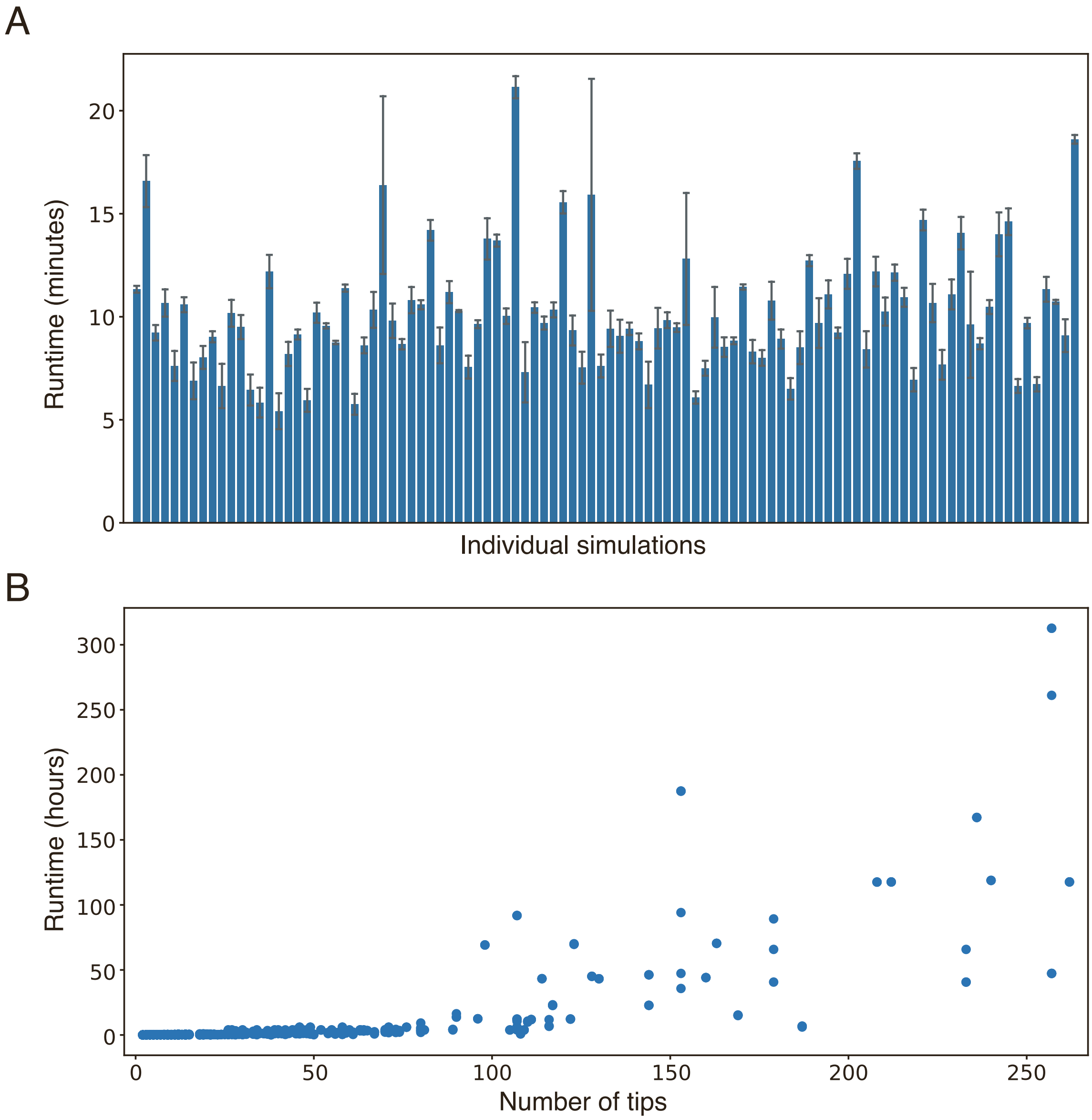

Figure S18. Runtime of BEAM across simulated and real lineage-tracing datasets, Related to Figure 2 and Figure 3
(A) Runtime of BEAM (in minutes) for the 100 simulated datasets shown in **Figure 2A**, each containing 50 tips per tree. Bars show the mean $\pm$ the standard deviation across three independent runs for each simulation. (B) Runtime of BEAM (in hours) for all MCMC chains used in the analysis of the main lung cancer[S3], main prostate cancer[S3], additional pancreatic cancer[S4], and additional lung cancer[S3] datasets in this study, all plotted as a function of the number of tips in each tree. Runtimes were estimated based on the logged runtime per million MCMC samples and the total number of MCMC samples completed at termination. All BEAM analyses were run on a high-performance computing cluster with Intel Xeon 6252, 8260, or 6432 CPU processors. Each job had access to up to five CPU threads (via BEAST 2's native multithreading) and up to 10 GB of memory, though actual memory usage was typically much lower.

SUPPLEMENTARY TABLES

Migration Event	Edgewise Probability Threshold						
	0.5	0.6	0.7	0.8	0.9	0.95	0.99
LL $\rightarrow$ Liv	13	10	8	4	2	0	0
M $\rightarrow$ Liv	27	27	25	25	19	16	9
RL $\rightarrow$ Liv	13	13	10	9	4	2	2

Table S1. Number of liver-directed migration events across posterior probability thresholds, Related to Figure 3
The number of CPs with a transition from left lung (LL), right lung (RL), or mediastinum (M) to Liv at different edgewise posterior-probability thresholds. Counts are out of a total of 35 CPs with the Liv tissue observed.

Mouse	Initial count	GTR selected	Primary reseeding selected
MMUS1469	111	0	0
MMUS1457	24	3	1
MMUS1544	71	1	0
MMUS1495	105	0	0
MMUS1466	17	0	0
MMUS1467	26	0	0
MMUS1492	9	0	0
MMUS1874	23	0	0
MMUS1875	16	0	0
MMUS1588	19	0	0
**Overall count**	421	4	1

Table S2. Per-mouse outcomes of Bayes factor hypothesis testing in prostate cancer, Related to Figure 4

The initial number of total CPs per mouse (left), the number of CPs per mouse selecting the GTR model in the random vs. GTR hypothesis test of data information in **Figure 4A** (middle), and the number of CPs per mouse that selected the primary reseeding (PR) model in the no PR vs. PR hypothesis test in **Figure 4B** (right) out of those that previously selected the GTR model (middle).
