# Supplementary material for: Bayesian inference of tissue-migration histories in metastatic cancer from cell-lineage tracing data
Source: Cell Genom. 2026 Mar 30;6(6):101193. doi: 10.1016/j.xgen.2026.101193 (PMC13261679; doi:10.1016/j.xgen.2026.101193)
Supplement: Document S2. Transparent peer review records for Staklinski et al. [file mmc2.pdf]

Summary

Initial submission: Received : 9/16/25

Scientific editor: Laura Zahn

First round of review: Number of reviewers: 3
Revision invited : 1/7/2026
Revision received : 1/7/2026

Second round of review: Number of reviewers: 3
Accepted : 3/3/2026

Data freely available: Yes

Code freely available: Yes

This transparent peer review record is not systematically proofread, type-set, or edited. Special characters, formatting, and equations may fail to render properly. Standard procedural text within the editor's letters has been deleted for the sake of brevity, but all official correspondence specific to the manuscript has been preserved.

Referees' reports, first round of review

Reviewer #1: In their manuscript "Bayesian inference of tissue-migration histories in metastatic cancer from cell-lineage tracing data", the authors present a novel method called BEAM to infer metastatic seeding histories from single-cell lineage tracing data. This method contributes to a growing field of similar methods towards this aim, advancing over its competitors in two main ways: First, it jointly infers cell lineage trees and their migration histories (existing methods do this sequentially, potentially impairing solution accuracy). Second, BEAM provides a full posterior distribution of lineage trees and tissue-migration graphs rather than simply providing one solution. This could enable users to evaluate conclusions from the data at varying confidence levels and to assess the robustness of these conclusions across a range of solutions.

The authors use simulated data to benchmark BEAM against other methods under a range of biologically plausible mutation rates and migration rates, consistently outperforming other methods, although the degree of improvement depends on the chosen posterior probability threshold. In the latter-half of the manuscript, the authors re-analyze data from metastatic lineage tracing experiments in mice from two published cohorts. One cohort (containing data for 3 lung cancer model mice) had already been re-analyzed in publications for the other tools compared to BEAM in this paper; the other cohort (one prostate cancer model mouse) is itself from the authors of BEAM. Using BEAM, the authors find non-negligible rates of metastasis-to-metastasis seeding and primary-reseeding in lung cancer models, in line with previous studies, although with some differences in the exact frequencies. The authors note an interesting enrichment in mediastinal to right lung (metastasis-to-metastasis) seeding in the three lung cancer mice, and a drastic depletion in lineages with adequate phylogenetic information for migration history inference in the prostate cancer mouse compared to lung cancer. These biological findings provide clear use-cases for BEAM, although they could be similarly obtained using existing methods, and due to the limited data they don't really provide new insights into seeding biology.

The manuscript is clearly written and the analyses presented are logical and sound. The analysis of simulated data suggests that BEAM is a technical advance over existing methods, particularly in the ability to condition inferred migration history graphs on their respective posterior probabilities. I also appreciated the comparison to a simplistic maximum parsimony method to demonstrate BEAM's improvement compared to a strong parsimony assumption. However, beyond this innovation and the technical advance of inferring the lineage tree and migration graph simultaneously, BEAM seems to only modestly improve accuracy compared to existing methods. Considering BEAM is implemented as an extension of the classic method BEAST 2, and that the authors used tools developed by other groups to simulate data used for benchmarking (including the simulator from MACHINA), the paper presents limited new analyses and relatively little empirical data (indeed, of 4 Figures, Fig 4 is quite sparse and could easily go into the supplement and Fig 1 is entirely conceptual). I would have liked to see more data included for re-analysis, surely additional datasets are now available (the lung cancer data dates to 2021, and the prostate cancer data from 2024). Overall, given its methodological advance and the modestly improved accuracy, BEAM will likely find some use in the field. However, given the limited data shown in the paper, the modest gain in accuracy compared to existing methods, and the added challenges to users of working with a full posterior distribution of migration histories rather than a single solution, it may ultimately see limited adoption as newer tools continue to emerge.

Main points:

1. The most interesting potential application of BEAM and similar tools to me (I may be biased) is not to analyze data from CRISPR-based lineage tracing experiments in mice, but rather to infer metastatic seeding histories in human cancer patients. I appreciate the technical limitations in doing this using single cell data given limited coverage and mutation rates, but how would BEAM perform if applied to clones inferred from bulk tumor sequencing from diverse anatomical sites as was done in the MACHINA paper (such as using datasets provided by Gundem, Nature 2015; McPhearson, Nature Genetics, 2016; and other more recent datasets)?

2. I'm curious how BEAM handles biological scenarios that deviate from the simulation framework in unexpected and non-ideal ways. This is very relevant to me as metastasis biology is likely very complex, and benchmarks against overly simplified simulations can be misleading. For example, the simulation methods used seem to enforce very simplistic logic for when migration events occur from each organ: How would BEAM fare against the other methods if migration rates between different pairs of organs systemically differed (as seems to be the case for mediastinal to right lung seeding in the 3 lung cancer mice)? Can variable migration rates between organs actually be estimated using BEAM?

3. Similarly, what if migration rates were time-specific? This could arise as a result of surgical interventions or due to phenotypic changes in response to chemotherapy. Can the authors modify the simulation framework

to drastically decrease or increase seeding from specific organs at certain times and compare BEAM to the other methods in these scenarios?

4. Can the authors provide more information for BEAM's performance compared to other methods when faced with increasing rates of both missing data (i.e., missing values in the character matrices) and miscalled genotypes (incorrect characters)? Having a clear notion of these limitations would help us establish in what other applications BEAM could prove useful (such as with sparse and noisy single cell genotype data, or clones inferred from bulk tissue sequencing as described in my first point).

5. The authors describe that their method performed well despite not explicitly handling poly-clonal seeding, even when their simulations included high rates of poly-clonal seeding events. This is an interest result as my expectation was that seeding by cell clusters could easily lead to incorrect conclusions if not modeled explicitly. Can the authors expand on this more? It was also not clear to me what rates of poly-clonal seeding are being simulated—could the authors please demonstrate BEAM's performance under low/moderate/high rates of poly-clonal seeding?

6. Although it can be a benefit to provide the full range of migration histories and their posterior probabilities in some cases, I can also imagine that this places more onus on users to impose (perhaps arbitrary) confidence thresholds on the tool's output. Considering the broad curves shown in the precision/recall graphs, the user's choice of confidence thresholds will likely have profound impacts on the results, perhaps making the tool less attractive to methods that provide a single optimized solution like MACHINA. Perhaps the authors can provide some practical guidance on how to optimally use BEAM's output when a single migration history is desired.

Minor points:

1. Would it be possible to annotate the 50% posterior probability solution on the precision/recall curves? This would make it easier to compare against methods that provide a single solution, as well as to interpret the full curve.

Reviewer #2: The new method iterates over the phylogenetic tree and likely migration history within a Bayesian framework, identifying possible routes of metastatic spread without relying on a fixed phylogeny. This represents a principled and meaningful methodological advance, especially relevant for high-resolution lineage-tracing datasets.

While a lot of the manuscript emphasizes empirical data (where the ground truth is unknowable), I regard results from simulated datasets as a more reliable baseline for performance evaluation. Figure 2B illustrates that BEAM performs slightly better than MACHINA, Metient, and MACH2, but the overall gain is quite modest. In fact, a significant p-value is not particularly informative here, as significance can always be achieved by increasing the number of simulations.

Additionally, many of the precision-recall results in the supplementary figures show that previous methods cluster near the midrange of BEAM, indicating that BEAM's advantage may be limited. A major concern is that the comparisons are mainly limited to parsimony-based methods, which are known to be less accurate than Bayesian approaches like PathFinder. Since PathFinder has already demonstrated improved performance over these parsimony methods, it is important to include it in the benchmarking analysis. Without such a comparison, it remains uncertain whether BEAM genuinely advances the state of the art in migration inference.

Reviewer #3: In the study titled “Bayesian inference of tissue-migration histories in metastatic cancer from cell-lineage tracing data” by Staklinski and colleagues, the authors present an elegant computational method for inferring metastatic migration histories from single-cell lineage tracing datasets. The authors first present a concise background on the problem of inferring metastatic histories and deficits in previous inference methods. They then describe their method, BEAM, which is a Bayesian method for inferring a migration history by jointly modeling the cell lineage history and migration graph. They largely use simulations to benchmark their method against other methods and present new analyses of publicly-available datasets from CRISPR-based lineage-tracing datasets and propose a novel and principled hypothesis-testing framework for assessing migration models.

Overall, BEAM is an elegant method that will be of interest to the field of cancer researchers using single-cell lineage-tracing approaches. The methodology is principled, and the authors solidly demonstrate the

advantages of a Bayesian approach. At the same time, the authors should strengthen the present study with the following suggestions:

Major Comments:

1. The benchmarking simulations can be clarified and expanded:
    a. First, the underlying assumptions of migration likelihoods are a bit unclear. Looking at the Methods section, they authors describe how they simulate migrations, specifically: "… [migrating cells are] migrated to a destination tissue chosen from a uniform transition matrix across ten tissues". It appears that this assumes that all transitions between any one of 10 sites are uniform – but this is not always the case (for example, the LL to RL or Liver examples discussed below). Would simulating from a variety of different transition matrices, not all uniform, change the findings of the study? It will be important to look at examples where some transition probabilities are 0 (e.g., as with the hypothesis testing at the end).
    b. Second, in the previous methods tested, the authors describe that they first reconstruct a phylogeny (using LAML) and then infer migration histories. This is reasonable, and realistic, but I am curious if the performance differential observed is due to inaccuracies in tree estimation or flaws in the migration history inference. Perhaps a supplemental figure showing the performance of MACHINA and other benchmark methods, assuming the ground truth phylogeny is known, would be helpful for users to understand the failure modes.
    c. Third, and relatedly, it will be helpful to know to what extent the number of possible tissues affects the performance of BEAM and other methods. Specifically, are there meaningful performance differences in inferring the migration graph between 2 vs 10 vs 20 tissues?
    d. Fourth, can the authors comment on how the lineage tracing simulation parameters relate to estimates from experimental data?

2. The authors note that computational complexity is a tradeoff in using BEAM, due to its MCMC sampling approach. This is understandable, and a well-known limitation of Bayesian approaches. However, I suggest that the authors still present an analysis of CPU time and memory consumption for some of the real data (or simulated data, whichever is easiest) to give readers a sense of the computational requirements of this method.

3. The authors bring up an interesting point around the number of migration-informative mutations being important for inference (Figure S8). This is a major limitation in many realistic scenarios since metastasis is a late-occurring event and will likely not have many mutations associated with it (either because of target site exhaustion or little time between sampling and metastasis). There are two opportunities here that will be useful to the field:
    a. First, can the authors present an analysis of how the number of metastasis-informative mutations affects the inference of migration graphs? This is similar to the benchmarking performed in Figure 2C, but specifically the question is how many lineage-informed edges in the ground-truth tree coincide with migration events.
    b. Second, the authors should show an analysis of the KP-Tracer dataset described in Yang et al. It's understandable that there are few phylogenetically-informative mutations, but this is a relatively well-used dataset in several studies (including Chu et al [PMID: 40604857] and Butler et al [https://doi.org/10.1098/rspb.2024.2850]) and can be helpful for contextualizing results. If an issue is scalability, perhaps only evaluating unique cell lineage states can reduce the dataset size and allow BEAM to run?

4. One major advantage of BEAM is that it models the posterior distribution of all possible migration graphs, and migration graphs can be ranked by probability. The authors do a nice job of presenting a range of results depending on the probability threshold, but it's unclear what the best practices are. Based on simulations, can the authors provide a bit more rationale as to what is the most reasonable posterior-probability consensus threshold to use?

5. The machinery proposed in this paper may be useful for a variety of problems, not just migration inference. For example, some recent papers have proposed using graph-labeling approaches to infer cell-state hierarchies from phylogeneis (including Yang et al [PIMD: 35523183], Schiffman et al [PMID: 39317739] and Sashittal et al [PMID: 39314473]). This is an important problem with far-reaching applications in the context of lineage-tracing, beyond cancer. Commenting on, or perhaps even showing, the applicability of BEAM to the problem of learning cell differentiation maps would broaden the scope of this paper.

Minor comments:

1. Figure 1 can be improved with a bit more detail. For example, what are the rows/columns/colors in the character matrix? What do "P", "M1", etc represent specifically?
2. The markings in Figure 2C are quite small – I suggest you increase the size of the 'X' marks representing the performance of different methods.
3. The discussion around parsimony approaches "oversimplifying the true history" of migration events (p. 8) can be misleading. The whole purpose of parsimony approaches is to oversimplify, as this is assumed to the most likely model. Below, when analyzing the tissue migration histories in experimental data, it is unclear if having more or fewer migrations is actually advantageous. Perhaps it is most fair to say that parsimony assumptions break down when the migration rates are high, which can be the case in late-stage aggressive disease.

Together, this is an excellent study and I am supportive of its publication after considering these comments, which I hope will serve to improve the scope and rigor of the paper.

Authors' response to the first round of review

We thank the editor for the opportunity to revise our manuscript, and the reviewers for their careful evaluation of our work and their thoughtful and constructive comments. We were pleased that the reviewers appreciated the innovation of our work, the rigor of our methods, our extensive simulation study, and the potential usefulness of BEAM to the emerging community focused on the study of metastasis via lineage-tracing data. At the same time, they highlighted a number of limitations and opportunities for improvement in our study, which have led us to substantially expand our analyses of both simulated and real data. Altogether, we have now doubled the number of fully analyzed metastatic CRISPR lineage-tracing datasets from two to four, providing what we believe to be the first comprehensive analysis spanning all currently available cancer datasets of this type. We also performed an extensive new benchmarking effort that, for the first time, evaluates every existing method in the field side-by-side. In response to suggestions from the reviewers, we generated 500 new simulations to address a wide range of issues (e.g., heritable missing data, migration timing, and M-hub behavior), which are summarized in seven new supplementary figures and major updates to a main figure. We believe that, together, these additions have greatly improved our manuscript and that it will be of broad interest to readers of Cell Genomics .

We provide point-by-point responses to all reviewer comments below.

Reviewers' Comments :

Reviewer #1: In their manuscript "Bayesian inference of tissue-migration histories in metastatic cancer from cell-lineage tracing data", the authors present a novel method called BEAM to infer metastatic seeding histories from single-cell lineage tracing data. This method contributes to a growing field of similar methods towards this aim, advancing over its competitors in two main ways: First, it jointly infers cell lineage trees and their migration histories (existing methods do this sequentially, potentially impairing solution accuracy). Second, BEAM provides a full posterior distribution of lineage trees and tissue-migration graphs rather than simply providing one solution. This could enable users to evaluate conclusions from the data at varying confidence levels and to assess the robustness of these conclusions across a range of solutions.

We appreciate these comments. We do wish to highlight also our extension to Bayesian hypothesis testing, which we believe is a particularly novel feature of the paper, and one that addresses an important need in the community.

The authors use simulated data to benchmark BEAM against other methods under a range of biologically plausible mutation rates and migration rates, consistently outperforming other methods, although the degree of improvement depends on the chosen posterior probability threshold. In the latter-half of the manuscript, the authors re-analyze data from metastatic lineage tracing experiments in mice from two published cohorts. One cohort (containing data for 3 lung cancer model mice) had already been re-analyzed in publications for the other tools compared to BEAM in this paper; the other cohort (one prostate cancer model mouse) is itself from the authors of BEAM.

Using BEAM, the authors find non-negligible rates of metastasis-to-metastasis seeding and primary-reseeding in lung cancer models, in line with previous studies, although with some differences in the exact frequencies. The authors note an interesting enrichment in mediastinal to right lung (metastasis-to-metastasis) seeding in the three lung cancer mice, and a drastic depletion in lineages with adequate phylogenetic information for migration history inference in the prostate cancer mouse compared to lung cancer. These biological findings provide clear use-cases for BEAM, although they could be similarly obtained using existing methods, and due to the limited data they don't really provide new insights into seeding biology.

We acknowledge the limitations of our analysis of real data, but note that the field is moving quickly and more informative data sets will almost certainly emerge in the near future (as mentioned in our Discussion). We view this manuscript, in large part, as setting the methodological stage for data sets that are not yet available but soon will be.

The manuscript is clearly written and the analyses presented are logical and sound. The analysis of simulated data suggests that BEAM is a technical advance over existing methods, particularly in the ability to condition inferred migration history graphs on their respective posterior probabilities. I also appreciated the comparison to a simplistic maximum parsimony method to demonstrate BEAM's improvement compared to a strong parsimony assumption. However, beyond this innovation and the technical advance of inferring the lineage tree and migration graph simultaneously, BEAM seems to only modestly improve accuracy compared to existing methods. Considering BEAM is implemented as an extension of the classic method BEAST 2, and that the authors used tools developed by other groups to simulate data used for benchmarking (including the simulator from MACHINA), the paper presents limited new analyses and relatively little empirical data (indeed, of 4 Figures, Fig 4 is quite sparse and could easily go into the supplement and Fig 1 is entirely conceptual). I would have liked to see more data included for re-analysis, surely additional datasets are now available (the lung cancer data dates to 2021, and the prostate cancer data from 2024). Overall, given its methodological advance and the modestly improved accuracy, BEAM will likely find some use in the field. However, given the limited data shown in the paper, the modest gain in accuracy compared to existing methods, and the added challenges to users of working with a full posterior distribution of migration histories rather than a single

solution, it may ultimately see limited adoption as newer tools continue to emerge.
Main points:
1. The most interesting potential application of BEAM and similar tools to me (I may be biased) is not to analyze data from CRISPR-based lineage tracing experiments in mice, but rather to infer metastatic seeding histories in human cancer patients. I appreciate the technical limitations in doing this using single cell data given limited coverage and mutation rates, but how would BEAM perform if applied to clones inferred from bulk tumor sequencing from diverse anatomical sites as was done in the MACHINA paper (such as using datasets provided by Gundem, Nature 2015; McPhearson, Nature Genetics, 2016; and other more recent datasets)?
We agree with the reviewer that a long-term goal of this general research area should be to develop methods that can be applied to data from human patients, but unfortunately, applications to bulk tumor sequencing data are well beyond the scope of the current manuscript. The current version of BEAM is designed for discrete sequence evolution of individual cells, whereas clonal phylogeny inference from bulk data relies on deconvolving variant allele frequency matrices to estimate subclonal genotypes and proportions (for examples and discussion of such approaches, see Gillis and Roth [PMID: 33302872], Deshwar et al. [PMID: 25786235], and Kulman et al. [PMID: 39775053]). These frequency-based mixture models differ fundamentally from the DNA mutation models in BEAST2 that underlie BEAM's barcode-editing and migration framework. At the same time, high-resolution lineage tracing in model systems enables uncovering biological insights that are difficult or impossible to obtain from human tumors. Human data are often confounded by treatment and limited sampling, whereas model systems allow dense lineage reconstruction, temporal control, and causal testing of how genotype, treatment, and microenvironment shape metastatic spread. We therefore view BEAM as addressing fundamental questions that are most appropriately studied in model systems, while also providing a conceptual foundation for future extensions as human data and methodologies continue to advance. We have added a brief statement in the Discussion to highlight some of the remaining challenges that must be addressed before models of this kind can be applied to data from human patients.
2. I'm curious how BEAM handles biological scenarios that deviate from the simulation framework in unexpected and non-ideal ways. This is very relevant to me as metastasis biology is likely very complex, and benchmarks against overly simplified simulations can be misleading. For example, the simulation methods used seem to enforce very simplistic logic for when migration events occur from each organ: How would BEAM fare against the other methods if migration rates between different pairs of organs systemically differed (as seems to be the case for mediastinal to right lung seeding in the 3 lung cancer mice)? Can variable migration rates between organs actually be estimated using BEAM?
We appreciate the reviewer's point about evaluating BEAM under heterogeneous migration scenarios and now provide results in Sup. Fig. 12 to demonstrate that BEAM remains effective when migration rates differ systematically between tissues. Specifically, we simulated data in which one metastatic tissue acted as a migration hub

with elevated outgoing migration rates, matching the asymmetric patterns observed in the real lung-cancer data. In this heterogeneous-rate regime, BEAM again outperformed the other methods, with its inferred relative migration rates accurately recovering the underlying M-hub generative structure. It is of course impossible to examine how the available methods will compare under all possible tissue-migration scenarios, but this experiment at least demonstrates that BEAM's advantages are not restricted to cases of equal migration rates between organs. Indeed, we expect that the flexibility of our tissue migration model should generally result in improvements in relative performance over parsimony-based methods in more complex scenarios.

3. Similarly, what if migration rates were time-specific? This could arise as a result of surgical interventions or due to phenotypic changes in response to chemotherapy. Can the authors modify the simulation framework to drastically decrease or increase seeding from specific organs at certain times and compare BEAM to the other methods in these scenarios?

To attempt to shed light on this scenario, we extended our simulations to include short, time-localized bursts of migration to examine how BEAM responds to temporally heterogeneous migration, although current lineage-tracing datasets have not been designed to address this question. We found that brief migration bursts increased the number of tissues seeded and primarily produced primary-to-metastasis events, rather than more complex metastasis-to-metastasis or reseeding patterns. Consistent with this simpler structure, parsimony-based methods performed comparably to BEAM when migration was dominated by a short primary dissemination pulse. We therefore focused on migration timing and observed that BEAM had somewhat increased sensitivity for elevated densities of migration events within the true migration window than the LAML/Fitch-Hartigan parsimony approach. However, the posterior distributions remained quite broad for these smaller simulated datasets, so we share these results here rather than making any stronger claims in the paper. These results highlight both the utility of BEAM for detecting time-localized migration and the limits of temporal resolution given the uncertainty in the data and the assumption of a strict molecular clock.

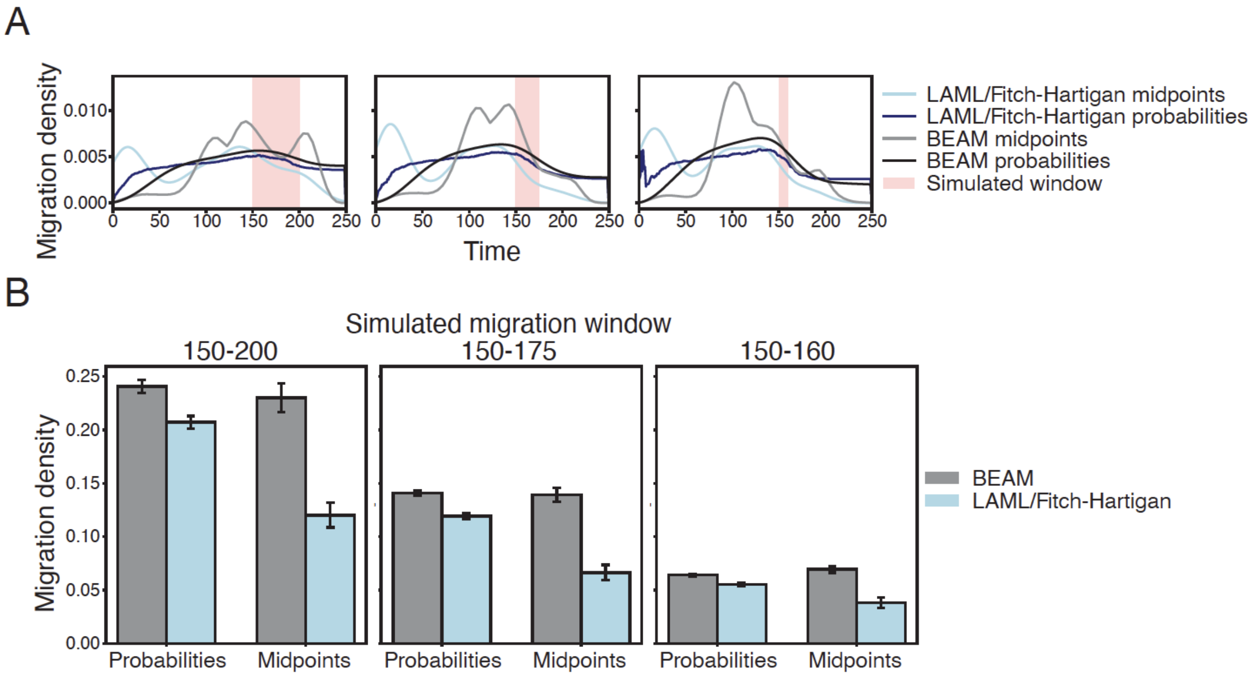

Figure caption: A. Migration time estimates from LAML/Fitch--Hartigan parsimony and BEAM for simulations under three different windows of time (150-200, 150-175, 150-160) in which migrations were allowed to occur. We ran 50 simulations for each window with data simulated under a migration rate of 1 x 10^{-4} and a mutation rate of 0.0025. For each inferred migration event, we computed either a midpoint (the average parent--child time) or a probability measure in which the entire event interval for the branch in the tree is given uniform weight. Shaded red regions indicate the true simulated windows. B. Mean migration density recovered within each true window across all replicates for both midpoint and probability migration event representations. Bars are shown +/- standard error of the mean across replicate simulations.

4. Can the authors provide more information for BEAM's performance compared to other methods when faced with increasing rates of both missing data (i.e., missing values in the character matrices) and miscalled genotypes (incorrect characters)? Having a clear notion of these limitations would help us establish in what other applications BEAM could prove useful (such as with sparse and noisy single cell genotype data, or clones inferred from bulk tissue sequencing as described in my first point).

We agree that evaluating robustness to missing or noisy data is important and that our original simulations did not directly model barcode dropout at considerable levels. We have now simulated barcode data under increasing heritable silencing rates that introduce higher levels of missing entries. These new results, in Sup. Fig. 7, show that BEAM maintains some baseline level of performance even when up to 90 percent of barcode sites are missing, likely because it can draw on tissue labels when mutation data are sparse. In contrast, methods that rely on a phylogeny inferred solely from mutation data show a substantial drop in performance under these conditions. This is expected because the resulting phylogeny would impose constraints that require many

more migrations to explain the tissue distribution at the tips under parsimony-based inference. We did not model miscalled genotypes, which are less emphasized in the field and likely less problematic for indel-based barcodes, where indels are easier to call than single-nucleotide variants. But we expect that such noise would have a similar effect, leading to reduced performance for all methods with BEAM remaining comparatively more robust due to its use of tissue-label information.

5. The authors describe that their method performed well despite not explicitly handling poly-clonal seeding, even when their simulations included high rates of poly-clonal seeding events. This is an interest result as my expectation was that seeding by cell clusters could easily lead to incorrect conclusions if not modeled explicitly. Can the authors expand on this more? It was also not clear to me what rates of poly-clonal seeding are being simulated—could the authors please demonstrate BEAM's performance under low/moderate/high rates of poly-clonal seeding?

We are very interested in explicitly studying polyclonal seeding and see this as an important direction for future work. In our current simulations, however, polyclonality is not directly parameterized but emerges naturally from the stochastic dynamics of the simulated cancer population and we have limited ability to control it. As defined in Sup. Fig. 6 , co-migration corresponds to distinct tissue-to-tissue edges in the ground-truth migration graph, and seeding is classified as monoclonal or polyclonal based on the presence of multi-edges, making polyclonality an emergent property, rather than a tunable parameter. Explicitly controlling polyclonality would require a substantial redesign of our simulation framework which we believe is beyond the scope of the current manuscript.

6. Although it can be a benefit to provide the full range of migration histories and their posterior probabilities in some cases, I can also imagine that this places more onus on users to impose (perhaps arbitrary) confidence thresholds on the tool's output. Considering the broad curves shown in the precision/recall graphs, the user's choice of confidence thresholds will likely have profound impacts on the results, perhaps making the tool less attractive to methods that provide a single optimized solution like MACHINA. Perhaps the authors can provide some practical guidance on how to optimally use BEAM's output when a single migration history is desired.

We agree that practical guidance is important. Based on our simulations, we now recommend two simple posterior thresholds: 0.50 for exploratory, higher-recall summaries and 0.90 for more conservative, high-precision results. We have added markers at these thresholds to the precision-recall plots to help readers calibrate the sensitivity-precision tradeoff, and we include this guidance in the revised Discussion .

Minor points:

1. Would it be possible to annotate the 50% posterior probability solution on the precision/recall curves? This would make it easier to compare against methods that provide a single solution, as well as to interpret the full curve.

We have annotated the 0.50 and 0.90 posterior probability thresholds on all precision-recall plots, reflecting our recommended guidelines and enabling clear comparison to single-solution methods across the simulated conditions.

Reviewer #2: The new method iterates over the phylogenetic tree and likely migration

history within a Bayesian framework, identifying possible routes of metastatic spread without relying on a fixed phylogeny. This represents a principled and meaningful methodological advance, especially relevant for high-resolution lineage-tracing datasets.

We appreciate these comments.

While a lot of the manuscript emphasizes empirical data (where the ground truth is unknowable), I regard results from simulated datasets as a more reliable baseline for performance evaluation. Figure 2B illustrates that BEAM performs slightly better than MACHINA, Metient, and MACH2, but the overall gain is quite modest. In fact, a significant p-value is not particularly informative here, as significance can always be achieved by increasing the number of simulations.

We initially presented F1 scores and p-values as concise summaries for consistency with prior work, but on further consideration, we believe that the F1 scores are of limited value here and the precision-recall curves, now annotated with BEAM point estimates, are far more informative. In addition, our F1 scores summarized only one set of simulation results, which were ill-suited for differentiating among methods because all methods performed quite well in this idealized setting. The full set of analyses demonstrates that BEAM's advantages become more evident under more challenging conditions, including not only higher mutation and migration rates (bottom right of Fig 2B) but also degraded input tree quality (Fig. 2A; Sup. Fig. 3) and high levels of heritable barcode missingness (Sup. Fig. 7). Accordingly, we have moved the F1-score plots and p-values to Sup. Fig. 3 . More broadly, we wish to emphasize that, beyond its performance on these benchmarks, BEAM introduces a fundamentally different approach to migration graph inference from the parsimony-based methods that have dominated the field, by directly modeling the uncertainty in tissue-migration histories, jointly considering cell-lineage phylogenies and migration graphs, and enabling Bayesian hypothesis testing of higher-level properties of migration histories.

Additionally, many of the precision-recall results in the supplementary figures show that previous methods cluster near the midrange of BEAM, indicating that BEAM's advantage may be limited. A major concern is that the comparisons are mainly limited to parsimony-based methods, which are known to be less accurate than Bayesian approaches like PathFinder. Since PathFinder has already demonstrated improved performance over these parsimony methods, it is important to include it in the benchmarking analysis. Without such a comparison, it remains uncertain whether BEAM genuinely advances the state of the art in migration inference.

We have now included PathFinder in Fig. 2 . We initially attempted to include PathFinder in our study and we consulted the authors regarding interpretation of its outputs. They advised us that PathFinder is designed to return a single best solution rather than a calibrated posterior distribution as expected from a Bayesian approach, and, to our surprise, recommended that we use MACHINA instead. Nonetheless, we have now re-included PathFinder in our main simulated-data benchmarks. We do find, indeed, that PathFinder lacks interpretable probabilistic output and performs fairly poorly on our simulated datasets, which may explain its omission from other recent benchmarking studies.

In another effort to ensure fair benchmarking, we incorporated feedback from the authors of Metient who informed us of updates to their default parsimony weights that were made during their own peer review process. We have now used these new defaults. They also confirmed our observation that Metient-calibrate does not improve over Metient-evaluate for cell lineage tracing data. We also now present Metient results integrated over multiple output solutions and shown as a curve on the precision-recall plots alongside BEAM and MACH2, as requested by the authors. Overall, we believe that our updated manuscript represents a good-faith attempt at fair and comprehensive benchmarking of the state-of-the-art methods in this field.

Reviewer #3: Comments enter in this field will be shared with the author; your identity will remain anonymous.

In the study titled "Bayesian inference of tissue-migration histories in metastatic cancer from cell-lineage tracing data" by Staklinski and colleagues, the authors present an elegant computational method for inferring metastatic migration histories from single-cell lineage tracing datasets. The authors first present a concise background on the problem of inferring metastatic histories and deficits in previous inference methods. They then describe their method, BEAM, which is a Bayesian method for inferring a migration history by jointly modeling the cell lineage history and migration graph. They largely use simulations to benchmark their method against other methods and present new analyses of publicly-available datasets from CRISPR-based lineage-tracing datasets and propose a novel and principled hypothesis-testing framework for assessing migration models.

Overall, BEAM is an elegant method that will be of interest to the field of cancer researchers using single-cell lineage-tracing approaches. The methodology is principled, and the authors solidly demonstrate the advantages of a Bayesian approach.

We appreciate these comments. We have endeavored to convey the value of the Bayesian approach through a variety of applications (e.g., sampling graphs, thresholding edges by confidence, and performing hypothesis testing) to make it concrete for non-statistical readers. We have found that the idea of properly accounting for uncertainty can come across as abstract and obscure for some biologists unless it is very well motivated.

At the same time, the authors should strengthen the present study with the following suggestions:

Major Comments:

1. The benchmarking simulations can be clarified and expanded:

a. First, the underlying assumptions of migration likelihoods are a bit unclear.

Looking at the Methods section, they authors describe how they simulate migrations, specifically: "… [migrating cells are] migrated to a destination tissue chosen from a uniform transition matrix across ten tissues". It appears that this assumes that all transitions between any one of 10 sites are uniform – but this is not always the case (for example, the LL to RL or Liver examples discussed below). Would simulating from a variety of different transition matrices, not all uniform, change the findings of the study? It will be important to look at examples where some transition probabilities are 0 (e.g., as with the hypothesis testing at the end).

We agree that it is useful to consider less restricted migration rates. As described in our response to Reviewer #1, we have now added Sup. Fig. 12 , which examines scenarios with systematically heterogeneous migration rates, including zero-probability transitions reflecting the asymmetric patterns observed in the lung cancer data. Under these conditions, BEAM again outperformed other methods and accurately recovered the underlying structured migration patterns, demonstrating robustness to biologically motivated heterogeneity.

b. Second, in the previous methods tested, the authors describe that they first reconstruct a phylogeny (using LAML) and then infer migration histories. This is reasonable, and realistic, but I am curious if the performance differential observed is due to inaccuracies in tree estimation or flaws in the migration history inference. Perhaps a supplemental figure showing the performance of MACHINA and other benchmark methods, assuming the ground truth phylogeny is known, would be helpful for users to understand the failure modes.

We thank the reviewer for this insightful suggestion. In our simulation setting, it is straightforward to fix the phylogeny at the true value, and as the reviewer suggests, a comparison of this case with inferred trees is a natural and elegant way to disentangle tree reconstruction from migration inference. In response, we have carried out a series of experiments of this kind by repurposing the 100 idealized simulated data in Fig. 2 that were produced under a mutation rate of 0.0025 and migration rate of 1 x 10 $^{-6}$ *. For each method requiring an input tree, we evaluated three conditions: a parsimony tree inferred by Cassiopeia-greedy, a maximum-likelihood tree inferred by LAML, and the ground-truth simulation tree, which removes CRISPR tree reconstruction error. These results are now included in the main Fig. 2 . We additionally report mean normalized Robinson–Foulds distances between inferred and true trees for Cassiopeia-greedy, LAML, and BEAM, showing the expected improvements in tree accuracy, with BEAM performing best overall. Together, these results demonstrate that parsimony-based methods are highly sensitive to input tree quality, whereas BEAM is able to mitigate this limitation by jointly inferring the tree and migration history. We believe this addition strengthens the manuscript by further emphasizing the importance of the joint inference strategy.*

c. Third, and relatedly, it will be helpful to know to what extent the number of possible tissues affects the performance of BEAM and other methods. Specifically, are there meaningful performance differences in inferring the migration graph between 2 vs 10 vs 20 tissues?

The number of tissues adds an important layer of complexity to migration-history inference. Although our simulations allowed up to 10 tissues, stochastic growth and migration naturally generated a wide range of realized tissue counts, enabling us to assess performance as a function of tissue number without additional simulations. We included a new Sup. Fig. 6C plot, which groups simulations by realized tissue count and reports precision and recall for all methods. As expected, methods performed similarly when migration histories were simple (<3 tissues), but as tissue number and graph complexity increased, BEAM showed a growing advantage over parsimony-based approaches. We did not extend this analysis beyond 10 tissues, as

that is already at the top of the range observed in available cancer metastasis datasets.

d. Fourth, can the authors comment on how the lineage tracing simulation parameters relate to estimates from experimental data?

Our simulations used mutation rates calibrated to match experimental lineage-tracing data, while migration rates were explored across a broad range to capture plausible behaviors. Sup. Fig. 13 shows that simulated mutational diversity at our primarily chosen mutation rate of 0.0025 most closely reflects the highest quality of published CRISPR lineage-tracing datasets in cancer metastasis. Migration rates could not be directly estimated from real data since migration histories are latent, so we instead explored diverse migration regimes and provided representative graphs to enable qualitative comparisons. We added a sentence to the Discussion to suggest that readers align their new datasets to these simulated regimes.

2. The authors note that computational complexity is a tradeoff in using BEAM, due to its MCMC sampling approach. This is understandable, and a well-known limitation of Bayesian approaches. However, I suggest that the authors still present an analysis of CPU time and memory consumption for some of the real data (or simulated data, whichever is easiest) to give readers a sense of the computational requirements of this method.

We have added Sup. Fig. 18 reporting BEAM runtimes for both simulated datasets and the real datasets analyzed in the study. Since direct measurements of memory usage were not available from previously executed runs, we have included in the figure caption the amount of memory (10 GB per job) conservatively allocated for each BEAM run as a practical reference for resource requirements. We have also added a citation to recent work from our group demonstrating the promise of variational inference for scalable Bayesian phylogenetics, providing a practical path towards improved scalability of BEAM.

3. The authors bring up an interesting point around the number of migration-informative mutations being important for inference (Figure S8). This is a major limitation in many realistic scenarios since metastasis is a late-occurring event and will likely not have many mutations associated with it (either because of target site exhaustion or little time between sampling and metastasis). There are two opportunities here that will be useful to the field:

a. First, can the authors present an analysis of how the number of metastasis-informative mutations affects the inference of migration graphs? This is similar to the benchmarking performed in Figure 2C, but specifically the question is how many lineage-informed edges in the ground-truth tree coincide with migration events.

We added Sup. Fig. 5 quantifying metastasis-informative mutations, defined as mutations occurring on migration branches in the true tree, across all variable-rate simulation conditions. Their abundance increased with mutation and migration rates and peaked in the same regimes where BEAM showed its largest performance gains, indicating that improved accuracy arises from stronger migration-linked phylogenetic signals. Although this metric is informative for simulations, it is inherently circular for real data because it relies on knowledge of the true tree. This limitation motivated our development of a Bayes-factor measure of migration-history information directly based

on observed data, which captures both direct and indirect phylogenetic constraints, helping users assess when migration inference is likely to be reliable.

b. Second, the authors should show an analysis of the KP-Tracer dataset described in Yang et al. It's understandable that there are few phylogenetically-informative mutations, but this is a relatively well-used dataset in several studies (including Chu et al [PMID: 40604857] and Butler et al [https://doi.org/10.1098/rspb.2024.2850]) and can be helpful for contextualizing results. If an issue is scalability, perhaps only evaluating unique cell lineage states can reduce the dataset size and allow BEAM to run?

We analyzed both the KP-Tracer lung cancer (Yang et al.) and pancreatic cancer (Simeonov et al.) datasets in the new Sup. Fig. 16 . As shown previously in Sup. Fig. 13 , these datasets have limited mutational content, which we now find leads to high uncertainty in both tree reconstruction and migration inference. Because this limitation mirrors the conclusions drawn from our prostate cancer analyses, we present these results concisely in the supplementary figures. Rather than over-interpreting such data, we emphasize that many existing lineage-tracing datasets lack sufficient phylogenetic signals to support robust and unbiased reconstruction of migration histories.

4. One major advantage of BEAM is that it models the posterior distribution of all possible migration graphs, and migration graphs can be ranked by probability. The authors do a nice job of presenting a range of results depending on the probability threshold, but it's unclear what the best practices are. Based on simulations, can the authors provide a bit more rationale as to what is the most reasonable posterior-probability consensus threshold to use?

As described in our response to Reviewer #1, we now clearly define recommended liberal (0.50) and conservative (0.90) thresholds. We mark them on the precision-recall curves for our simulation results for reference.

5. The machinery proposed in this paper may be useful for a variety of problems, not just migration inference. For example, some recent papers have proposed using graph-labeling approaches to infer cell-state hierarchies from phylogenies (including Yang et al [PIMD: 35523183], Schiffman et al [PMID: 39317739] and Sashittal et al [PMID: 39314473]). This is an important problem with far-reaching applications in the context of lineage-tracing, beyond cancer. Commenting on, or perhaps even showing, the applicability of BEAM to the problem of learning cell differentiation maps would broaden the scope of this paper.

We agree, in principle, that BEAM's Bayesian framework can be extended to other problems involving dynamic processes on uncertain lineage trees. However, we believe that inferring full cell differentiation hierarchies would require modeling unobserved intermediate (stem-cell like) states, which are not supported in the current implementation, and therefore we believe demonstrating applicability to this problem is beyond the scope of the current manuscript. Nevertheless, we have added text to the Discussion exploring this interesting topic.

Minor comments:

1. Figure 1 can be improved with a bit more detail. For example, what are the rows/columns/colors in the character matrix? What do "P", "M1", etc represent specifically?

We have revised the Fig. 1 appearance and caption to clarify that the character matrix represents the observed barcode mutation states across single cells (rows = cells, columns = barcode mutation sites, colors = edit states). We also explain that "P" denotes the primary tumor and "M1–M6" represent metastatic tumors.

2. The markings in Figure 2C are quite small – I suggest you increase the size of the 'X' marks representing the performance of different methods.

We have increased the size of the "X" markers in precision/recall plots.

3. The discussion around parsimony approaches "oversimplifying the true history" of migration events (p. 8) can be misleading. The whole purpose of parsimony approaches is to oversimplify, as this is assumed to be the most likely model. Below, when analyzing the tissue migration histories in experimental data, it is unclear if having more or fewer migrations is actually advantageous. Perhaps it is most fair to say that parsimony assumptions break down when the migration rates are high, which can be the case in late-stage aggressive disease.

Our intent was not to criticize parsimony approaches for their simplifying assumptions but rather to highlight the parameter regimes in which those assumptions fail to approximate the underlying process. As suggested, we have revised the text to clarify that "parsimony assumptions tend to break down" when migration rates are high or mutation information is sparse.

Together, this is an excellent study and I am supportive of its publication after considering these comments, which I hope will serve to improve the scope and rigor of the paper.

We thank the reviewer for this support and for these constructive suggestions.

Referees' reports, second round of review

Reviewer #1: I would like to thank the authors for their revised manuscript and thoughtful point-by-point response to my comments regarding their original submission. The revisions made address my concerns about BEAM's performance given imbalanced seeding rates across tissues, and missing genotype data. I also appreciate the revised discussion in which the authors provide some practical guidance on how to interpret BEAM's output, such as the 0.5 and 0.9 posterior probability thresholds, and their annotation of these values on all the precision-recall plots. Generally, I feel this manuscript would have been substantially stronger had the authors developed their own simulation framework rather than reusing existing methods, as this would have given the authors more flexibility to explore different seeding scenarios. As such, I still don't have a good sense of what parameter regimes pose limits to BEAM's performance. For example, despite their efforts in Figure S6D, polyclonal seeding is obviously not a binary phenomenon; how many clones seeded from the primary to a specific metastasis (and at what relative frequencies) pose an upper limit to BEAM's performance? That said, I respect that developing a novel simulation framework is non-trivial and outside the scope of the current work. This ranting is merely in the hopes that future work will consider using a de novo simulation framework that enables more complex and realistic biological scenarios to be considered. I have no further comments and support the publication of the manuscript without further revision, and I would like to again thank the authors.

Reviewer #2: Authors have addressed my concerns adequately.

Reviewer #3: I would like to commend the authors of the manuscript entitled "Bayesian inference of tissue-migration histories in metastatic cancer from cell-lineage tracing data" on formulating a comprehensive response and strong revision. They have addressed all of my concerns and I have no additional suggestions. I am in favor for its acceptance and believe this will be of great interest to the field of researchers using single-cell lineage tracing approaches.

Authors' response to the second round of review

Reviewer #1: I would like to thank the authors for their revised manuscript and thoughtful point-by-point response to my comments regarding their original submission. The revisions made address my concerns about BEAM's performance given imbalanced seeding rates across tissues, and missing genotype data. I also appreciate the revised discussion in which the authors provide some practical guidance on how to interpret BEAM's output, such as the 0.5 and 0.9 posterior probability thresholds, and their annotation of these values on all the precision-recall plots. Generally, I feel this manuscript would have been substantially stronger had the authors developed their own simulation framework rather than reusing existing methods, as this would have given the authors more flexibility to explore different seeding scenarios. As such, I still don't have a good sense of what parameter regimes pose limits to BEAM's performance. For example, despite their efforts in Figure S6D, polyclonal seeding is obviously not a binary phenomenon; how many clones seeded from the primary to a specific metastasis (and at what relative frequencies) pose an upper limit to BEAM's performance? That said, I respect that developing a novel simulation framework is non-trivial and outside the scope of the current work. This ranting is merely in the hopes that future work will consider using a de novo simulation framework that enables more complex and realistic biological scenarios to be considered. I have no further comments and support the publication of the manuscript without further revision, and I would like to again thank the authors.

We have now added text addressing this remaining point in the new "Limitations of the study" section of the Discussion.

Reviewer #2: Authors have addressed my concerns adequately.

Reviewer #3: I would like to commend the authors of the manuscript entitled "Bayesian inference of tissue-migration histories in metastatic cancer from cell-lineage tracing data" on formulating a comprehensive response and strong revision. They have addressed all of my concerns and I have no additional suggestions. I am in favor for its acceptance and believe this will be of great interest to the field of researchers using single-cell lineage tracing approaches.

We appreciate the positive feedback from all three reviewers and thank them for their time and careful consideration of our work.
